# MiR-196b-3p and miR-450b-3p are key regulators of adipogenesis in porcine intramuscular and subcutaneous adipocytes

**DOI:** 10.1186/s12864-023-09477-0

**Published:** 2023-06-27

**Authors:** Wenjing Wu, Keke Liu, Zhongyu You, Jin Zhang

**Affiliations:** 1 College of Biological and Chemical Engineering, Jiaxing University, Jiaxing Zhejiang, 314000 China; 2 College of Agronomy and Biotechnology, Hebei Normal University of Science and Technology, Qin Huangdao Hebei, 066000 China

**Keywords:** Pig, Adipocyte, RNA-seq, MiR-196b-3p, MiR-450b-3p, Adipogenesis

## Abstract

**Background:**

As components of white adipose tissue, porcine intramuscular (IM) and subcutaneous (SC) adipocytes undergo similar differentiation and adipogenesis processes. However, the adipogenic capacity of IM adipocytes is weaker than that of SC adipocytes. Identifying key regulators underlying this difference between IM and SC adipocytes will benefit pig breeding.

**Results:**

In this study, we used BGISEQ-500 sequencing technology to analyze the expression of small RNAs in primary cultured IM and SC adipocytes on day 8 after adipogenic induction, and found 32-fold higher miR-196b-3p expression, as well as 8-fold lower miR-450b-3p expression in IM adipocytes than in SC adipocytes. Functional studies revealed that miR-196b-3p inhibits adipogenesis by targeting *CD47* via the AMPK signaling pathway, and its effect was attenuated by the specific p-AMPKα activator AICAR. We also found that miR-450b-3p promotes adipogenesis by targeting *SIRT1* via the Wnt/β-catenin signaling pathway, and its effect was weakened by the Wnt/β-catenin signaling activator LiCl.

**Conclusions:**

Our findings suggest that miR-196b-3p and miR-450b-3p are novel key regulatory factors that play opposite roles in porcine adipogenesis, helping us decipher the regulatory differences between porcine IM and SC fat deposition.

**Supplementary Information:**

The online version contains supplementary material available at 10.1186/s12864-023-09477-0.

## Introduction

Fat deposition is an important economic trait of pigs. The intramuscular (IM) fat content is the key factor of pork quality, while subcutaneous (SC) fat deposition is negatively associated with the lean mass ratio of the carcass [1]. Accordingly, improving the IM fat content or decreasing SC fat deposition are major goals of pig breeding. As components of white adipose tissue, IM and SC adipocytes exhibit similar differentiation and adipogenesis processes [2]. However, the adipogenic capacity of IM adipocytes is significantly weaker and their triglyceride content is lower than that of SC adipocytes [3]. Identifying key regulators and unveiling the difference in adipogenesis between IM and SC adipocytes will benefit pig breeding.

Adipogenesis is mediated by a series of complex mechanisms, including commitment of mesenchymal stem cells to differentiate into preadipocytes, and the induction of preadipocyte differentiation into mature adipocytes [4]. The whole adipogenic lineage progression is orchestrated by a set of genes, among which fatty acid synthase (*FAS*), fatty acid binding protein 4 (*aP2*), peroxisome proliferator-activated receptor γ (*PPARγ*), and CCAAT enhancer binding proteins (*C/EBPs*) are considered adipogenic marker genes due to their dominant roles [5–8]. By regulating these adipogenic marker genes, signaling cascades such as the Wnt/β-catenin and AMPK/SIRT1 pathways determine the adipogenic capacity of porcine IM and SC adipocytes by sensing intercellular ligands. In the Wnt/β-catenin signaling pathway, Wnt can affect adipocyte differentiation based on β-catenin-dependent (canonical Wnt) and β-catenin-independent (non-canonical Wnt) mechanisms, among which β-catenin plays a central role in inhibiting adipogenesis [9, 10]. AMP-activated protein kinase (AMPK) is a fuel-sensing enzyme that is activated by an increased AMP/ATP ratio [7]. AMPK activation can inhibit the expression of downstream adipocyte differentiation transcription factors, including *PPARγ*, *C/EBPα*, and *SREBP-1* [7, 11]. Subsequently, the expression of lipogenic genes regulated by these transcription factors is also suppressed, including acetyl-CoA carboxylase (*ACC*), *FAS* and *aP2* [12], leading to the inhibition of adipocyte differentiation.

In the past decade, microRNAs (miRNAs) have been proved to be pivotal regulators in the adipogenic network, and differentially expressed miRNAs between porcine IM and SC adipocytes have been identified with high throughput technologies [13–15]. However, the signaling pathways and mechanisms through which miRNAs affect porcine adipogenesis need further exploration.

In this study, based on miRNA sequencing analysis of porcine IM and SC adipocytes, we discovered that the expression of miR-196b-3p in IM adipocytes was significantly higher than in SC adipocytes, while the expression of miR-450b-3p was higher in SC adipocytes than in IM adipocytes, after which the effects and mechanism of miR-196b-3p and miR-450b-3p on adipogenesis in adipocytes were explored. The results revealed that miR-196b-3p inhibits adipogenesis through the AMPK pathway by targeting *CD47*, while miR-450b-3p promotes adipogenesis via the WNT pathway by targeting Sirtuin 1 (*SIRT1*). Our findings suggest that miR-196b-3p and miR-450b-3p are novel key regulatory factors that play opposite roles in porcine adipogenesis.

## Results

### The expression levels of miR-196b-3p and miR-450b-3p were significantly different between porcine intramuscular (IM) and subcutaneous (SC) adipocytes

**Fig. 1:**
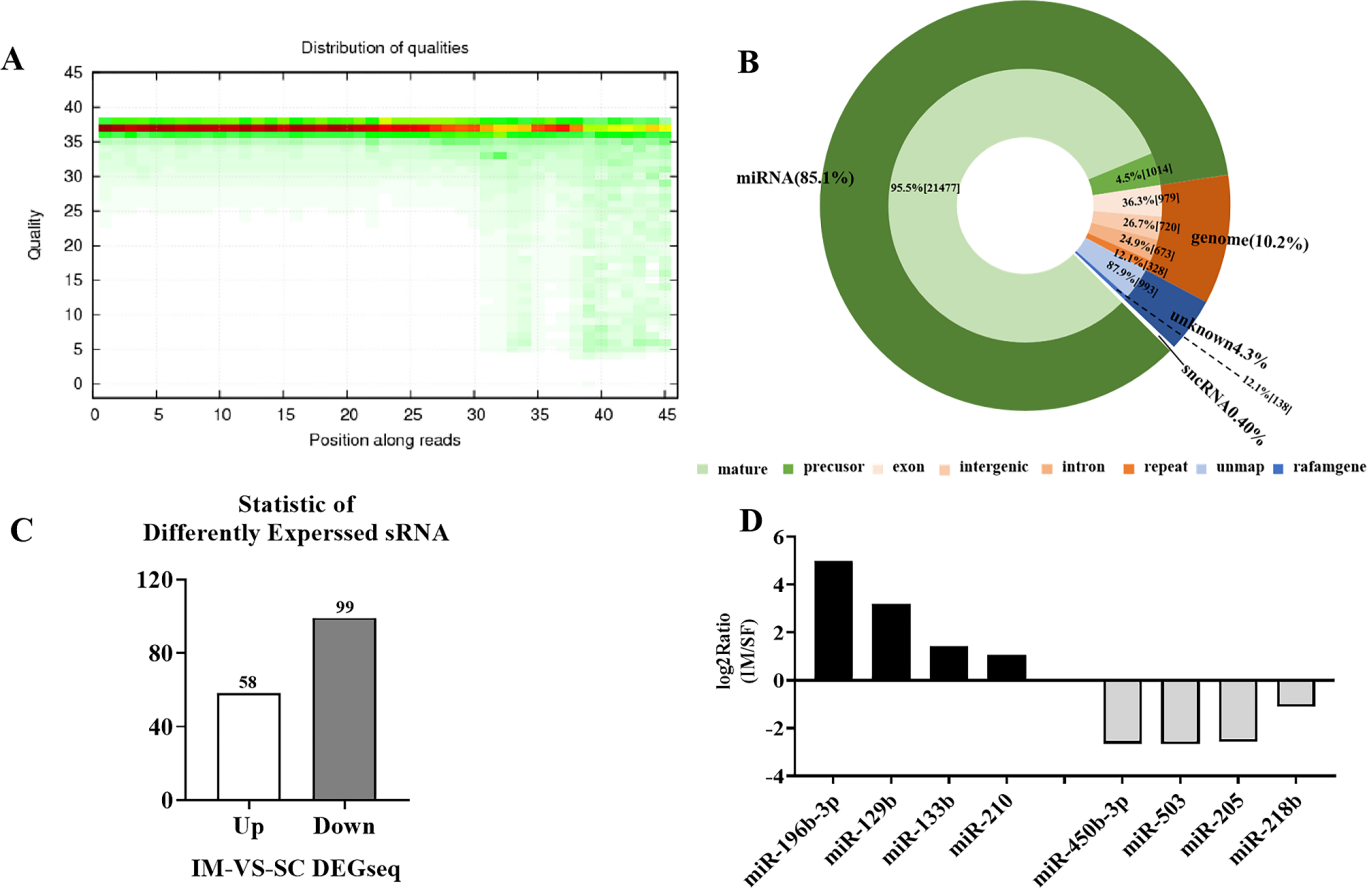
The distribution of miRNAs in IM and SC adipocytes. **A** Quality of miRNAs. **B** Relative proportions of different sRNAs. **C-D** Significantly differentially expressed (SDE) miRNAs between IM and SC adipocytes

To explain the difference in adipogenic capacity between IM and SC adipocytes, the expression of small RNAs in primary cultured IM and SC adipocytes on day 8 post adipogenic induction was determined using the BGISEQ-500 sequencing platform. The primary cultured adipocytes were from three Jiaxing black pigs and the number of non-coding small RNAs detected in each sample (mainly miRNAs and piRNAs) is shown in Supplementary Table 1. The base quality distribution of clean tags is presented in 35–40, indicating that the sequencing quality is good (Fig. 1A). Alignment and annotation analysis revealed that miRNAs accounted for most of the reads (Fig. 1B). A comparison of the miRNA expression profiles of IM and SC adipocytes revealed 157 differentially expressed miRNAs, 58 of which were upregulated, while 99 were downregulated (Fig. 1C). Among them, ssc-miR-196b-3p (log2Ratio (IM / SC) = 5.0) and ssc-miR-450b-3p (log2Ratio (IM / SC) = -2.6) showed the highest relative abundance and the largest fold change (Fig. 1D). These results indicate that miR-196b-3p and miR-450b-3p may play a pivotal role in porcine adipogenesis and their functions are related to the different adipogenic capacity of IM and SC adipocytes.

### MiR-196b-3p and miR-450b-3p inhibit adipocyte proliferation

**Fig. 2:**
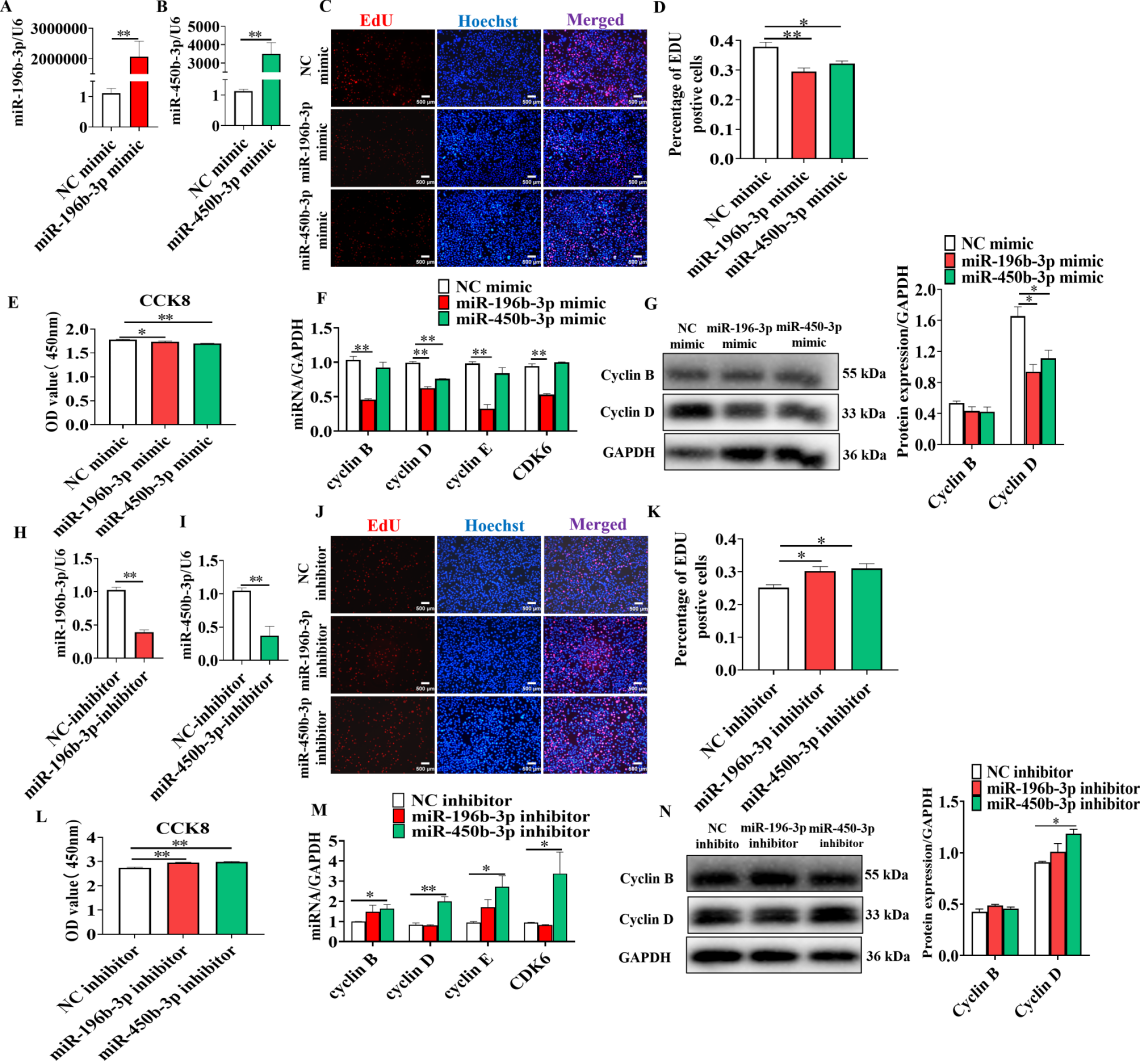
MiR-196b-3p and miR-450b-3p inhibited the proliferation of adipocytes. Adipocytes were transfected with miR-196b-3p or miR-450b-3p mimics for 24 h (**A-B**). **C** The proliferation of adipocytes was examined using the EdU assay. Red represents EdU staining, and blue represents cell nuclei counter-stained with Hoechst 33,342. **D** The percentage of EdU-positive cells was quantified. **E** Cell proliferation was examined using the CCK-8 assay. **F-G** The expression levels of cyclin B, cyclin D, cyclin E and CDK6 were determined by real-time quantitative PCR and western blot analysis. Adipocytes were transfected with miR-196b-3p or miR-450b-3p inhibitor for 24 h (**H-I**). **J-L** The proliferation of adipocytes was examined using EdU staining and the CCK-8 assay. **M-N** The expression levels of cyclin B, cyclin D, cyclin E and CDK6. The grouping of blots cropped from different parts of the same gel. The data represent the means ± SEM. n = 3, *P < 0.05, **P < 0.01

To understand the role of miR-196b-3p and miR-450b-3p in adipocyte proliferation, 3T3-L1 cells were transfected with mimics or the negative control (NC). The mimics markedly increased the expression of miR-196b-3p and miR-450b-3p in 3T3-L1 cells (Fig. 2A&B). EdU staining indicated that miR-196b-3p and miR-450b-3p decreased the proportion of S-phase cells (Fig. 2C&D). The cell counting assay (CCK-8) showed that miR-196b-3p and miR-450b-3p decreased the numbers of viable cells (Fig. 2E). Gene expression analysis showed that miR-196b-3p suppressed the expression of the cell cycle marker genes *cyclin B*, *cyclin D*, *cyclin E* and *CDK6*, while miR-450b-3p suppressed *cyclin D* (Fig. 2F&G).

To further confirm the results, the miR-196b-3p and miR-450b-3p inhibitors were used to transfect 3T3-L1 cells. Following transfection, the expression of miR-196b-3p and miR-450b-3p was significantly decreased (Fig. 2H&I). The EdU staining assay revealed that the miR-196b-3p and miR-450b-3p inhibitors increased the proportion of S-Phase cells (Fig. 2J&K). The cell counting assay (CCK-8) showed that the miR-196b-3p and miR-450b-3p inhibitors increased the number of viable cells (Fig. 2L). Gene expression analysis showed that miR-196b-3p upregulated *cyclin B*, while the miR-450b-3p inhibitor upregulated *cyclin B*, *cyclin D*, *cyclin E* and *CDK6* (Fig. 2M&N). Taken together, the results showed that miR-196b-3p and miR-450b-3p inhibited adipocyte proliferation.

### MiR-196b-3p inhibits, while miR-450b-3p promotes adipocyte differentiation

**Fig. 3:**
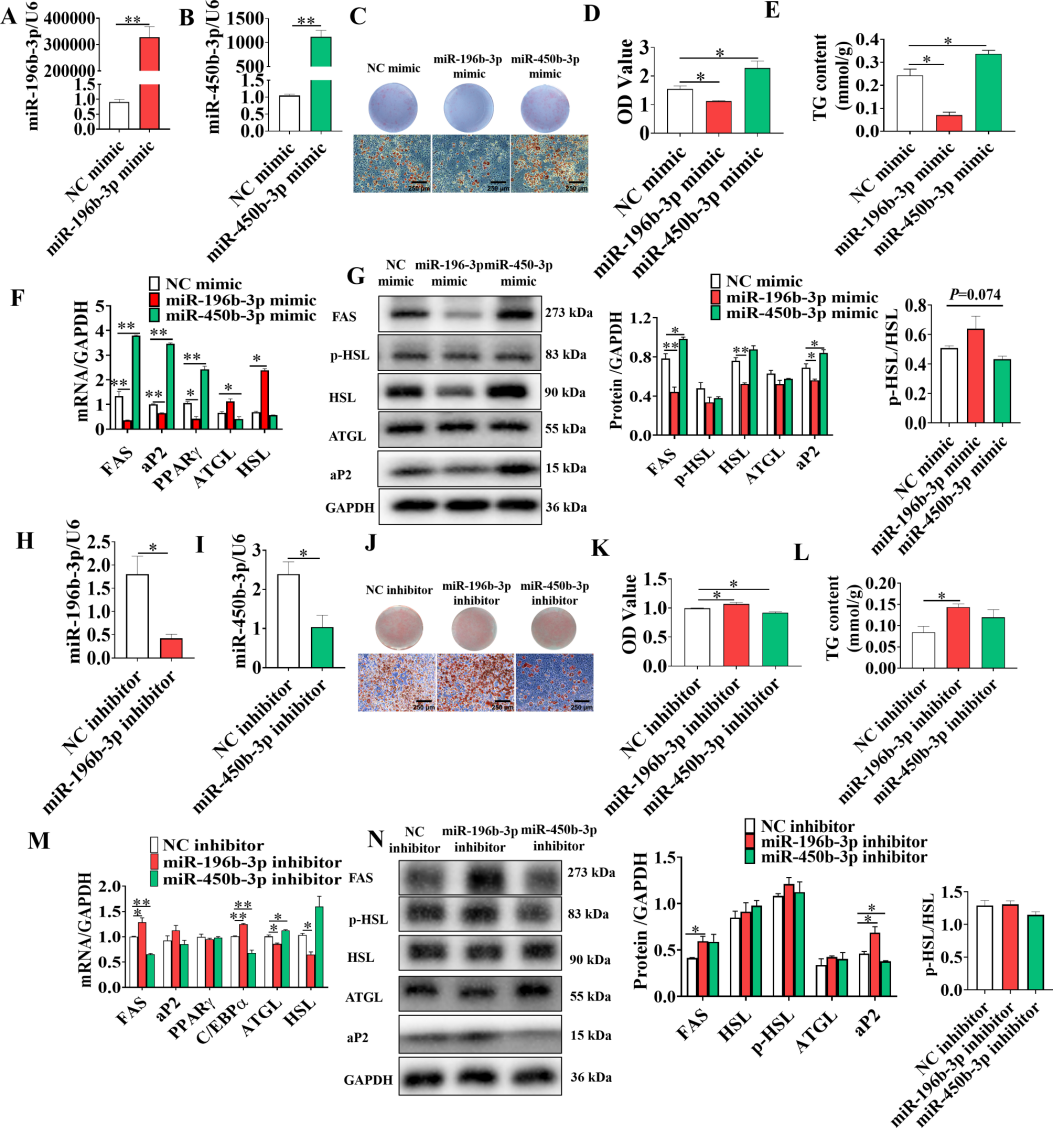
MiR-196b-3p inhibited and miR-450b-3p promoted lipid accumulation in 3T3-L1 adipocytes. After transfection with miR-196b-3p or miR-450b-3p mimics and induction of adipogenic differentiation, the overexpression efficiency of miRNAs was confirmed (**A-B**). **C** 3T3-L1 adipocytes were stained with oil red O at day 10. The intracellular lipid content was determined by oil red O staining (**D**), as well as by measuring the triglyceride content in adipocytes (**E**). **F** mRNA levels of *FAS*, *aP2, PPARγ*, *ATGL* and *HSL* according to real-time qPCR analysis. **G** Protein levels of adipogenic markers after transfection and induction of adipogenic differentiation for 10 days. Before 24 h of induction of differentiation with the cocktail method, 3T3-L1 adipocytes were transfected with miR-196b-3p or miR-450b-3p inhibitor. **H-I** The interference efficiency of miRNA was confirmed. **J-L** The adipocyte formation and triglyceride content of 3T3-L1 adipocytes was detected by oil red O staining. **M-N** After differentiation, the expression levels of adipogenic transcription factors were determined by real-time quantitative PCR, and protein levels were assessed by western blot analysis. The grouping of blots cropped from different parts of the same gel. The data represent the means ± SEM. n = 3, *P < 0.05, **P < 0.01

After transfection with mimics, miR-196b-3p and miR-450b-3p were markedly upregulated on day 8 after adipogenic induction (Fig. 3A&B). Oil Red O staining showed that miR-196b-3p mimics led to a decrease of the intracellular lipid content by approximately 30% (Fig. 3C&D) and similar results were obtained using a commercial triglyceride kit (Fig. 3E). Gene expression analysis showed that miR-196b-3p mimics significantly downregulated either the mRNA or the protein level of adipogenic markers, including *FAS*, *aP2* and *PPARγ* (Fig. 3F&G). However, the miR-450b-3p mimics led to increases of intracellular lipid content by about 50% (Fig. 3C&E). Gene expression analysis showed that miR-450b-3p mimic significantly induced both the mRNA and protein levels of adipogenic genes, including *FAS*, *aP2* and *PPARγ* (Fig. 3F&G).

To further confirm the results, the miR-196b-3p and miR-450b-3p inhibitors were used to transfect 3T3-L1 cells. The expression of miR-196b-3p and miR-450b-3p decreased significantly (Fig. 3H&I). Both oil red O staining and triglyceride content analysis showed that miR-196b-3p inhibitor led to an increase of the intracellular lipids content (Fig. 3J, K&L). Gene expression analysis showed that miR-196b-3p inhibitor dramatically upregulated adipogenic genes (*FAS*, *C/EBPα* and *aP2*) and downregulated lipolysis-related genes (*ATGL* and *HSL*) (Fig. 3M&N). However, mir-450b-3p inhibitor led to a decrease of the intracellular triglyceride content (Fig. 3J, K&L). Gene expression analysis showed that miR-450b-3p inhibitor significantly downregulated adipogenic genes (*FAS* and *C/EBPα*) and upregulated lipolysis-related genes (*ATGL*) (Fig. 3M&N). Taken together, these results indicate that miR-196b-3p and miR-450b-3p play opposite roles in adipogenesis and lipolysis.

### MiR-196b-3p activates the AMPKα signal pathway, while miR-450b-3p inhibits the WNT signaling pathway

**Fig. 4:**
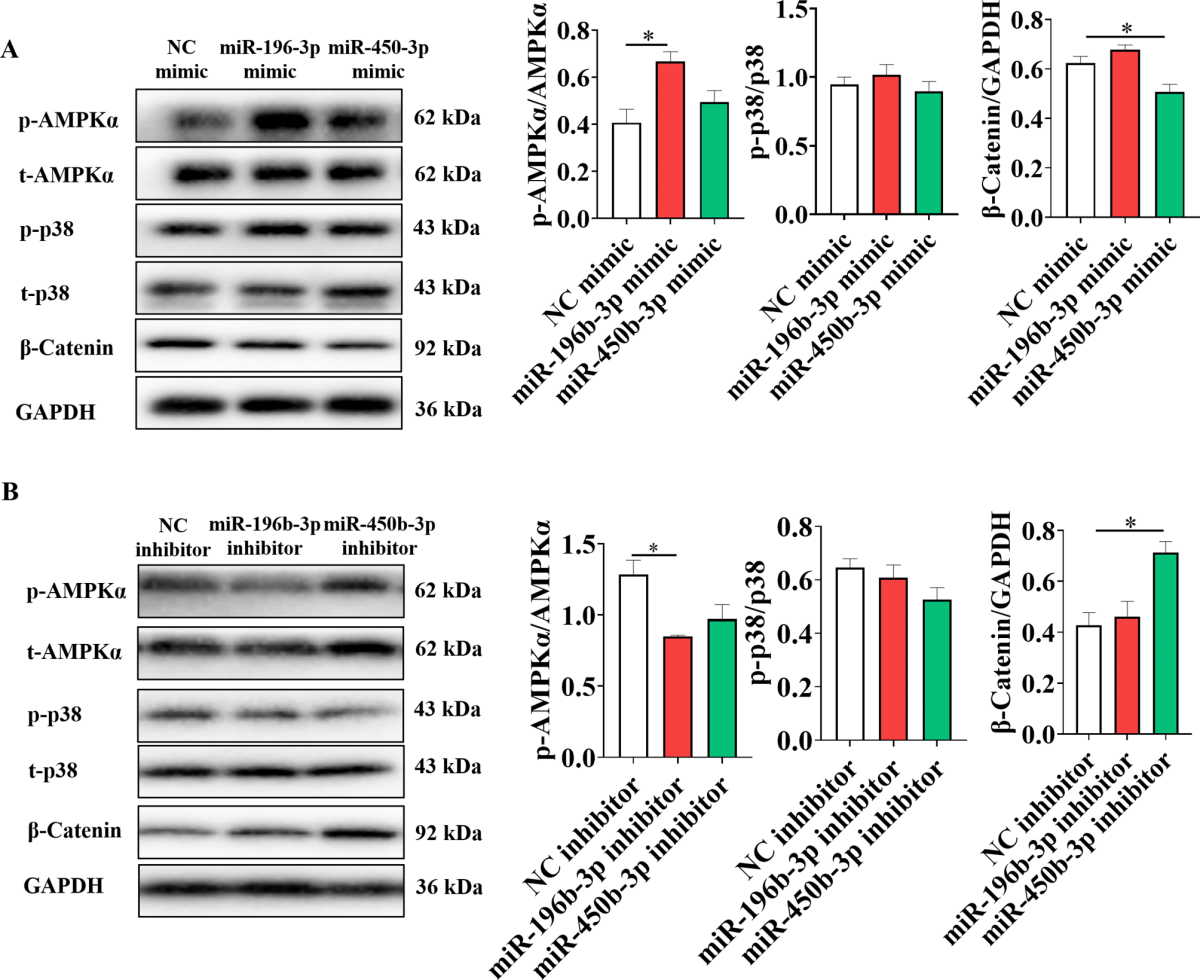
MiR-196b-3p regulates adipogenesis via AMPK signaling and miR-450b-3p regulates adipogenesis via β-Catenin/WNT signaling in adipocytes. After overexpression (**A**) and interference (**B**) of miR-196b-3p and miR-450b-3p, the protein levels of p-AMPK, p-p38 and β-catenin were investigated by western blot analysis. Quantification was performed densitometrically using ImageJ. The grouping of blots cropped from different parts of the same gel. The data were expressed as means ± SEM. n = 3, *P < 0.05, **P < 0.01

In order to understand the potential mechanisms through which miR-196b-3p and miR-450b-3p affect adipogenesis, proteins involved in the AMPKα, Wnt/β-catenin and p38MAPK signaling pathways were analyzed. The results showed that miR-196b-3p mimic increased the levels of phosphorylated AMPKα (p-AMPKα), and miR-196b-3p inhibitor decreased p-AMPKα (Fig. 4A&B). However, there were no significant changes in the other pathways. Similarly, miR-450b-3p mimic decreased the β-catenin level and miR-450b-3p inhibitor increased the β-catenin level (Fig. 4A&B), but there were no significant effects on other pathways.

**Fig. 5:**
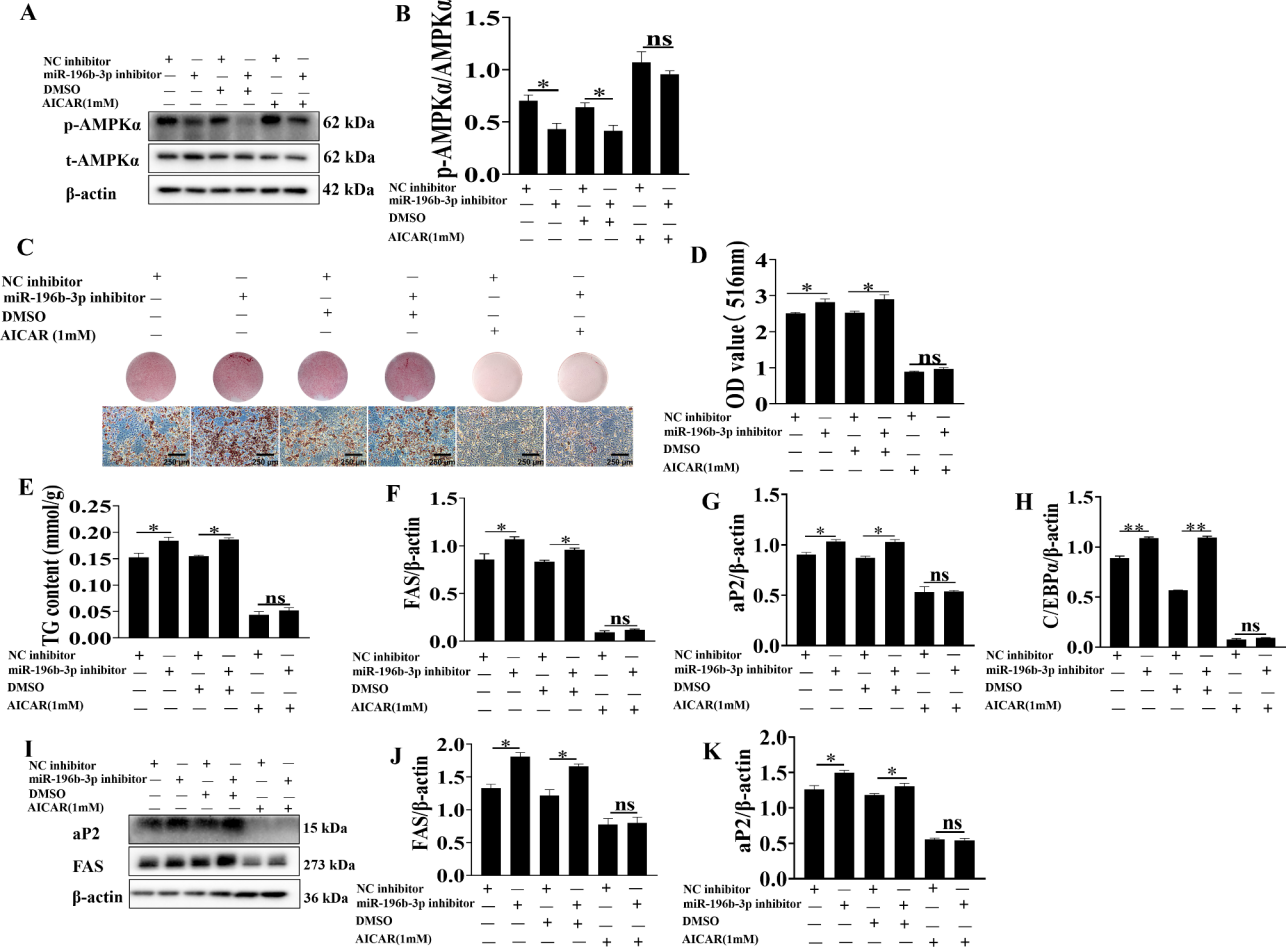
Inhibition of miR-196b-3p was rescued by AICAR. After transfected with miR-196b-3p inhibitor, 3T3-L1 adipocytes were treated with AICAR. **A-B** The levels of phosphorylated AMPKα were determined. **C-E** The intracellular lipid content and triglyceride content were examined. **F-H** Real-time qPCR analysis of FAS, aP2, C/EBPα. **I-K** Western blot analysis of the protein expression of the adipogenic markers FAS and aP2. The grouping of blots cropped from different parts of the same gel. The data represent the means ± SEM. n = 3, *P < 0.05, **P < 0.01

To further confirm and validate the pathway analysis results, the AMP-activated protein kinase (AMPK) activator, 5-aminoimidazole-4-carboxamide ribonucleoside (AICAR), was added to 3T3-L1 adipocytes transfected with miR-196b-3p inhibitor. The upregulation of p-AMPKα by miR-196b-3p inhibitor was restored by AICAR (Fig. 5A&B). Oil Red O staining and TG content analysis showed that the promotion of adipogenesis by miR-196b-3p inhibitor was attenuated by AICAR (Fig. 5C-E). AICAR downregulated the expression of adipogenic markers (*FAS*, *aP2* and *C/EBPα*), which indicated that activation of AMPK eliminated the effects of miR-196b-3p inhibitor (Fig. 5F-K).

**Fig. 6:**
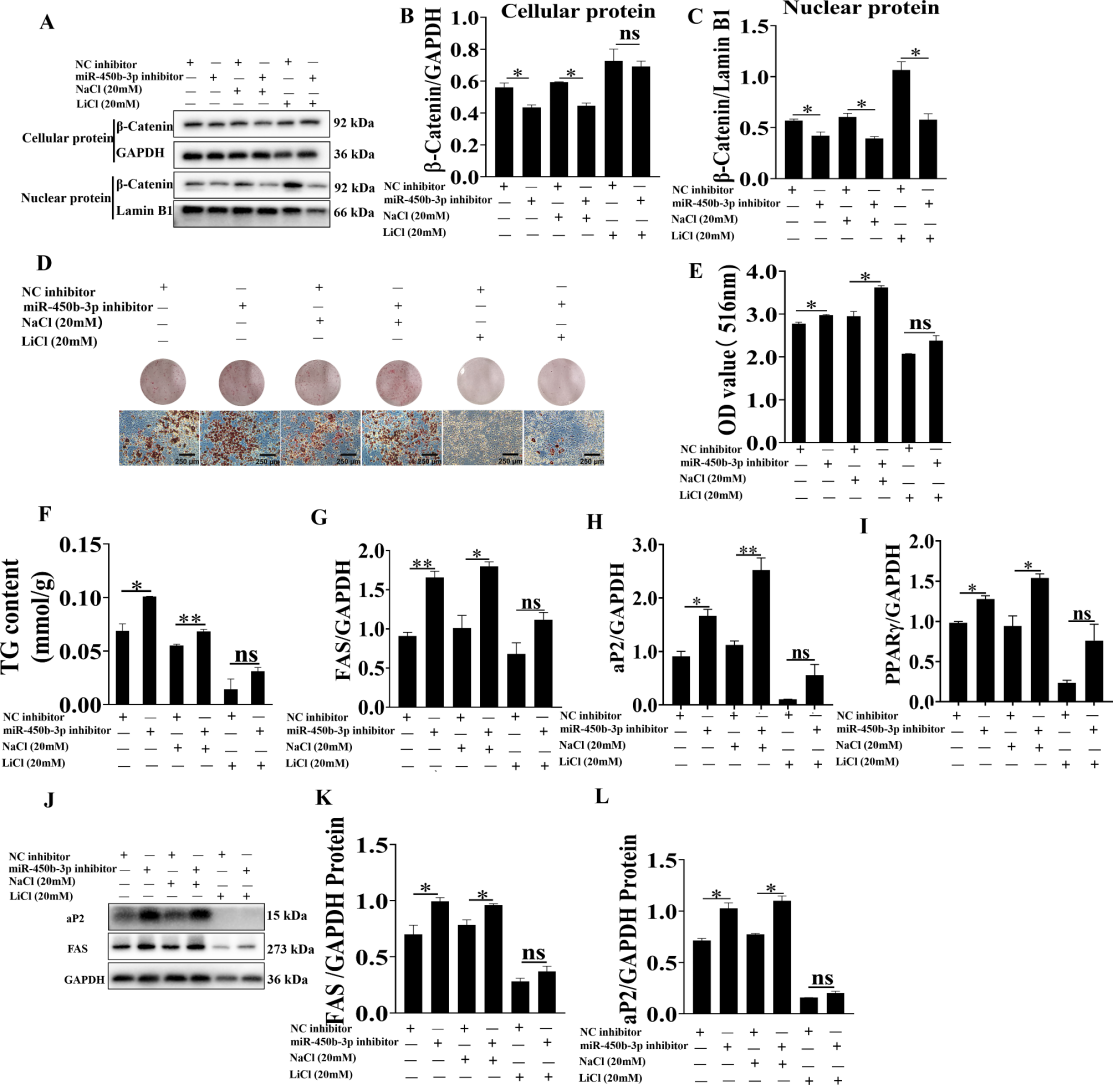
LiCl attenuated the effect of miR-450b-3p mimics. After transfection with miR-450b-3p mimics, 3T3-L1 adipocytes were treated with LiCl. **A-C** The cellular and nuclear protein levels of β-catenin were determined. **D-F** The intracellular lipid content and triglyceride content were examined. **G-I** Real-time qPCR analysis of FAS, aP2, and PPARγ. **J-L** Western blot analysis of the protein expression of the adipogenic markers FAS and aP2. The grouping of blots cropped from different parts of the same gel. The data represent the means ± SEM. n = 3, *P < 0.05, **P < 0.01

Lithium chloride (LiCl), an activator of the Wnt/β-catenin signaling pathway, was used to verify the pathway analysis results of miR-450b-3p. LiCl can suppress the activity of GSK3b, preventing the degradation of β-catenin, which leads to its accumulation and translocation into the nucleus. According to initial experiments, 20 mM LiCl could suppress cell differentiation and expression of β-catenin (Fig. S1). Accordingly, this concentration was selected for further experiments. We treated 3T3-L1 cells with LiCl (with NaCl as negative control) and miR-450b-3p mimic (with NC mimic as control). The downregulation of β-catenin by miR-450b-3p mimic was restored by LiCl (Fig. 6A-C). Oil red O staining showed that the pro-adipogenic effect of miR-450b-3p was attenuated by LiCl (Fig. 6D-F). The upregulation of *FAS*, *aP2* and *PPARγ* by miR-450b-3p was also attenuated by LiCl (Fig. 6G-L). These results demonstrated that the effects of miR-450b-3p on adipogenesis could be attenuated by LiCl as an activator of the Wnt/β-catenin signaling pathway.

Thus, our results indicated that miR-196b-3p and miR-450b-3p affect adipocytes through different signaling pathways.

### **MiR-196b-3p directly targets the 3’UTR of** ***CD47***, **while miR-450b-3p directly targets the 3’UTR of** ***SIRT1***

**Fig. 7:**
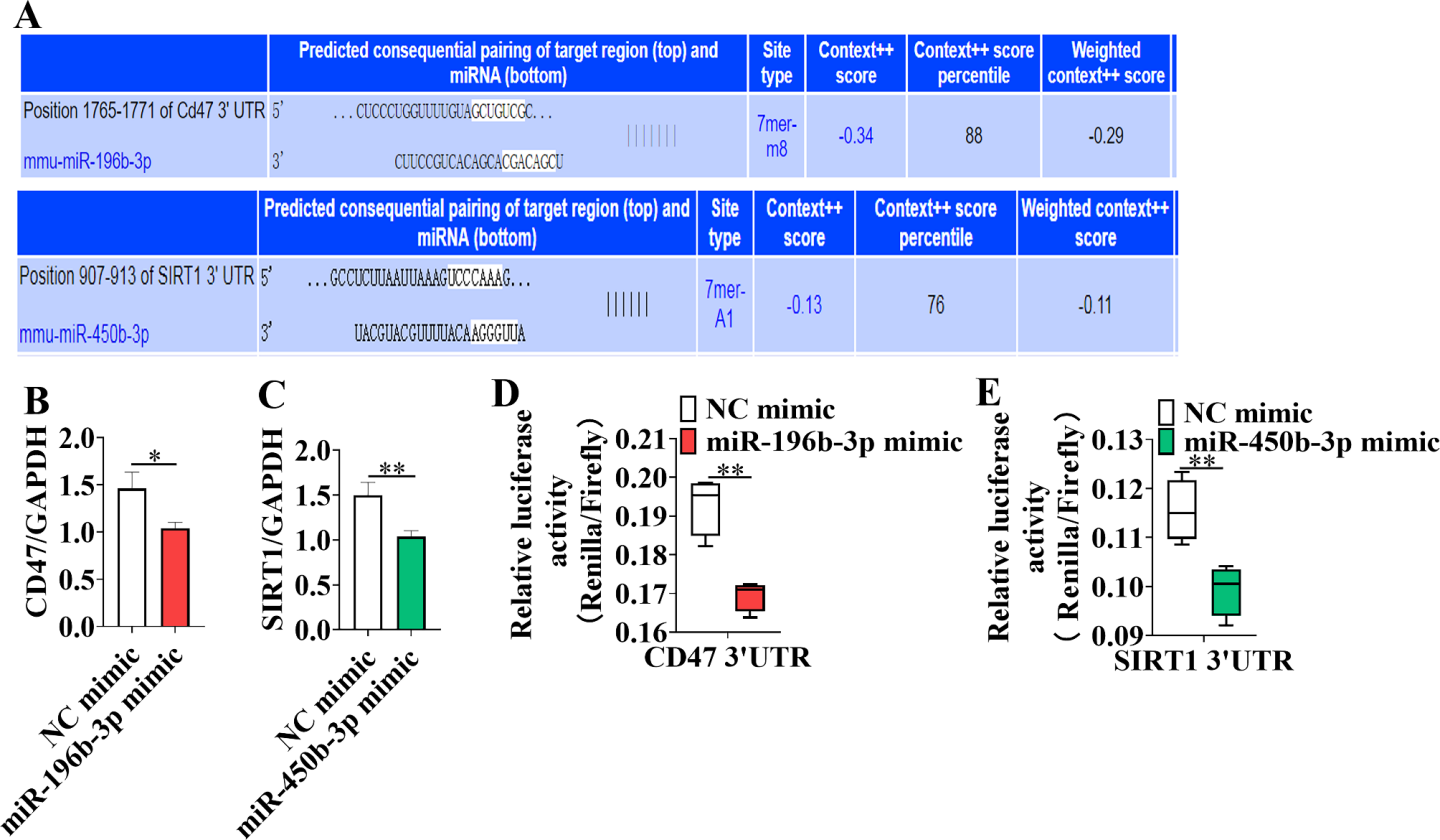
*CD47* is a direct target of miR-196b-3p and SIRT1 is a direct target of miR-450b-3p in adipocytes. A schematic of the respective target sites of miR-196b-3p and miR-450b-3p within the 3’UTR of the CD47 and SIRT1 mRNAs identified using TargetScan (**A**). **B-C** The expression of CD47 and SIRT1 was determined. **D** HEK293T cells were transfected with a luciferase reporter vector containing the miR-196b-3p target sites from the 3’ UTR of CD47 and miR-196b-3p mimics or negative control miRNA. **E** Luciferase reporter analysis of the interaction between SIRT1 and miR-450b-3p. The data represent the means ± SEM n = 3, *P < 0.05, **P < 0.01

To identify the target genes of miR-196b-3p and miR-450b-3p in adipocytes, we used the TargetScan Mouse 7.2 target gene prediction algorithm. Among thousands of predicted targets (Fig. 7A), *CD47* and *SIRT1* attracted our attention due to their essential roles in adipogenesis. Q-PCR analysis confirmed the downregulation of *CD47* and *SIRT1* following transfection with miR-196b-3p or miR-450b-3p mimics (Fig. 7B, C). To investigate if miR-196b-3p directly targets *CD47* and miR-450b-3p directly targets *SIRT1*, we cloned theirs 3’UTR sequences into the psi-CHECK-2 vector next to the *Renilla* luciferase coding sequence. In the dual-luciferase reporter assay, the luciferase activity was significantly reduced in both groups (Fig. 7D&E), which indicated that *CD47* and *SIRT1* are indeed direct target genes of miR-196b-3p and miR-450b-3p, respectively.

### MiR-196b-3p and miR-450b-3p have conserved functions in porcine adipocytes

**Fig. 8:**
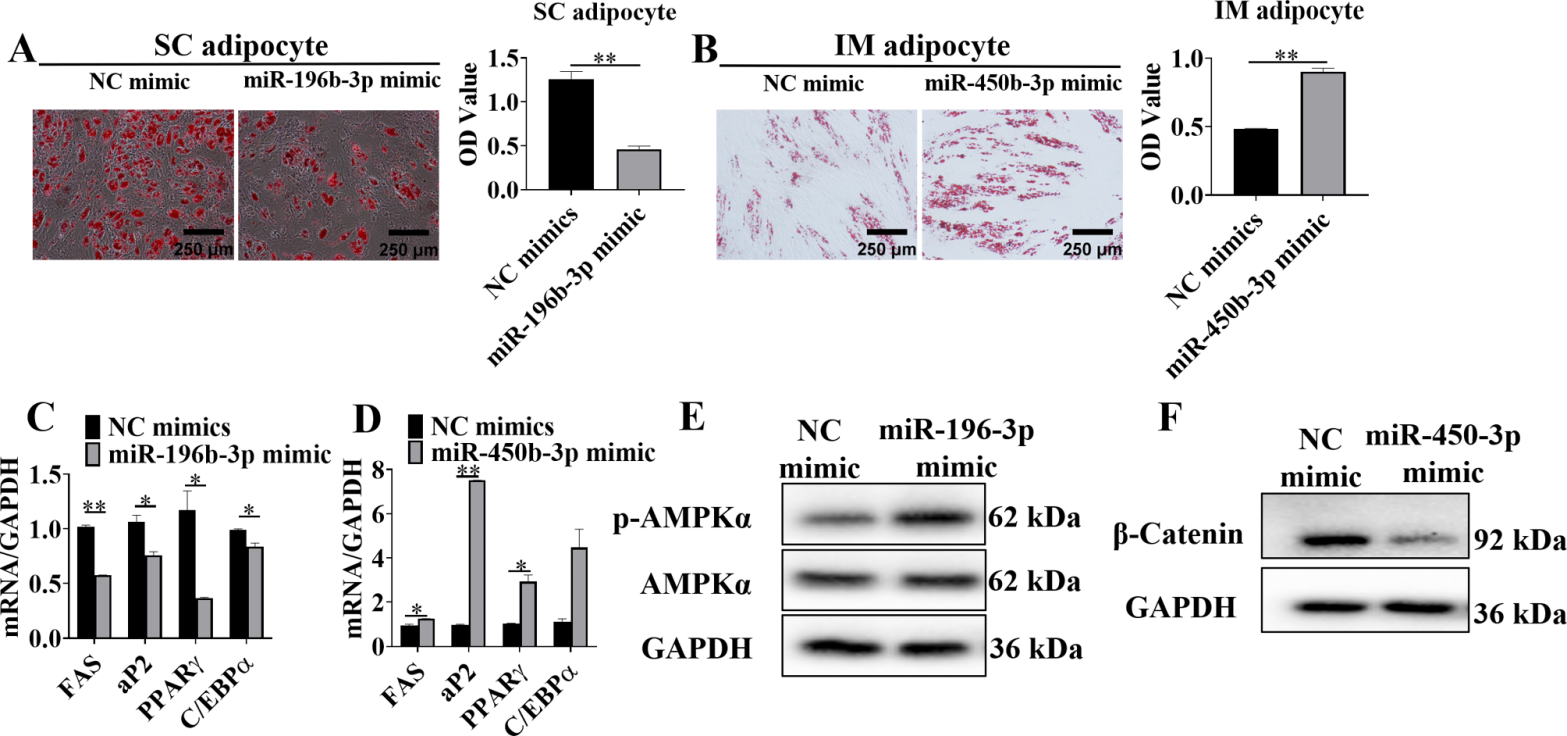
MiR-196b-3p inhibited lipid accumulation via the AMPK pathway in porcine SC adipocytes and miR-450b-3p promoted lipid accumulation via the WNT pathway in porcine IM adipocytes. Before 24 h of induction of differentiation with the cocktail method, porcine SC adipocytes were transfected with miR-196b-3p mimics, and porcine IM adipocytes were transfected with miR-450b-3p mimics. **A-B** SC and IM adipogenesis was detected by oil red O staining. **C-D** Real-time qPCR analysis of the mRNA expression of adipogenic marker genes: *FAS*, *aP2*, *PPARγ*, and *C/EBPα*. **E-F** The protein levels of p-AMPK and β-catenin were determined by western blot analysis. The grouping of blots cropped from different parts of the same gel. The data represent the means ± SEM. n = 3, *P < 0.05, **P < 0.01

Primary cultures of porcine IM and SC adipocytes were used to confirm the results. Since the expression of miR-196-3p was lower in SC than in IM adipocytes, we transfected SC adipocytes with miR-196b-3p mimics and found that the intracellular lipid content decreased after transfection (Fig. 8A). Similarly, the expression of miR-450b-3p was lower in IM than in SC adipocytes, so we transfected IM adipocytes with miR-450b-3p mimics, which led to an increase of the intracellular lipids content (Fig. 8B). Consistently, the expression of adipogenesis and lipolysis marker genes also exhibited corresponding changes (Fig. 8C&D). Furthermore, the activities of the relevant signaling pathways were assessed by western blot analysis. The results revealed that the p-AMPK level increased in IM adipocytes transfected with miR-196b-3p mimics, while the β-catenin level decreased in SC adipocytes transfected with miR-450b-3p mimics (Fig. 8E&F). All these findings suggested that miR-196b-3p and miR-450b-3p have conserved functions in porcine adipocytes.

## Discussion

Pork is one of the most widely consumed meats worldwide and accounts for more than 60% of meat consumption in China [16]. Due to ongoing economic development, consumers are paying increasing attention to the quality of pork. Among the many studied factors affecting pork quality [17, 18], the intramuscular (IM) fat content is considered the most important [1]. However, IM fat deposition is positively correlated with subcutaneous (SC) fat accumulation (r = 0.45) in most pig breeds [19]. Therefore, identifying the differences in the adipogenic regulatory networks of IM and SC adipocytes is the premise for breeding lean pigs with high-quality meat. In this study, a total of 2809 miRNA transcripts were identified from IM and SC adipocytes, 157 of which were differentially expressed between the two kinds of adipocytes. The highest fold changes in IM and SC adipocytes were observed for miR-196b-3p and miR-450b-3p, respectively. Further mechanistic investigations showed that miR-196b-3p inhibited preadipocyte proliferation and adipogenesis through inactivation of the AMPK pathway by targeting *CD47*. Conversely, miR-450b-3p promoted adipogenesis through inactivation of the WNT pathway by targeting *SIRT1*. These results suggest that miR-196b-3p and miR-450b-3p play opposite roles in adipogenesis. Therefore, their different expression levels can partly explain the differences in adipogenic capacity between IM and SC adipocytes.

miRNA exert their biological actions by blocking translation and/or inducing degradation of target mRNAs by seed sequence base-pairing to mRNA recognition sites. In the past decade, an increasing number of studies have indicated that miRNAs such as miR-15a/b [20], miR-127 [21], miR-34a [22, 23], miR-29a/b/c [24, 25], and miR-181a [26], play a significant role in the regulation of porcine adipogenesis. However, few studies have explained the different roles of miRNAs in regulating the adipogenic capacity of IM and SC adipocytes. Here, we found that miR-196b-3p expression was 32-fold higher in IM adipocytes than in SC adipocytes. Functionally, miR-196b-3p inhibited adipogenesis by targeting *CD47*, a transmembrane cell receptor previously implicated in self-recognition and immune cell infiltration [27, 28]. In contrast to WT mice, *CD47* knockout mice exhibited anti-diet induced obesity and a reduced fat/body weight ratio [29]. In addition, *CD47* knockout alleviated cardiac remodeling, probably by improving AMPK signaling, which suggested that *CD47* is an upstream regulator of the AMPK signaling pathway [30]. AMPK is a highly conserved heterotrimeric enzyme composed of a catalytic (α) and two regulatory (β and γ) subunits. AMPK is a metabolic energy sensor that plays a key role in regulating energy homeostasis [31, 32]. Phosphorylated AMPK has been reported to phosphorylate HSL, and p-HSL is necessary for lipolysis [33]. AMPK has also been proved to be an anti-adipogenic factor, which suppresses the proliferation and differentiation of adipocytes. This effect was accompanied by a significant reduction in the expression of *PPARγ*, *FAS*, and *aP2* in 3T3-L1 adipocytes [12, 32–35]. In the present study, we found that miR-196b-3p overexpression increased p-AMPK and p-HSL levels, while miR-196b-3p inhibitor decreased p-AMPK and p-HSL levels in the context of adipogenesis. We further found that the AMPK signaling pathway agonist AICAR [33] attenuated the effects of miR-196b-3p inhibitor on adipogenesis.

We also found that the expression of miR-450b-3p was approximately 8-fold higher in SC than in IM adipocytes. MiR-450b-3p promotes adipogenesis by targeting *SIRT1*, which is known to deacetylate histones and non-histone proteins including transcription factors, thereby regulating metabolism, stress resistance, cellular survival, cellular senescence/aging, inflammation-immune function, endothelial function, and the circadian rhythm [36]. *SIRT1* was identified as a regulator of diverse biological processes, including adipogenesis and lipolysis. *SIRT1* represses the adipose-specific nuclear hormone receptor *PPARγ* by interacting with its cofactors, nuclear receptor co-repressor and silencing mediator of retinoid and thyroid hormone receptors (SMRT), thereby reducing adipogenesis and promoting lipolysis [37]. In the present study, we found that the expression levels of miR-450b-3p and *SIRT1* were negatively correlated, while the dual-luciferase assay also confirmed their binding, which proved that *SIRT1* is the target gene of miR-450b-3p. Notably, *SIRT1* was previously reported to inhibit adipocyte differentiation by activating Wnt/β-catenin signaling [38]. In the canonical Wnt signaling pathway, β-catenin plays a central role as a transcriptional coactivator. When β-catenin enters the nucleus, it turns on transcription by binding to members of the TCF/LEF family of transcription factors. A critical step in the activation of Wnt/β-catenin signaling is the formation of a complex comprising β-catenin and members of the TCF/LEF family. Previous studies have shown that activation of Wnt/β-catenin signaling inhibits lipogenesis by preventing C/EBPα-mediated PPARγ activation. *SIRT1* deacetylates β-catenin, causing its accumulation in the nucleus of mesenchymal stem cells, thus increasing osteogenic differentiation and decreasing adipogenic differentiation [39]. Another study found that resveratrol, an activator of *SIRT1*, increased the protein expression levels of β-catenin in porcine pancreatic stem cells [40]. In this study, we used western blot analysis to determine the protein content of β-catenin in the nucleus. The results showed that overexpression of miR-450b-3p significantly reduced the nuclear translocation of β-catenin by targeting *SIRT1*. We then treated adipocytes with LiCl [41], an activator of WNT signaling pathway, and confirmed that the effects of miR-450b-3p on adipogenesis were weakened by LiCl. These results suggest that miR-450b-3p promotes adipogenic differentiation by targeting *SIRT1* to decrease the translocation of β-catenin to the nucleus.

## Conclusions

In conclusion, we clarified the importance of miR-196b-3p and miR-450b-3p in adipogenic differentiation, revealing that miR-196b-3p inhibits adipogenesis via the AMPK signaling pathway by targeting *CD47*, while miR-450b-3p promotes adipogenesis via the WNT/β-catenin signaling pathway by targeting *SIRT1*. These findings not only help us understand the mechanisms through which miR-196b-3p and miR-450b-3p affect adipogenesis, but also provide novel molecular targets for controlling adipogenesis to improve the quality of pork.

## Materials and methods

### Animals

Three 3-day-old Jiaxing black piglets were provided by Zhejiang Qinglian Food Co., Ltd (Jiaxing, Zhejiang Province, China). Longissimus thoracis muscle for the isolation of IM adipocytes and subcutaneous adipose tissues for the isolation of SC adipocytes were collected from the piglets after they were euthanized with isoflurane. All animal experiments were performed following the guidelines of the China Animal Protection Association and the Jiaxing University Animal Care Committee.

### Adipocyte isolation, differentiation and RNA-seq

Isolated tissues were cut into small pieces of approximately 1 mm^3^ and digested with 1 mg/mL collagenase type I (Invitrogen, Carlsbad, CA, USA) in a 37 °C constant temperature shaking water bath for 60 min. The digested tissues were passed through a 70 μm pore-size nylon mesh, and centrifuged at 1500 × g for 10 min to obtain adipose-derived stromal-vascular (SV) cells. These cell suspensions are cultured in DMEM/F12 medium (HyClone, USA) containing 1% antibiotic/antimycotic solution (SV30010; HyClone, USA) and 10% fetal bovine serum (FBS; Gibco, USB) at 37 °C in a humidified atmosphere comprising 5% CO_2_. After cell confluence, the SV cells were induced with a differentiation cocktail comprising DMEM/F12 supplemented with 10% FBS, 0.5 mM isobutyl methylxanthine (IBMX; Sigma, USA), 0.5 mM dexamethasone (Sigma, USA), and 20 nM insulin (Sigma, USA) for 2 days. After induction, the cells were maintained in DMEM/F12 with 10% FBS and 20 nM insulin for another 4–6 days. On day 8 after adipogenic induction, the cultured IM and SC adipocytes were harvested and sequenced on a BGISEQ-500 platform (BGI Hangzhou, China).

### RNA extraction & miRNA library constuction

Total RNA was extracted from the tissues using Trizol (Invitrogen, Carlsbad, CA, USA) according to the manual instructions. The mix was centrifuge at 12,000×g for 5 min at 4 °C. The supernatant was transferred to a new 2.0ml tube which was added 0.3ml of Chloroform/isoamyl alcohol (24:1) per 1.5ml of Trizol reagent. After the mix was centrifuged at 12,000×g for 10 min at 4 °C, the aqueous phase was transferred to a new 1.5mL tube which was add equal volume of supernatant of isopropyl alcohol. The mix was centrifuged at 12,000×g for 20 min at 4 °C and then removed the supernatant. After washed with 1ml 75% ethanol, the RNA pellet was air-dried in the biosafety cabinet and then dissolved by add 25µL ~ 100µL of DEPC-treated water. Subsequently, total RNA was qualified and quantified using a Nano Drop and Agilent 2100 bioanalyzer (Thermo Fisher Scientific, MA, USA).

Library was prepared with 1 µg total RNA for each sample. Total RNA was purified by electrophoretic separation on a 15% urea denaturing polyacrylamide gel electrophoresis (PAGE) gel and small RNA regions corresponding to the 18–30 nt bands in the marker lane (14–30 ssRNA Ladder Marker, TAKARA) were excised and recovered. Then the 18–30 nt small RNAs were ligated to a 5’-adaptor and a 3’-adaptor. The adapter-ligated small RNAs were subsequently transcribed into cDNA by SuperScript II Reverse Transcriptase (Invitrogen, USA) and then several rounds of PCR amplification with PCR Primer Cocktail and PCR Mix were performed to enrich the cDNA fragments. The PCR products were selected by agarose gel electrophoresis with target fragments 100 ~ 120 bp, and then purified by QIAquick Gel Extraction Kit (QIAGEN, Valencia, CA). The library was quality and quantitated in two methods: check the distribution of the fragments size using the Agilent 2100 bioanalyzer, and quantify the library using real-time quantitative PCR (QPCR) (TaqMan Probe). The final ligation PCR products were sequenced using the BGISEQ-500 platform (BGI-Shenzhen, China).

### Data processing and analysis

FastQC (http://www.bioinformatics.babraham.ac.uk/projects/fastqc/) and Fastx (fastx_toolkit-0.0.13.2) are used for quality control and pre-processing of sequencing data. The reads were annotated to the pig genome using mirDeep2.0.1.2 [42] software and the miRBase 20.0 database to identify known conserved miRNAs and predict novel miRNAs. Statistical analysis of the data using the DESeq (http://bioinfo.au.tsinghua.edu.cn/software/degseq/) [43] software based on the negative binomial distribution (http://www.bioconductor.org/packages/release/bioc/html/edgeR.html/) was used to obtain differentially expressed miRNAs. RNAhybrid and miRanda were used for microRNA target gene prediction. DAVID software (http://david.abcc.ncifcrf.gov/) was used for GO and KEGG pathway analysis [15].

### Quantitative real-time PCR

Real-time quantitative PCR (SYBRGreen dye method) was used for quantitative detection. The reaction system included nuclease-Free Water (3.4 µL), 10 µM upstream primer (0.3 µL), 10 µM downstream primer (0.3 µL), SYBR mix (5 µL), and 1 µL sample in a total volume of 10 µL. The PCR temperature program included denaturation at 95 ° C for 2 min, followed by 40 cycles of 95℃ for 2 s, 60℃ for 20 s, and 70℃ for 10 s. The Ct value was automatically generated using the default settings, with three technical replicates per sample. U6, β-actin and GAPDH were used as the reference genes of QRT-PCR. The primer sequences are listed in Supplementary Table 2. The primer of miRNA was synthesized by Guangzhou RiboBio Co., Ltd.

### Western blot analysis

RIPA buffer (Beyotime, Shanghai, China) supplemented with protease inhibitor (Pierce, Bradenton, Florida, USA) was used to extract the total protein. The lysates were centrifuged at 1360 g for 7 min, and the supernatant was boiled in sodium dodecyl sulfate (SDS) loading buffer (Beyotime, Shanghai, China) for 10 min. After separation on 12% acrylamide SDS-PAGE gels, the protein bands were transferred onto a polyvinylidene difluoride membrane (CST, Danvers, Massachusetts, USA). The membrane was then blocked in 5% defatted milk. To expose more different proteins, we cut the whole membrane before hybridizing the antibodies, and then incubated the different primary antibodies (Abcam, Cambridge, UK. 1:1000) at 4 °C overnight. After cleaning, the membranes were incubated with horseradish peroxidase-conjugated secondary antibodies (Abcam, Cambridge, UK. 1:20000). Protein bands were visualized using chemiluminescence reagent (Millipore, Massachusetts, USA) and analyzed using Quantity One 4.6.3 Image software, as described previously [15].

### Transfection of adipocytes with miRNA mimics/inhibitor

The miRNA-NC, mimics(20 µM), inhibitor (20 µM) was incubated with lipofectamin 2000 in Opti-MEM for 20 min, after which the mixture was added to cultured cells. After 6 h, the medium was replaced by the culture medium.

### EdU staining and CCK-8 assay

EdU staining and the cell count kit 8 (CCK-8) assay were performed as described previously [15]. Adipocytes were transfected with miRNA-NC, inhibitor or mimics at 40% confluence, and the proliferation related assays were performed 48 h after transfection.

### Oil Red O staining

The cells treated with miRNA-NC, inhibitor or mimics were allowed to mature for 8 days, and then washed with PBS, fixed with 4% paraformaldehyde for 30 min at room temperature, and washed again three times with PBS. A mixture of Oil Red O stock solution (0.6% Oil Red O dye in isopropanol) and water at a 6:4 ratio was dripped onto the cells and allowed to stain for 30 min, followed by washing four times with PBS. Images were captured under a conventional optical microscope (Nikon, Tokyo, Japan).

### Triglyceride content assay

On day 8 of differentiation after transfection with miRNA-inhibitor or mimics, the intracellular triglyceride content was measured using a commercial triglyceride assay kit (Nan Jing Jian Cheng Bioengineering Institute, China) according to the manufacturer’s instructions.

### Luciferase reporter assay

CD47 3’-UTR was subcloned into the psiCHECK-2™construct (Promega), and the resulting reporter constructs were used to transfect 293T cells along with either miR-196b-3p mimic or control. Lipofectamine 3000 reagent was used for transfection. SIRT1 3’-UTR psiCHECK-2™ vector and miR-450b-3p mimic were used to co-transfect 293T cells. At 48 h post-transfection, the Dual Luciferase Reporter Assay System was used to analyze the luciferase activity as described before [15].

### Statistical analysis

All data were derived from at least three independent experiments and presented as means ± SEM. Differences between groups were analyzed using Student’s two-tailed *t*-test when only two groups were compared, or using single-factor analysis of variance (one-way ANOVA) when more than two groups were compared. Differences with *P*-values < 0.05 were considered statistically significant.

## Electronic supplementary material

Below is the link to the electronic supplementary material.

Supplementary Material 1

Supplementary Material 2

Supplementary Material 3

## Data Availability

The data sets supporting the results of this article are included within the manuscript and its additional files. The RNA-Seq data were submitted to the NCBI database and were given the GenBank accession numbers MN752885-MN753975. The raw datasets are available from the corresponding author on reasonable request.

## Abbreviations

IM: intramuscular
SC: subcutaneous
FAS: fatty acid synthase
aP2: fatty acid binding protein 4
PPARγ: peroxisome proliferator-activated receptorγ
C/EBPs: CCAAT enhancer binding proteins
AMPK: AMP-activated protein kinase
ACC: acetyl-CoA carboxylase
SIRT1: Sirtuin 1
NC: negative control
CDK6: cyclin dependent kinase 6
CCK-8: cell counting assay 8
ATGL: adipose triglyceride lipase
HSL: hormone-sensitive lipase
