# Supplementary material for: MiR-196b-3p and miR-450b-3p are key regulators of adipogenesis in porcine intramuscular and subcutaneous adipocytes
Source: BMC Genomics. 2023 Jun 27;24:360. doi: 10.1186/s12864-023-09477-0 (PMC10303896; doi:10.1186/s12864-023-09477-0)
Supplement: Supplementary file 1 — Supplementary Material 1 [file 12864_2023_9477_MOESM1_ESM.pdf]

**Supplementary Table 1 The number of non-coding small RNAs detected in each sample**

| Sample name | miRNA count | piRNA count | siRNA Count |
|---|---|---|---|
| SC1 | 475 | 295 | 0 |
| SC2 | 454 | 282 | 0 |
| SC3 | 473 | 325 | 0 |
| IM1 | 487 | 300 | 0 |
| IM2 | 459 | 265 | 0 |
| IM3 | 457 | 265 | 0 |

**Supplementary Table 2 Primers (S, sense; A, antisense) for real time PCR.**

| Gene | Accession Number | Primer Sequences | Production length /bp | Tm/°C |
|---|---|---|---|---|
| FAS | DQ846748.1 | S: AGTTGCCCGAGTCAGAGAA<br>A: CGTCGAACTTGGAGAGATCC | 150 | 60 |
| FABP4 | NM_024406.3 | S: AAGAAGTGGGAGTGGGCTTTG<br>A: CTCTTCACCTTCCTGTCGTCTG | 184 | 60 |
| C/EBPα | NM_001287514.1 | S: TGGACAAGAACAGCAACGAG<br>A: TCACTGGTCAACTCCAGCAC | 127 | 60 |
| PPARγ | NM_001287514.1 | S: CCAAGAATACCAAAGTGCGATCA<br>A: CCCACAGACTCGGCACTCAAT | 133 | 60 |
| ATGL | NM_001163689.1 | S: TTCGCAATCTCTACCGCCTC<br>A: AAAGGGTTGGGTTGGTTCAG | 136 | 60 |
| HSL | NM_010719.5 | S: GCTGGGCTGTCAAGCACTGT<br>A: GTAACTGGGTAGGCTGCCAT | 160 | 60 |
| Cyclin B | X58708.1 | S: AACTTCAGCCTGGGTCG<br>A: CAGGGAGTCTTCACTGTAGGA | 136 | 60 |
| Cyclin D | NM_001379248.1 | S: TAGGCCCTCAGCCTCACTC<br>A: CCACCCCTGGGATAAAGCAC | 160 | 60 |
| Cyclin E | NM_001287514.1 | S: CAGAGCAGCGAGCAGGAGC<br>A: GCAGCTGCTTCCACACCACT | 127 | 60 |
| CDK6 | NM_001287514.1 | S: TGGATAAAGTTCCAGAGCCCG<br>A: TTCTGCGGTTTCAGATCACGA | 133 | 60 |
| β-actin | NM_007393.5 | S: GTCCCTGACCCTCCCAAAAG<br>A: GCTGCCTCAACACCTCAACCC | 266 | 60 |
| GAPDH | NM_007393.5 | S: TGCTGAGTATGTCGTGGAGTCT<br>A: ATGCATTGCTGACAATCTTGAG | 138 | 60 |
