# Supplementary material for: MiR-196b-3p and miR-450b-3p are key regulators of adipogenesis in porcine intramuscular and subcutaneous adipocytes
Source: BMC Genomics. 2023 Jun 27;24:360. doi: 10.1186/s12864-023-09477-0 (PMC10303896; doi:10.1186/s12864-023-09477-0)
Supplement: Supplementary file 2 — Supplementary Material 2 [file 12864_2023_9477_MOESM2_ESM.pdf]

Figure 2

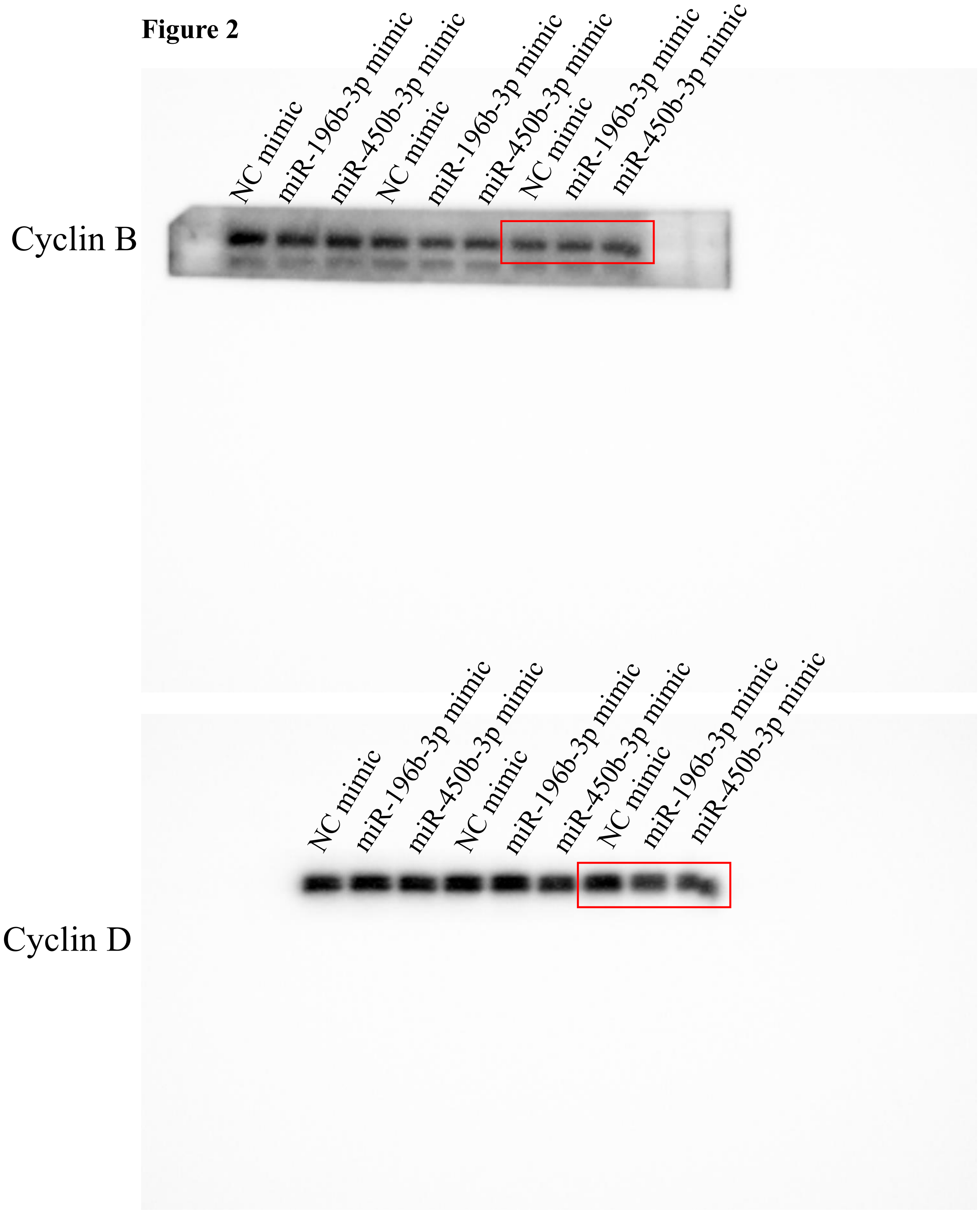

NC mimic
miR-196b-3p mimic
miR-450b-3p mimic
NC mimic
miR-196b-3p mimic
miR-450b-3p mimic
NC mimic
miR-196b-3p mimic
miR-450b-3p mimic
Cyclin B
NC mimic
miR-196b-3p mimic
miR-450b-3p mimic
NC mimic
miR-196b-3p mimic
miR-450b-3p mimic
NC mimic
miR-196b-3p mimic
miR-450b-3p mimic
Cyclin D

GAPDH

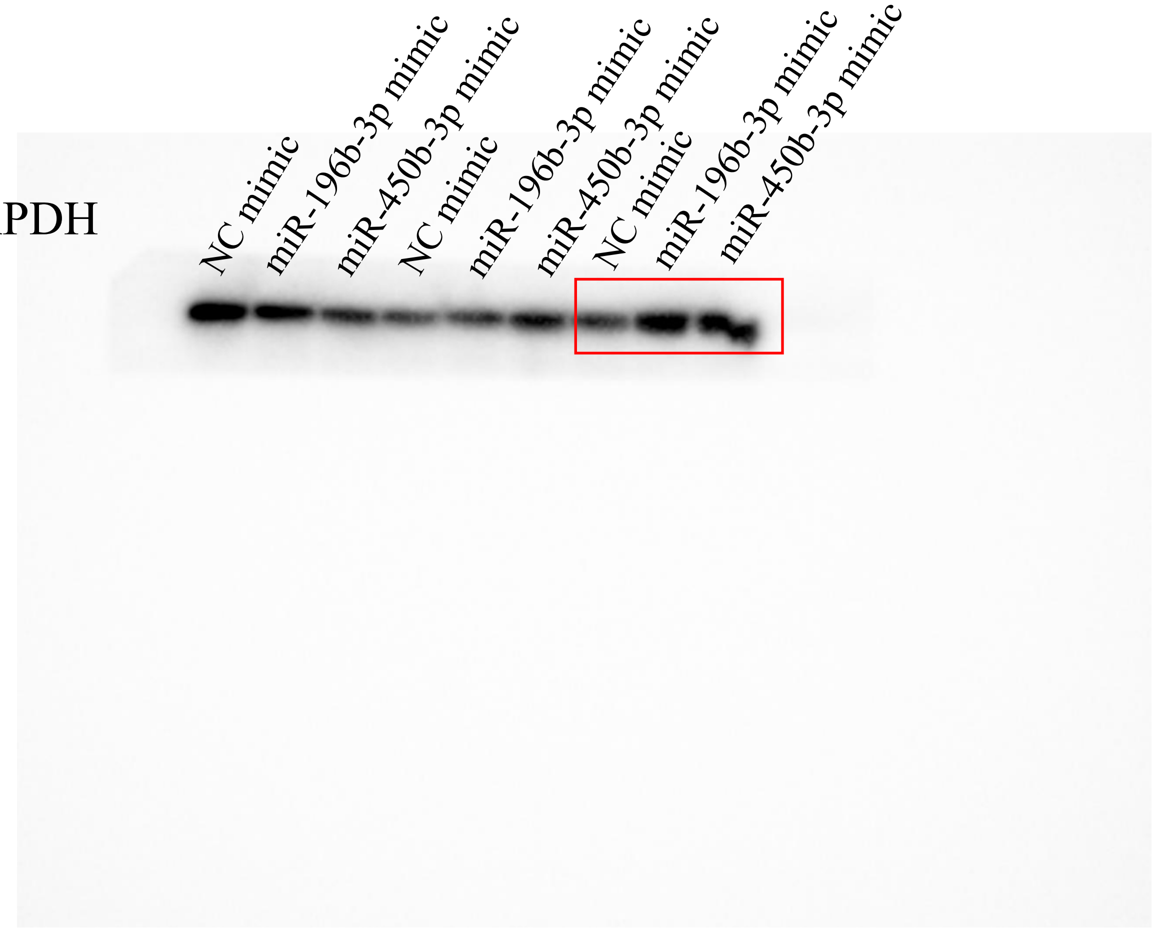

Cyclin B

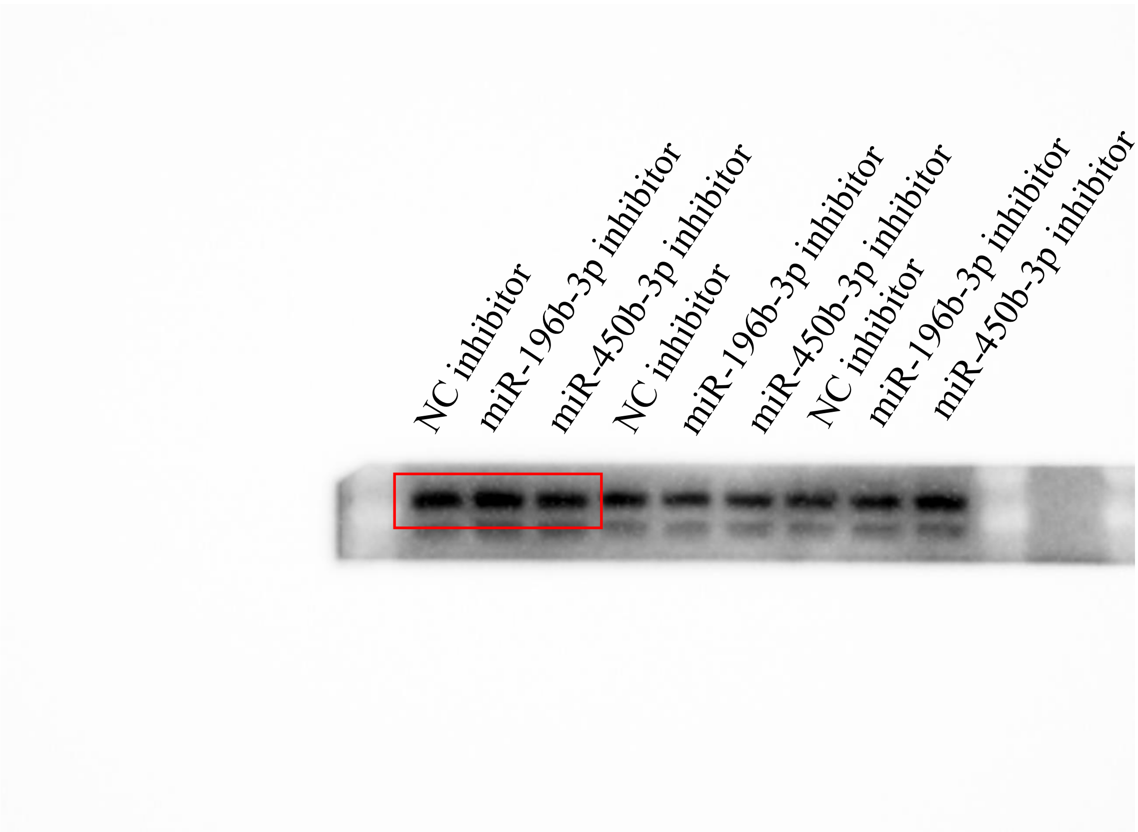

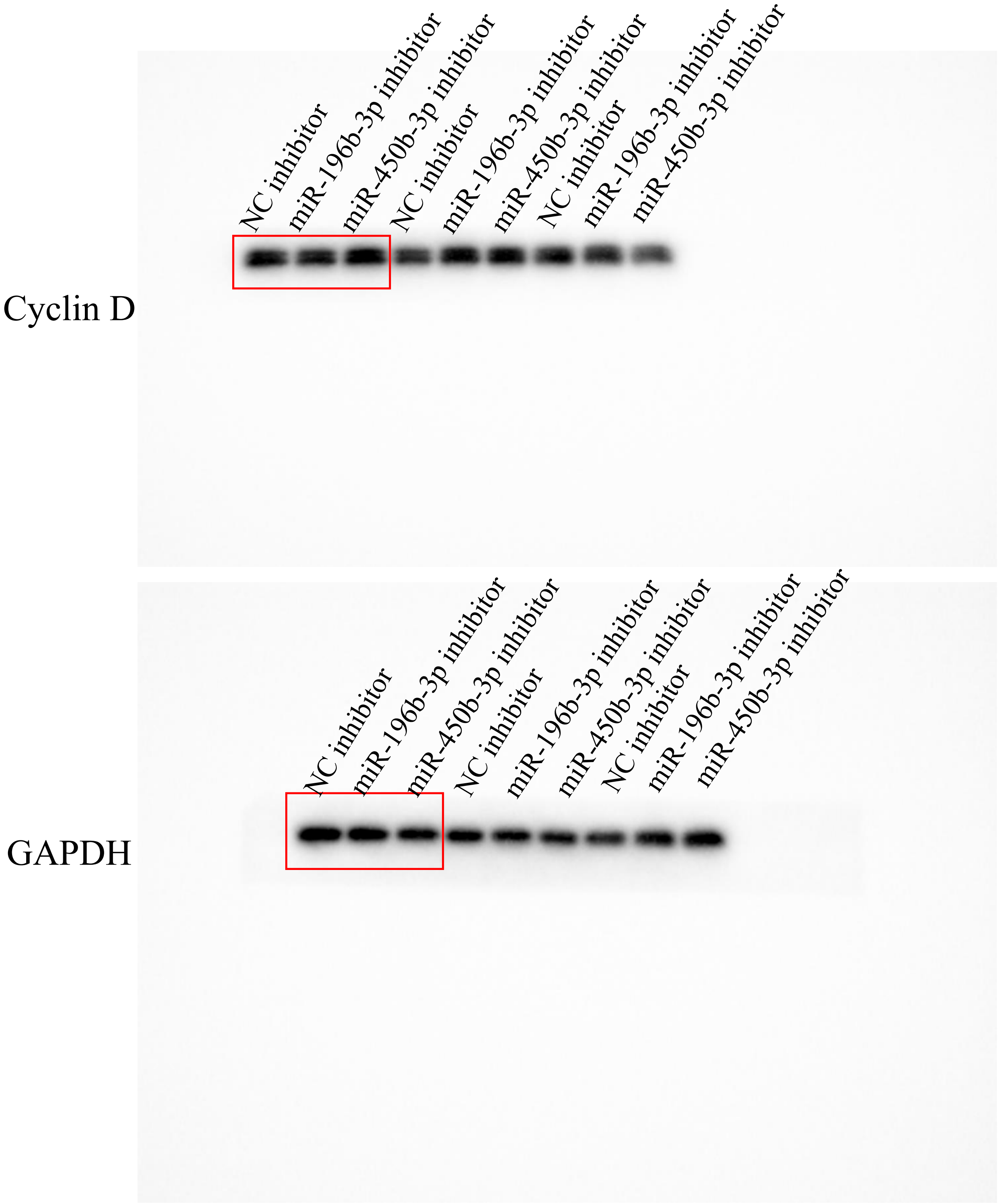
NC inhibitor
miR-196b-3p inhibitor
miR-450b-3p inhibitor
NC inhibitor
miR-196b-3p inhibitor
miR-450b-3p inhibitor
NC inhibitor
miR-196b-3p inhibitor
miR-450b-3p inhibitor
Cyclin D
NC inhibitor
miR-196b-3p inhibitor
miR-450b-3p inhibitor
NC inhibitor
miR-196b-3p inhibitor
miR-450b-3p inhibitor
NC inhibitor
miR-196b-3p inhibitor
miR-450b-3p inhibitor
GAPDH

Figure 3

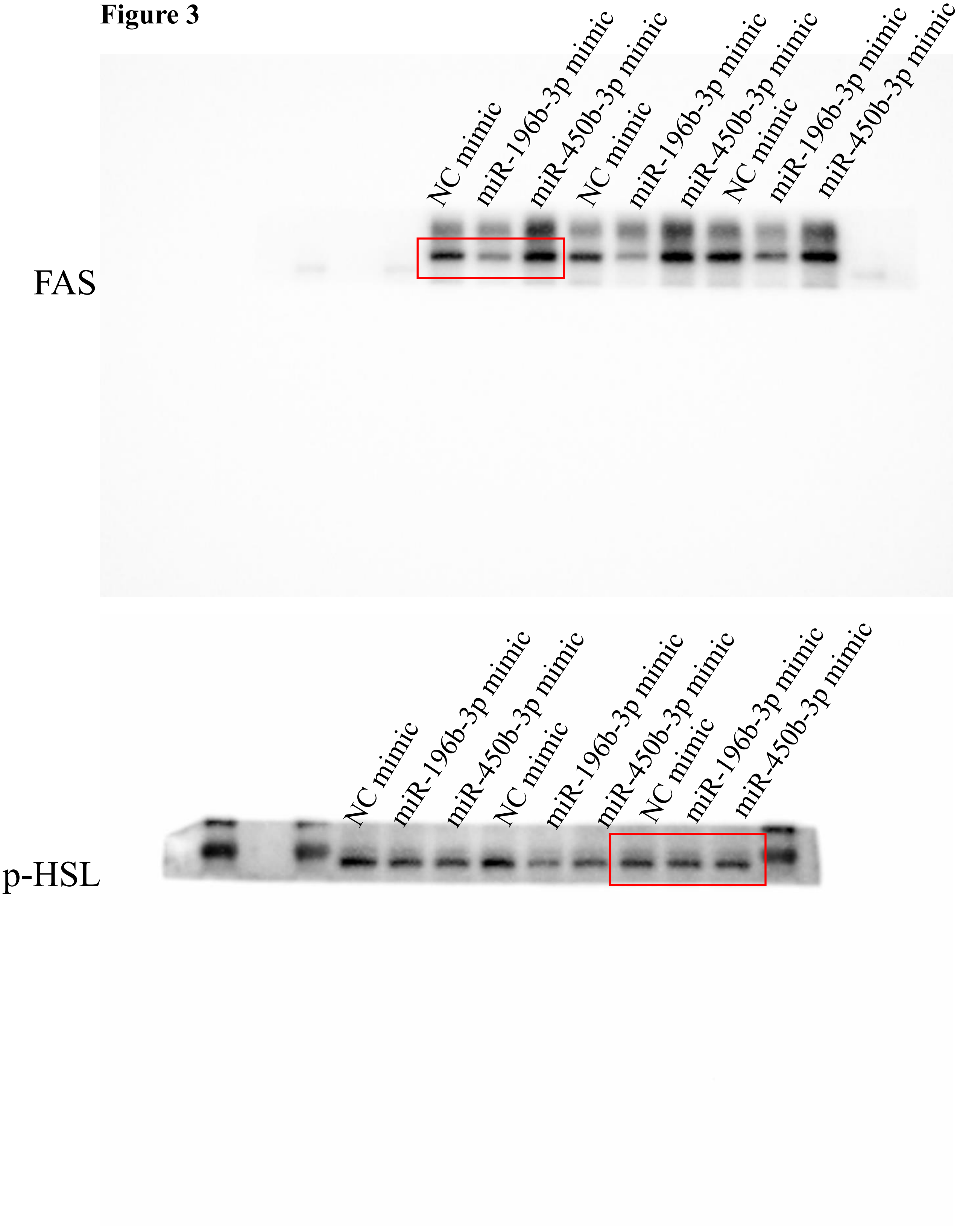
FAS
NC mimic
miR-196b-3p mimic
miR-450b-3p mimic
NC mimic
miR-196b-3p mimic
miR-450b-3p mimic
NC mimic
miR-196b-3p mimic
miR-450b-3p mimic
p-HSL
NC mimic
miR-196b-3p mimic
miR-450b-3p mimic
NC mimic
miR-196b-3p mimic
miR-450b-3p mimic
NC mimic
miR-196b-3p mimic
miR-450b-3p mimic

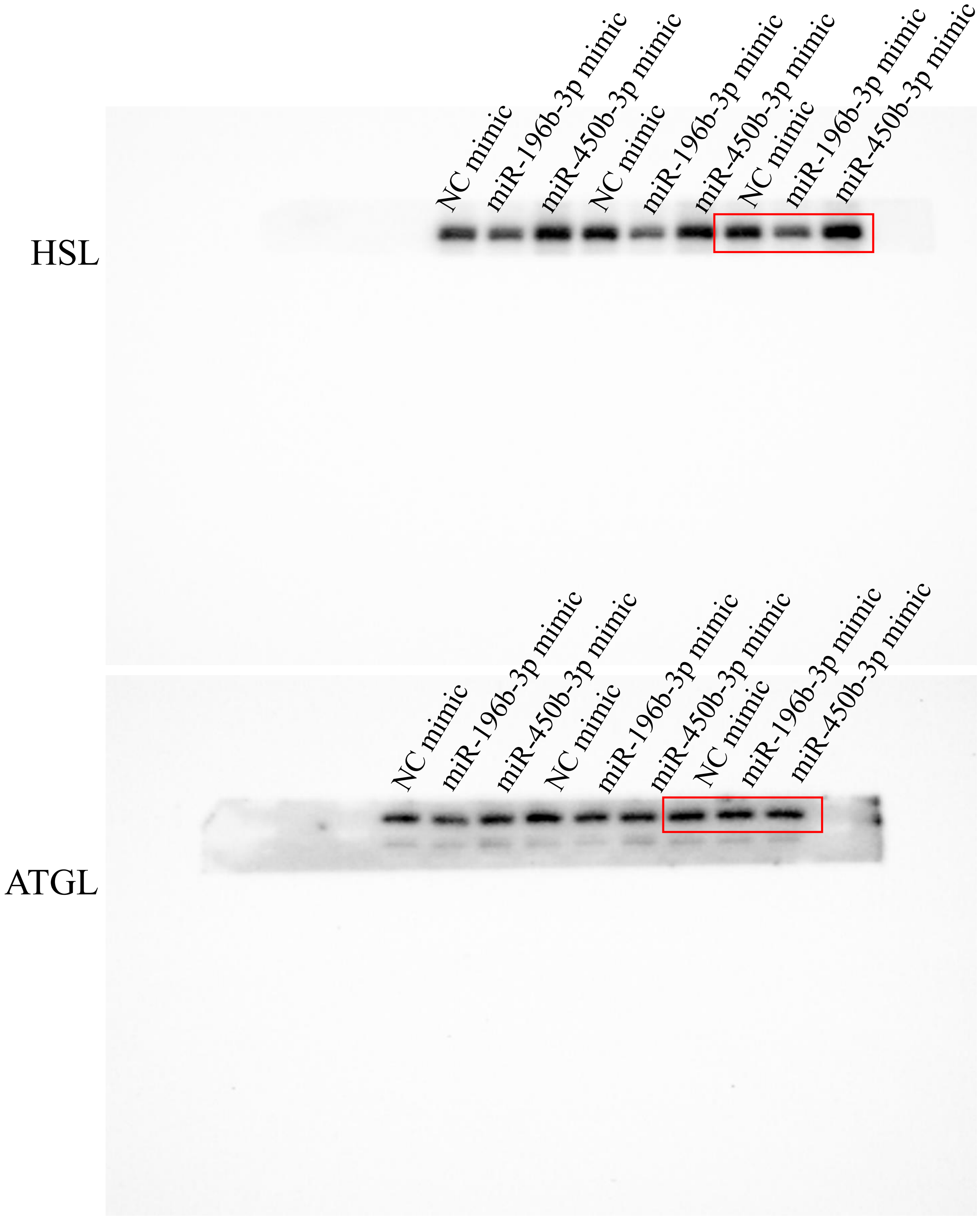
HSL
NC mimic
miR-196b-3p mimic
miR-450b-3p mimic
NC mimic
miR-196b-3p mimic
miR-450b-3p mimic
NC mimic
miR-196b-3p mimic
miR-450b-3p mimic
ATGL
NC mimic
miR-196b-3p mimic
miR-450b-3p mimic
NC mimic
miR-196b-3p mimic
miR-450b-3p mimic
NC mimic
miR-196b-3p mimic
miR-450b-3p mimic

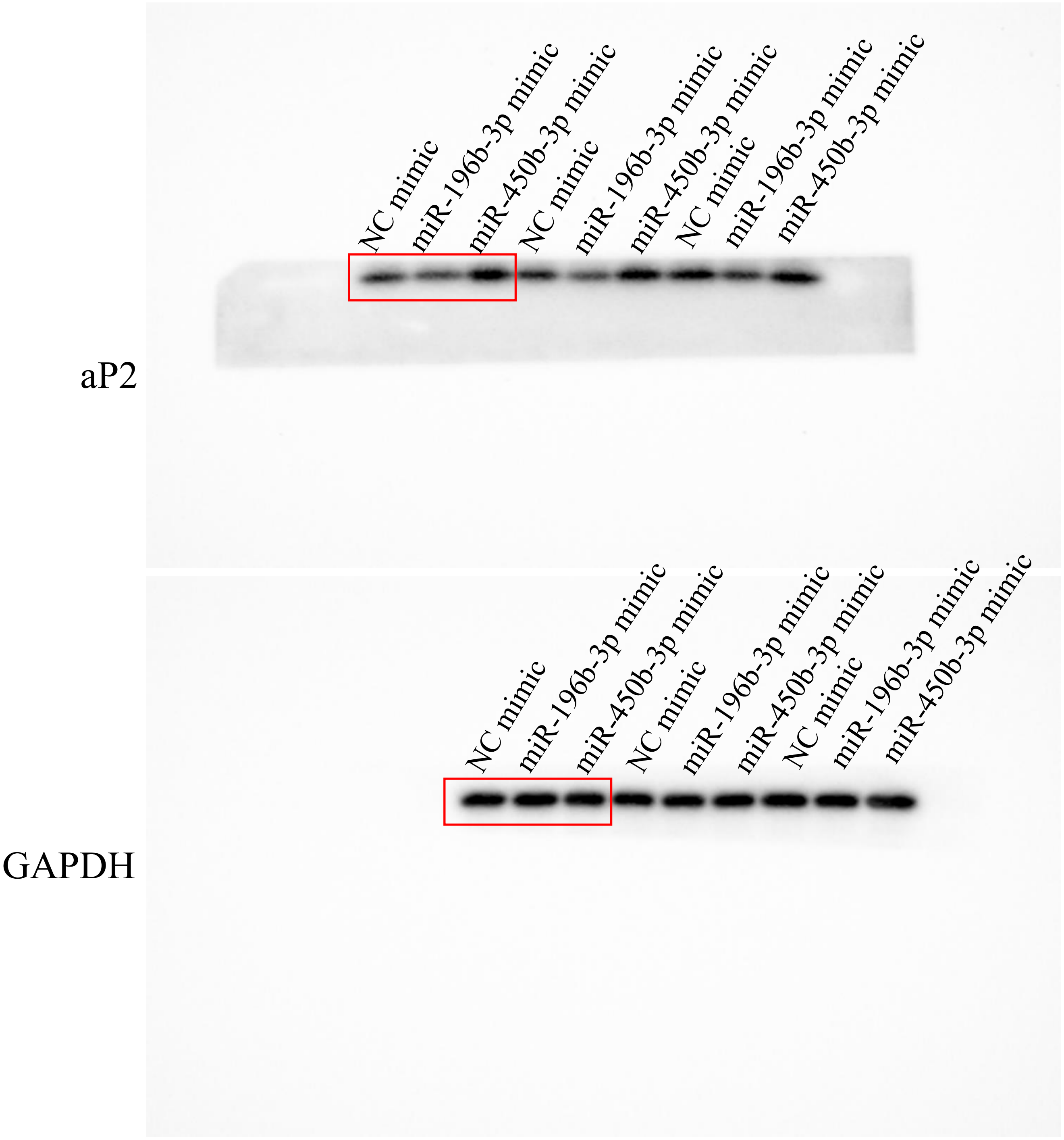
NC mimic
miR-196b-3p mimic
miR-450b-3p mimic
NC mimic
miR-196b-3p mimic
miR-450b-3p mimic
NC mimic
miR-196b-3p mimic
miR-450b-3p mimic
aP2
NC mimic
miR-196b-3p mimic
miR-450b-3p mimic
NC mimic
miR-196b-3p mimic
miR-450b-3p mimic
NC mimic
miR-196b-3p mimic
miR-450b-3p mimic
GAPDH

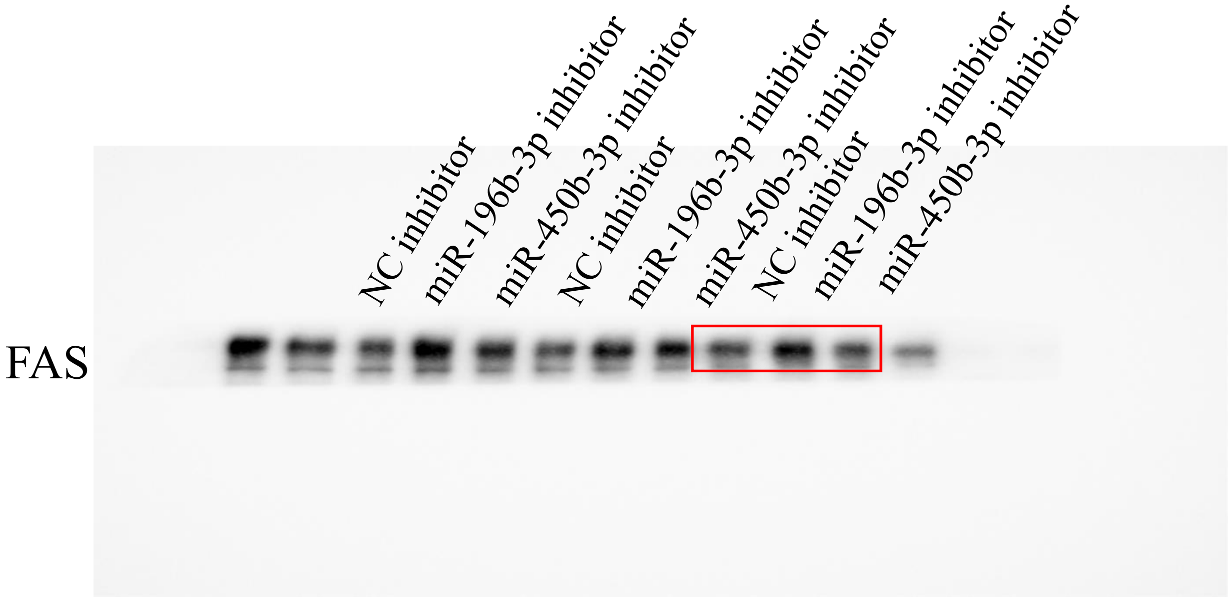
NC inhibitor
miR-196b-3p inhibitor
miR-450b-3p inhibitor
NC inhibitor
miR-196b-3p inhibitor
miR-450b-3p inhibitor
NC inhibitor
miR-196b-3p inhibitor
miR-450b-3p inhibitor
FAS

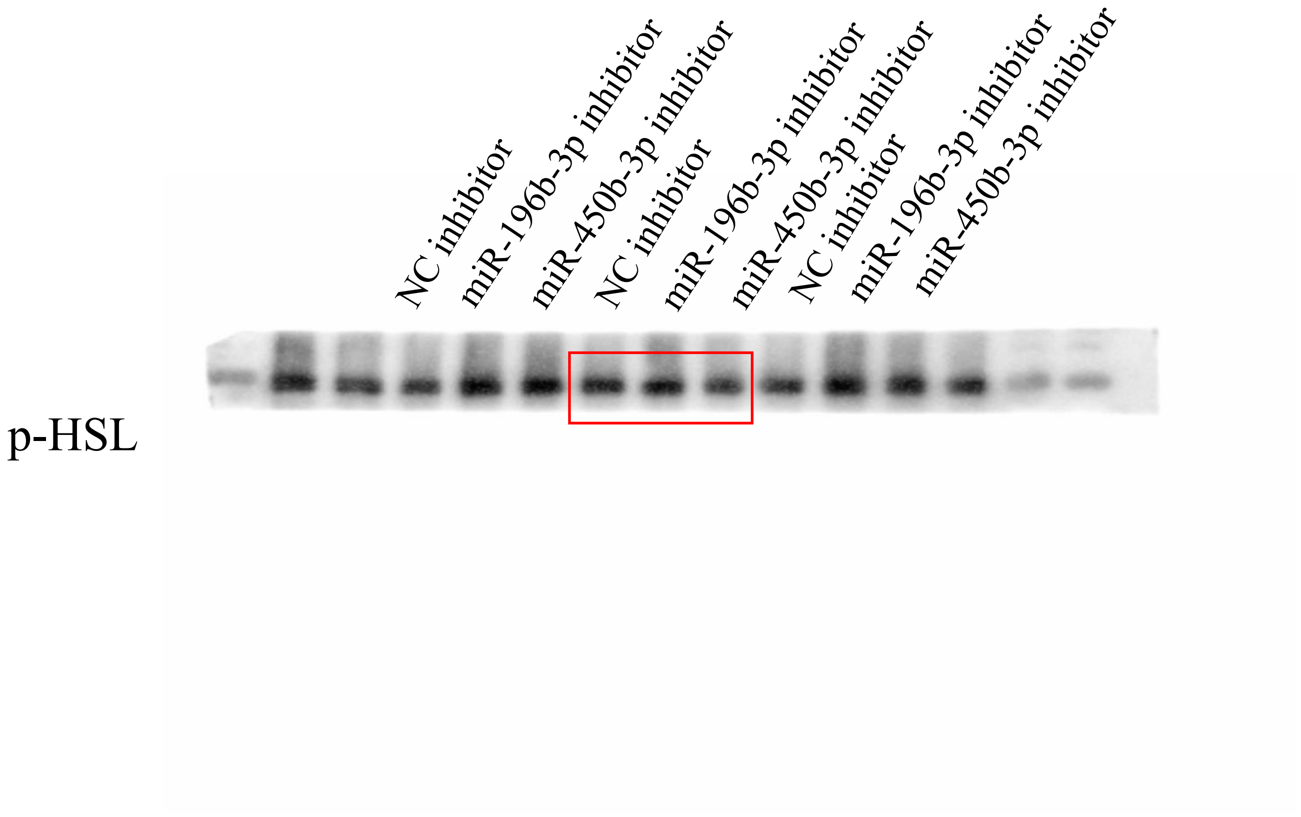
NC inhibitor
miR-196b-3p inhibitor
miR-450b-3p inhibitor
NC inhibitor
miR-196b-3p inhibitor
miR-450b-3p inhibitor
NC inhibitor
miR-196b-3p inhibitor
miR-450b-3p inhibitor
p-HSL

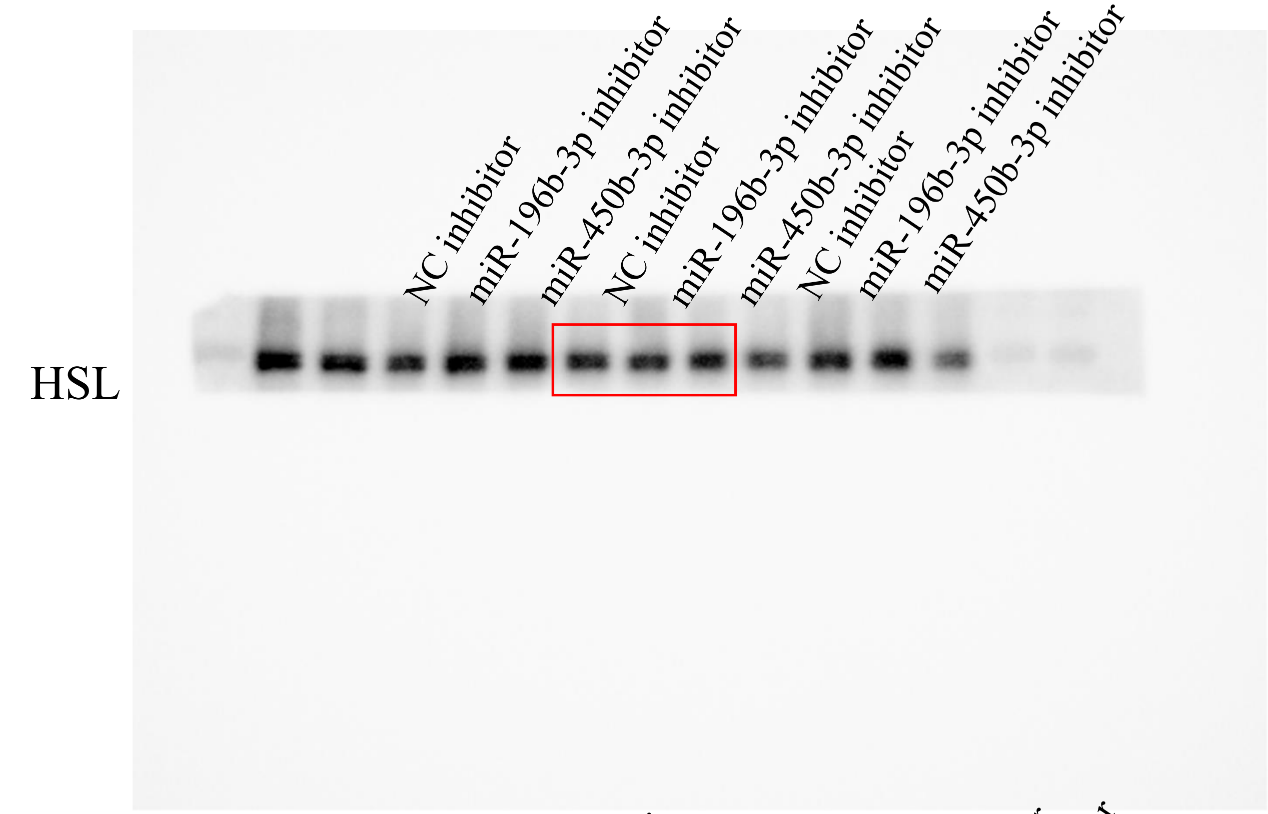
NC inhibitor
miR-196b-3p inhibitor
miR-450b-3p inhibitor
NC inhibitor
miR-196b-3p inhibitor
miR-450b-3p inhibitor
NC inhibitor
miR-196b-3p inhibitor
miR-450b-3p inhibitor
HSL

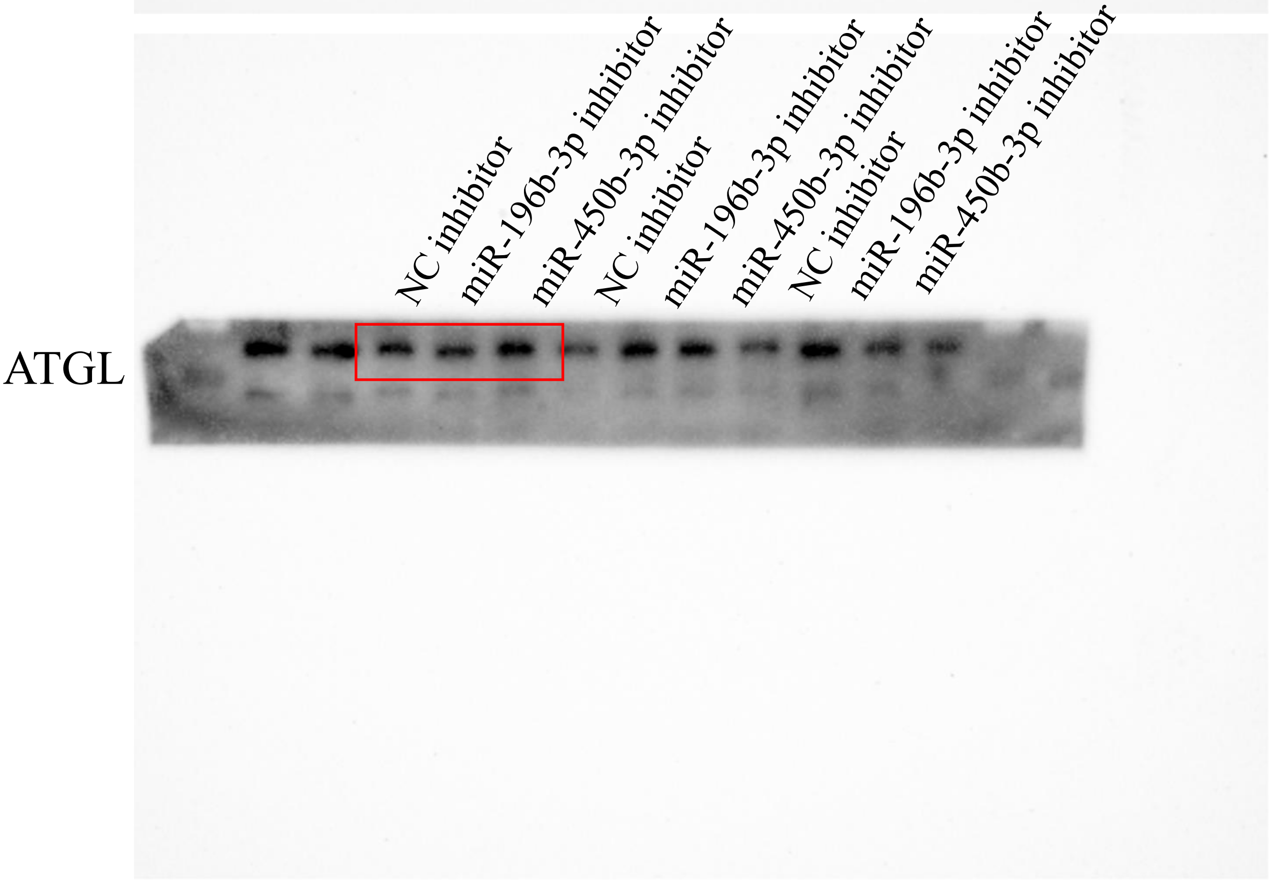
NC inhibitor
miR-196b-3p inhibitor
miR-450b-3p inhibitor
NC inhibitor
miR-196b-3p inhibitor
miR-450b-3p inhibitor
NC inhibitor
miR-196b-3p inhibitor
miR-450b-3p inhibitor
ATGL

aP2

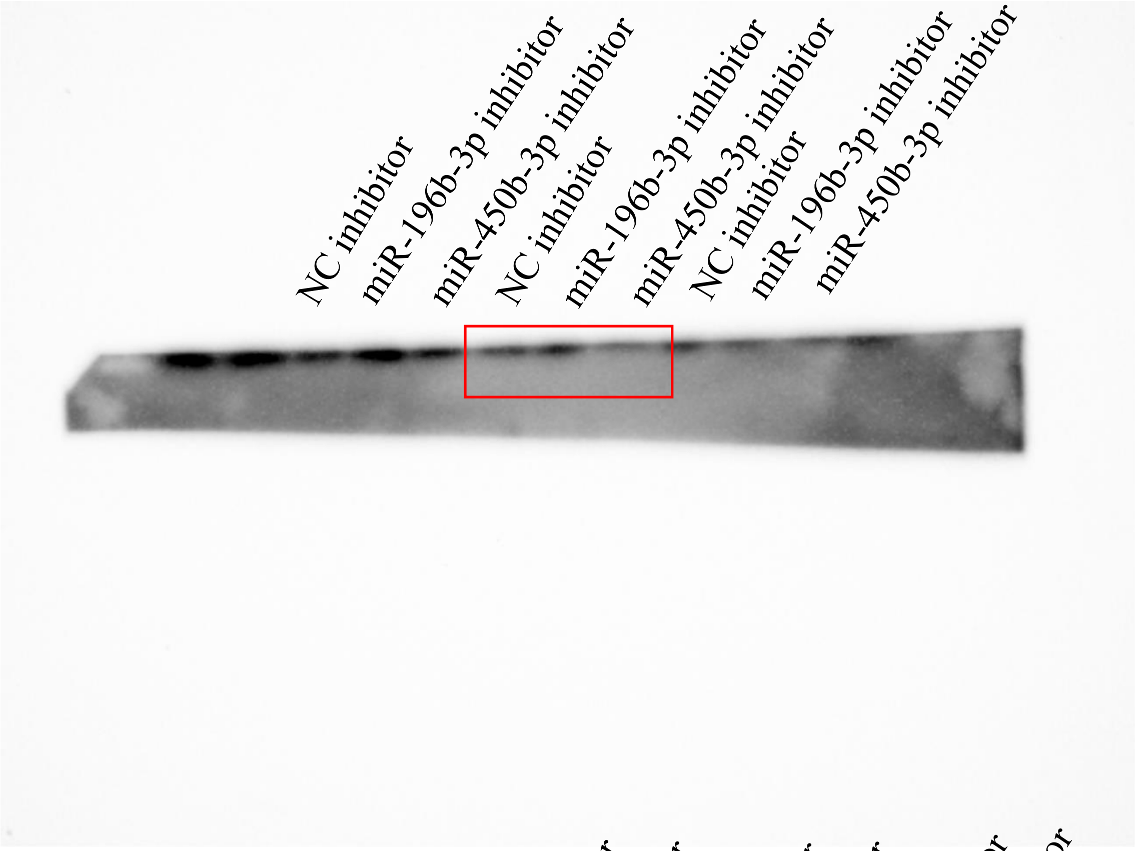

NC inhibitor
miR-196b-3p inhibitor
miR-450b-3p inhibitor
NC inhibitor
miR-196b-3p inhibitor
miR-450b-3p inhibitor
NC inhibitor
miR-196b-3p inhibitor
miR-450b-3p inhibitor

GAPDH

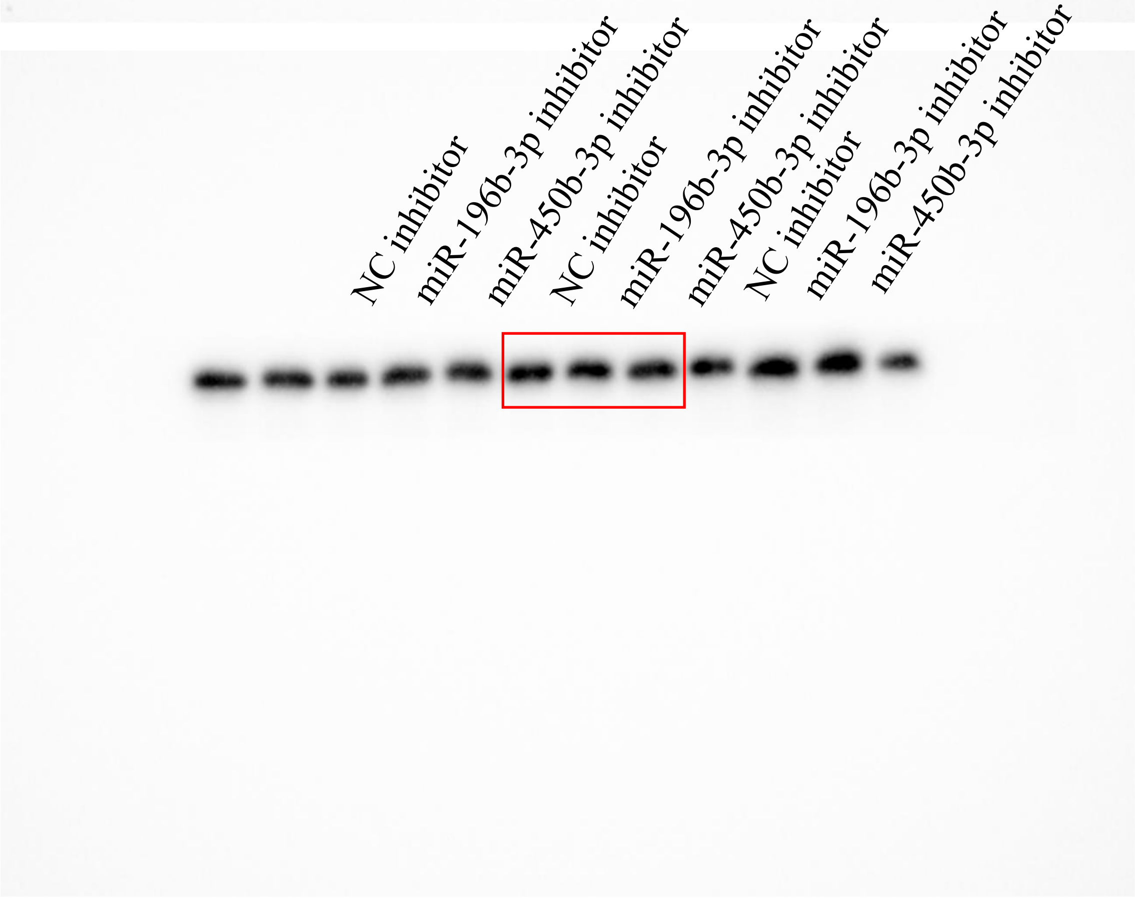

NC inhibitor
miR-196b-3p inhibitor
miR-450b-3p inhibitor
NC inhibitor
miR-196b-3p inhibitor
miR-450b-3p inhibitor
NC inhibitor
miR-196b-3p inhibitor
miR-450b-3p inhibitor

Figure 4

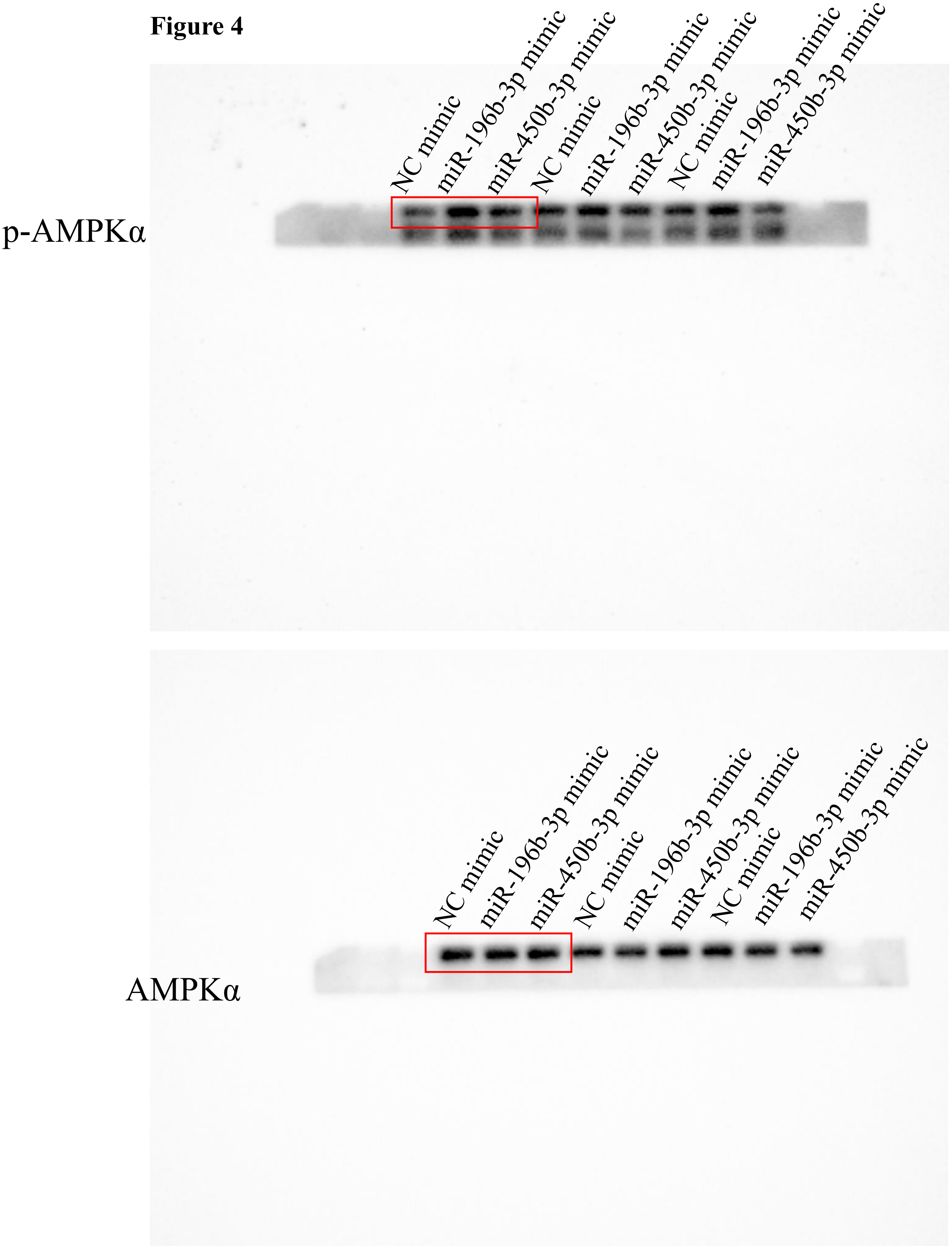
NC mimic
miR-196b-3p mimic
miR-450b-3p mimic
NC mimic
miR-196b-3p mimic
miR-450b-3p mimic
NC mimic
miR-196b-3p mimic
miR-450b-3p mimic
p-AMPKα
NC mimic
miR-196b-3p mimic
miR-450b-3p mimic
NC mimic
miR-196b-3p mimic
miR-450b-3p mimic
NC mimic
miR-196b-3p mimic
miR-450b-3p mimic
AMPKα

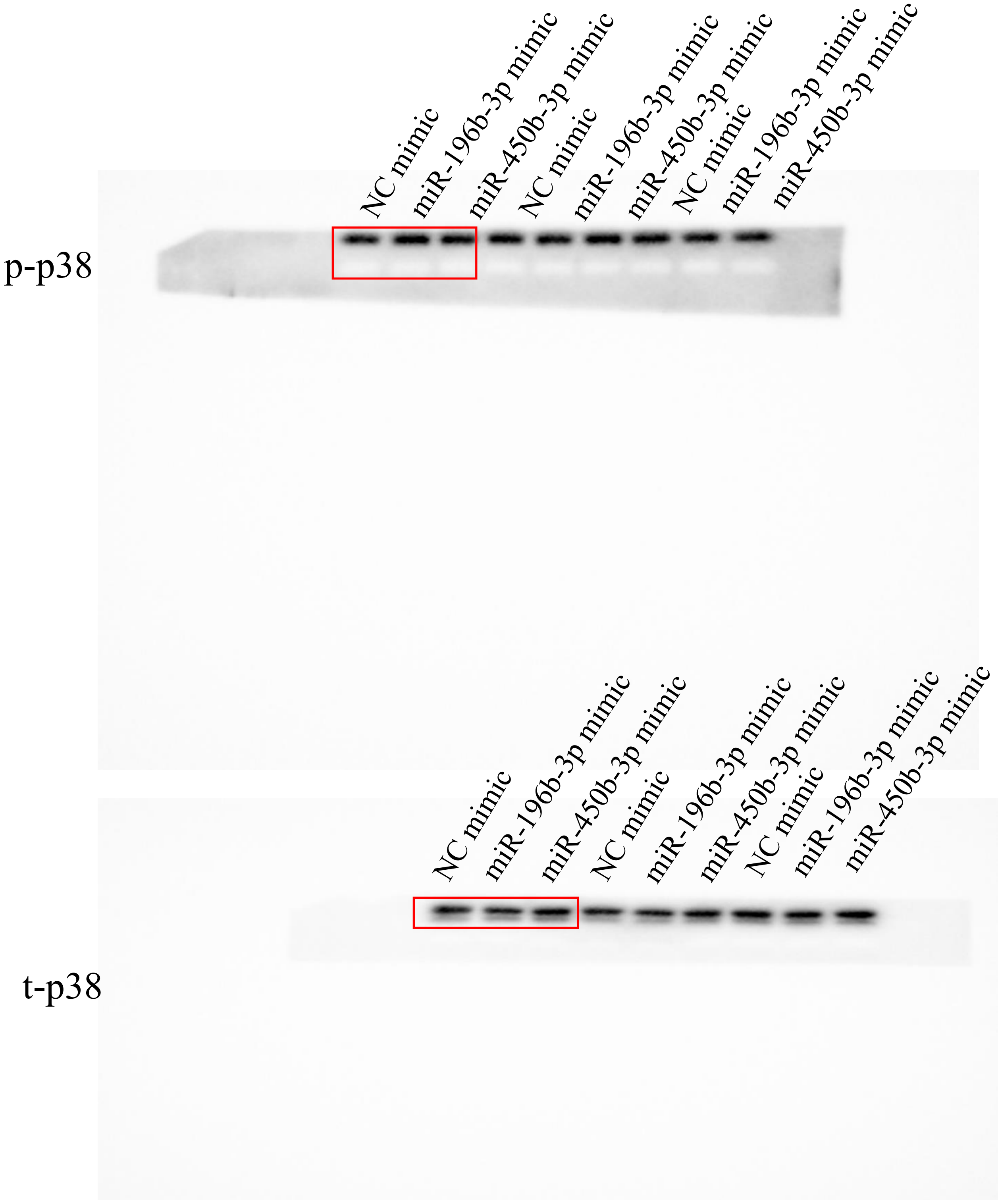

NC mimic
miR-196b-3p mimic
miR-450b-3p mimic
NC mimic
miR-196b-3p mimic
miR-450b-3p mimic
NC mimic
miR-196b-3p mimic
miR-450b-3p mimic
p-p38
NC mimic
miR-196b-3p mimic
miR-450b-3p mimic
NC mimic
miR-196b-3p mimic
miR-450b-3p mimic
NC mimic
miR-196b-3p mimic
miR-450b-3p mimic
t-p38

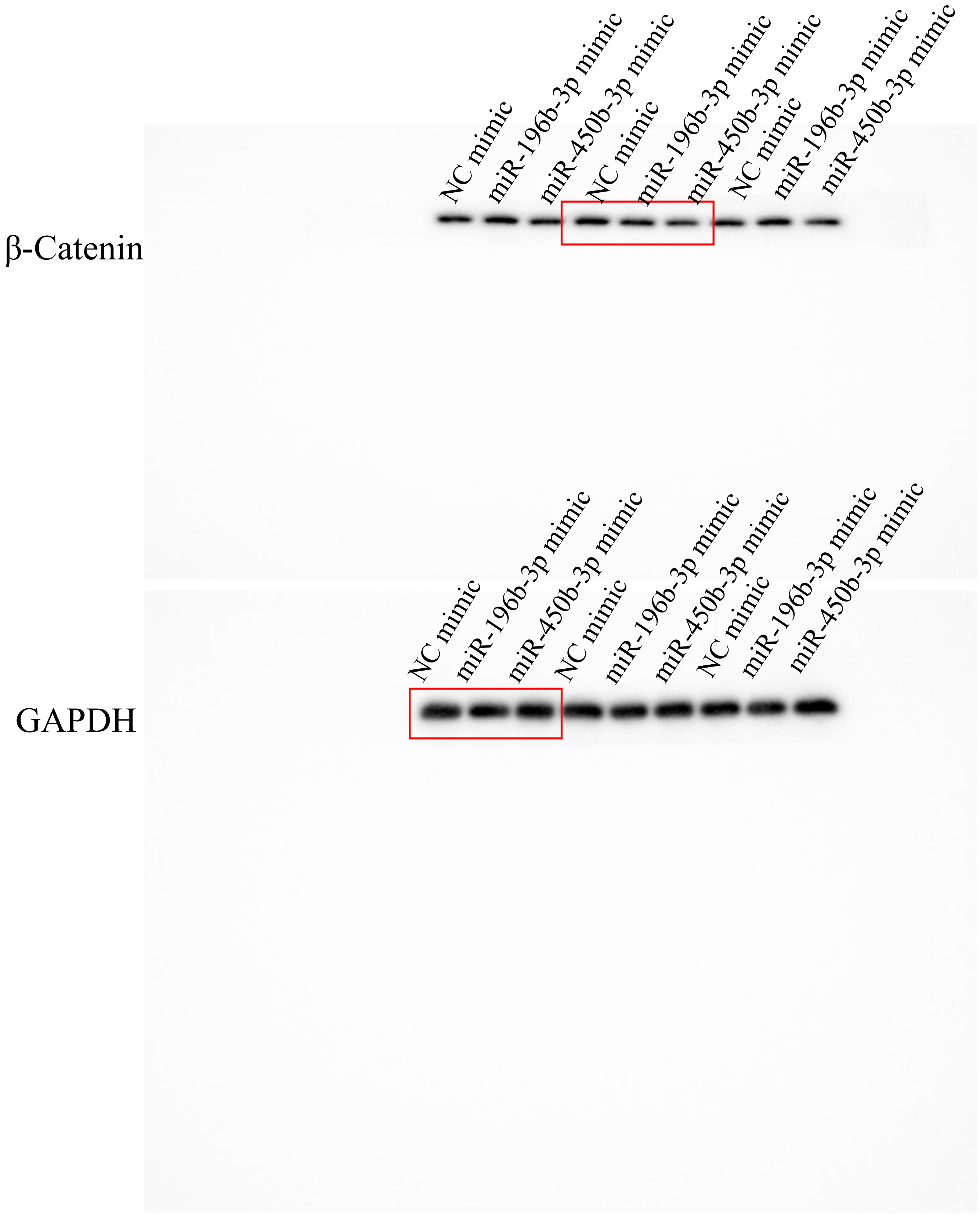
β-Catenin
GAPDH
NC mimic
miR-196b-3p mimic
miR-450b-3p mimic
NC mimic
miR-196b-3p mimic
miR-450b-3p mimic
NC mimic
miR-196b-3p mimic
miR-450b-3p mimic

p-AMPKα

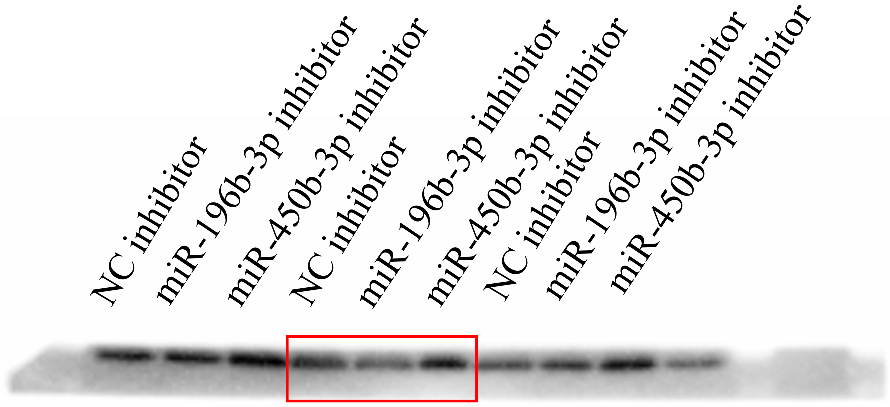

AMPKα

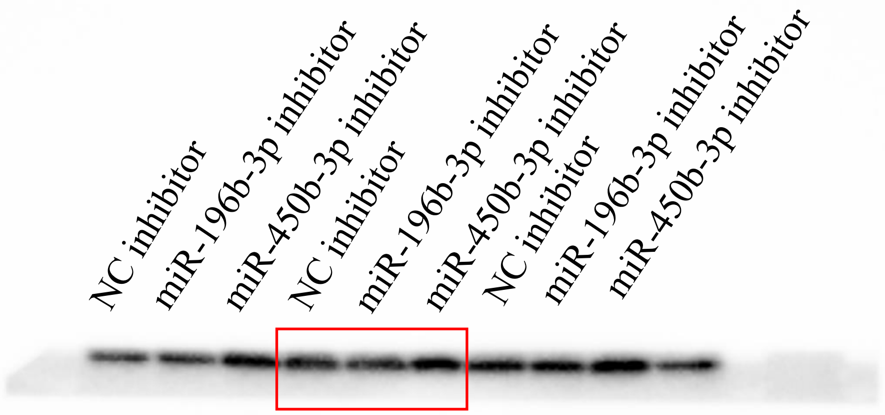

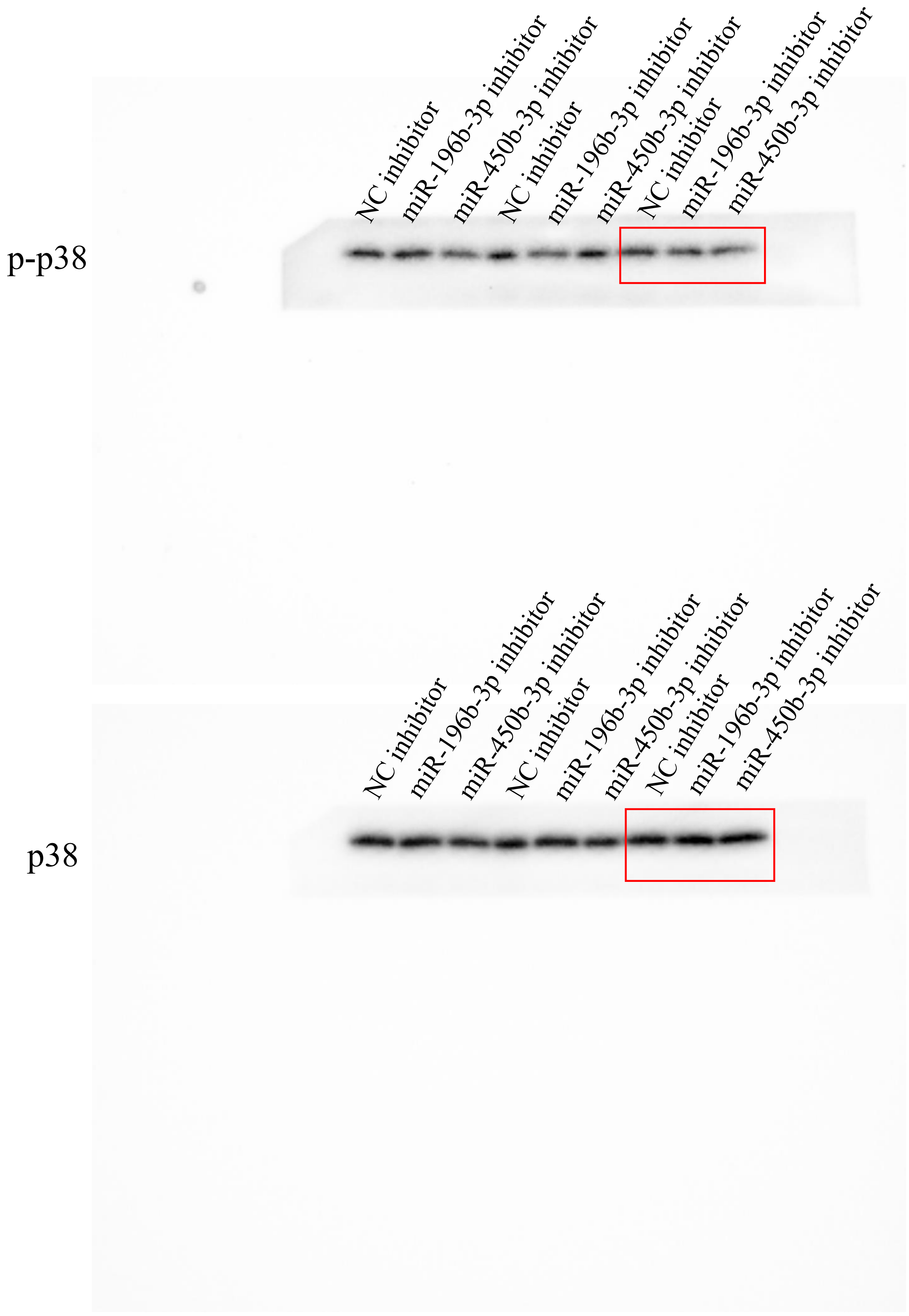
NC inhibitor
miR-196b-3p inhibitor
miR-450b-3p inhibitor
NC inhibitor
miR-196b-3p inhibitor
miR-450b-3p inhibitor
NC inhibitor
miR-196b-3p inhibitor
miR-450b-3p inhibitor
p-p38
NC inhibitor
miR-196b-3p inhibitor
miR-450b-3p inhibitor
NC inhibitor
miR-196b-3p inhibitor
miR-450b-3p inhibitor
NC inhibitor
miR-196b-3p inhibitor
miR-450b-3p inhibitor
p38

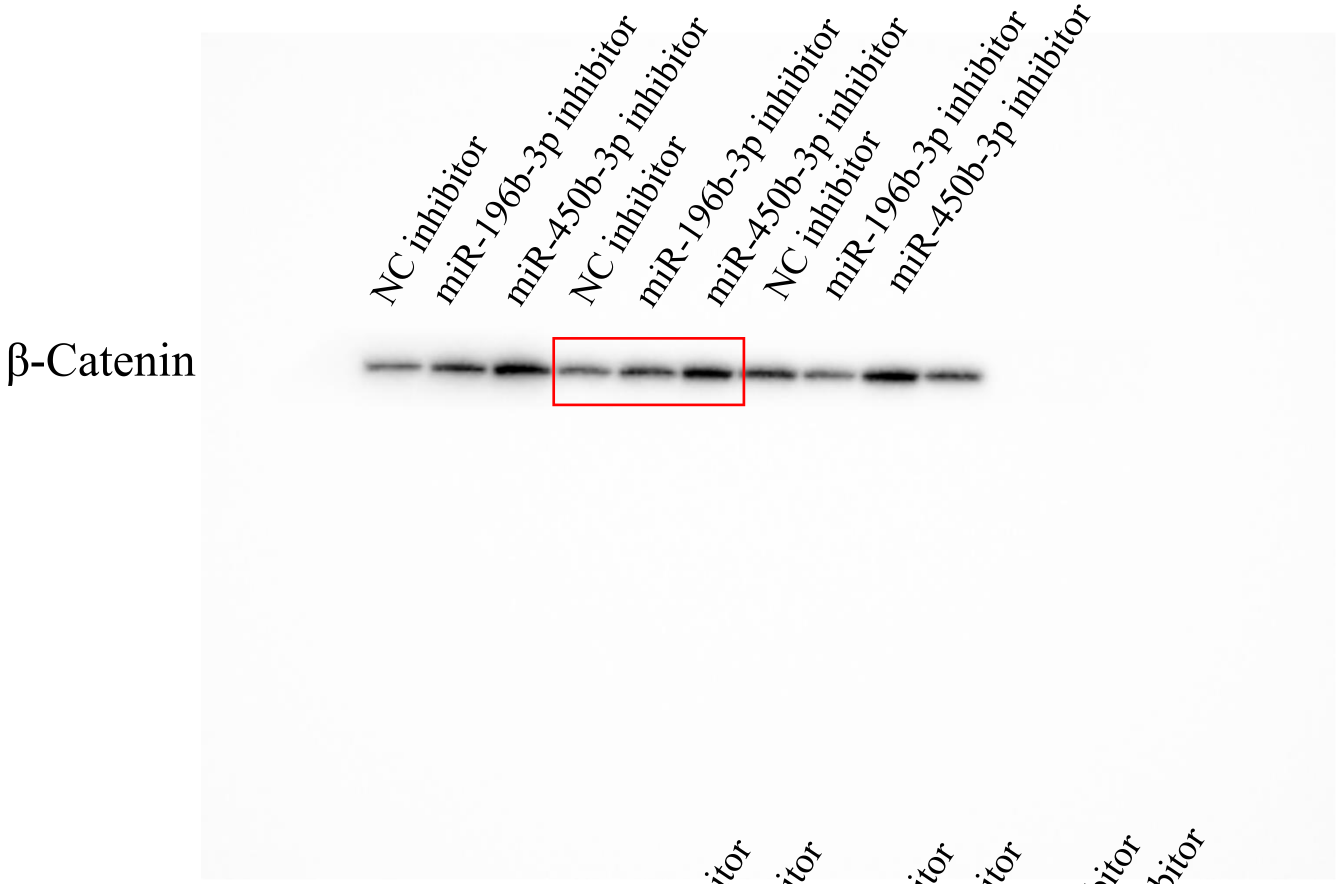
NC inhibitor
miR-196b-3p inhibitor
miR-450b-3p inhibitor
NC inhibitor
miR-196b-3p inhibitor
miR-450b-3p inhibitor
NC inhibitor
miR-196b-3p inhibitor
miR-450b-3p inhibitor
β-Catenin

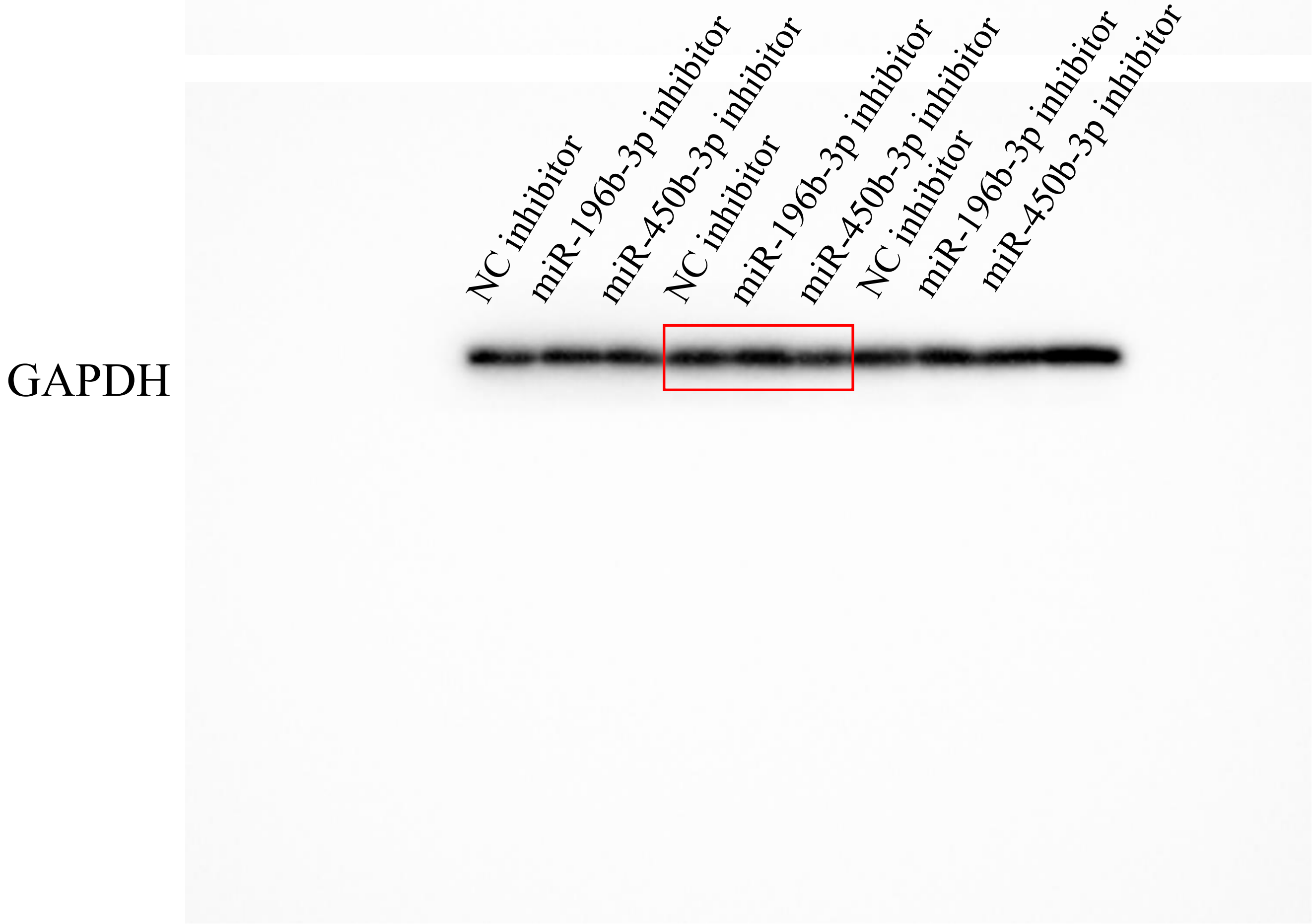
NC inhibitor
miR-196b-3p inhibitor
miR-450b-3p inhibitor
NC inhibitor
miR-196b-3p inhibitor
miR-450b-3p inhibitor
NC inhibitor
miR-196b-3p inhibitor
miR-450b-3p inhibitor
GAPDH

Figure 5

p-AMPKα

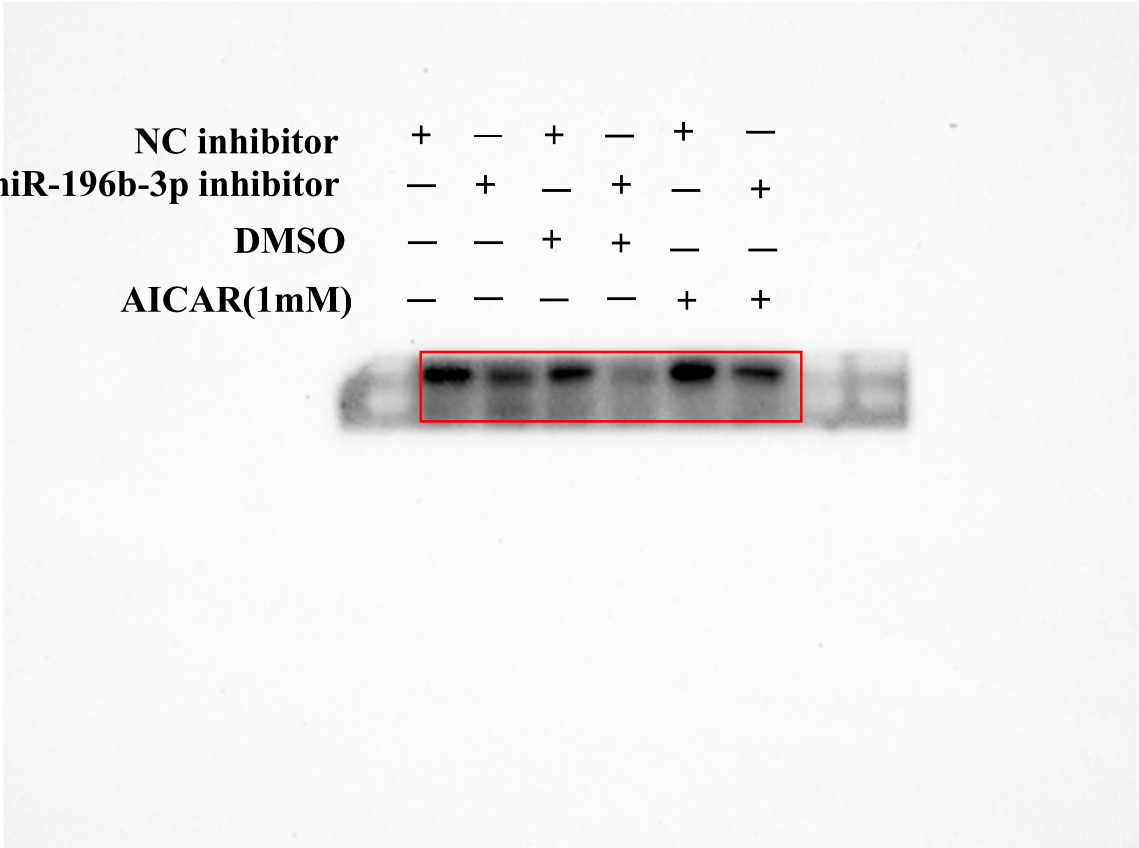

p-AMPKα

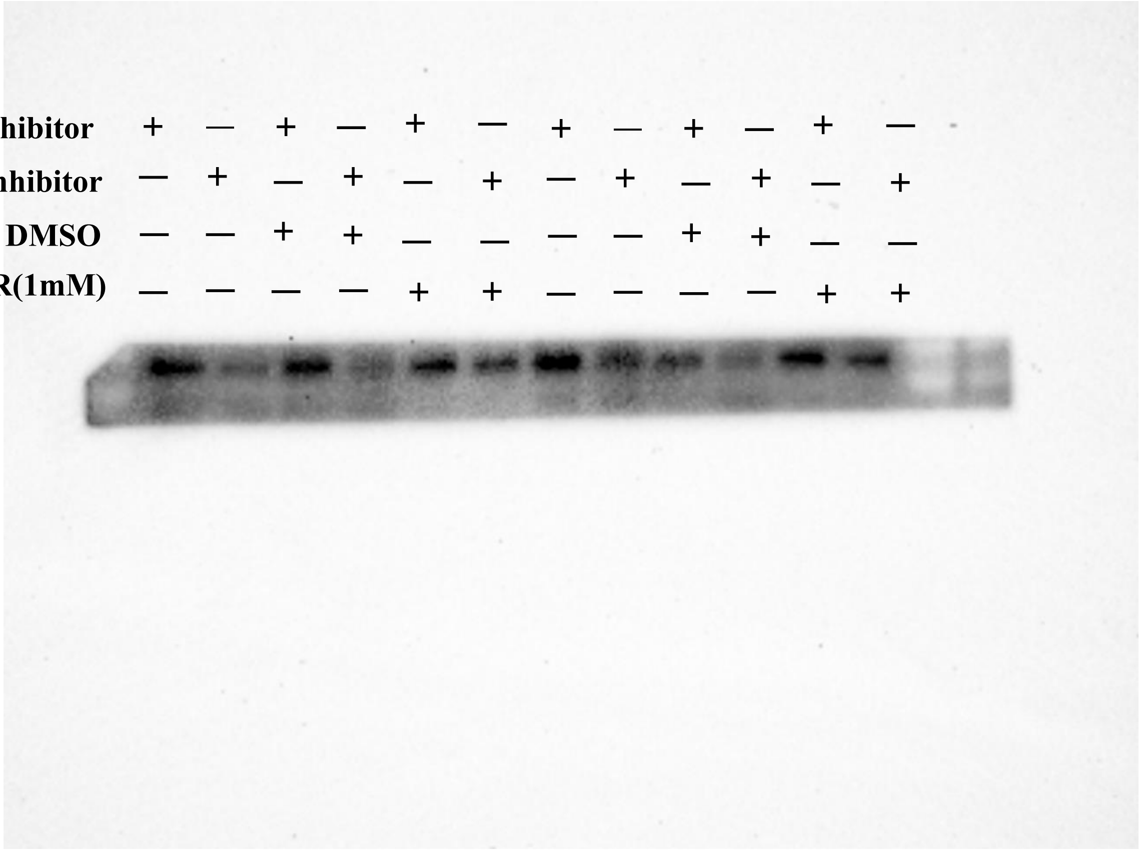

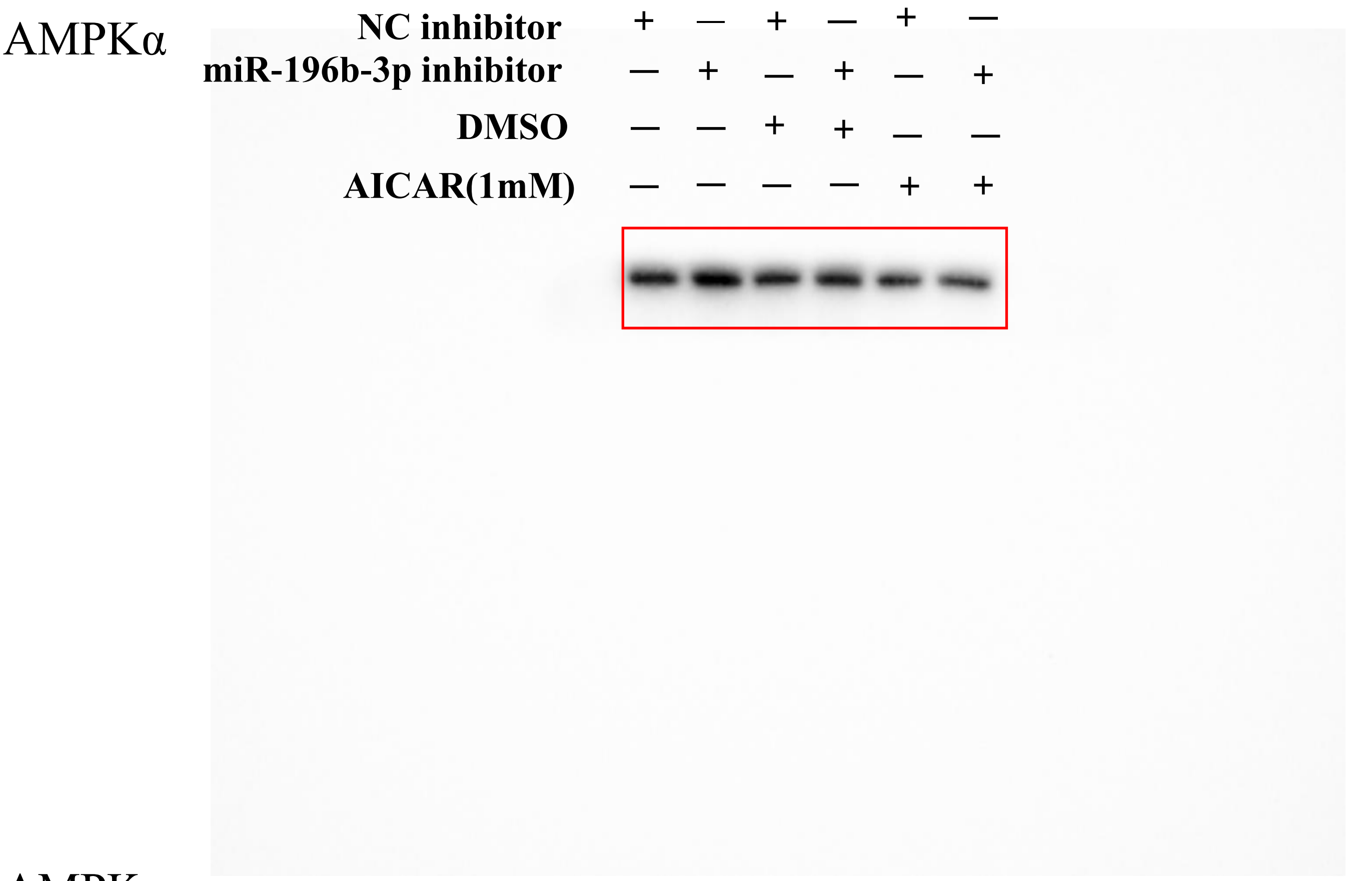
AMPKα
NC inhibitor + — + — + —
miR-196b-3p inhibitor — + — + — +
DMSO — — + + — —
AICAR(1mM) — — — — + +

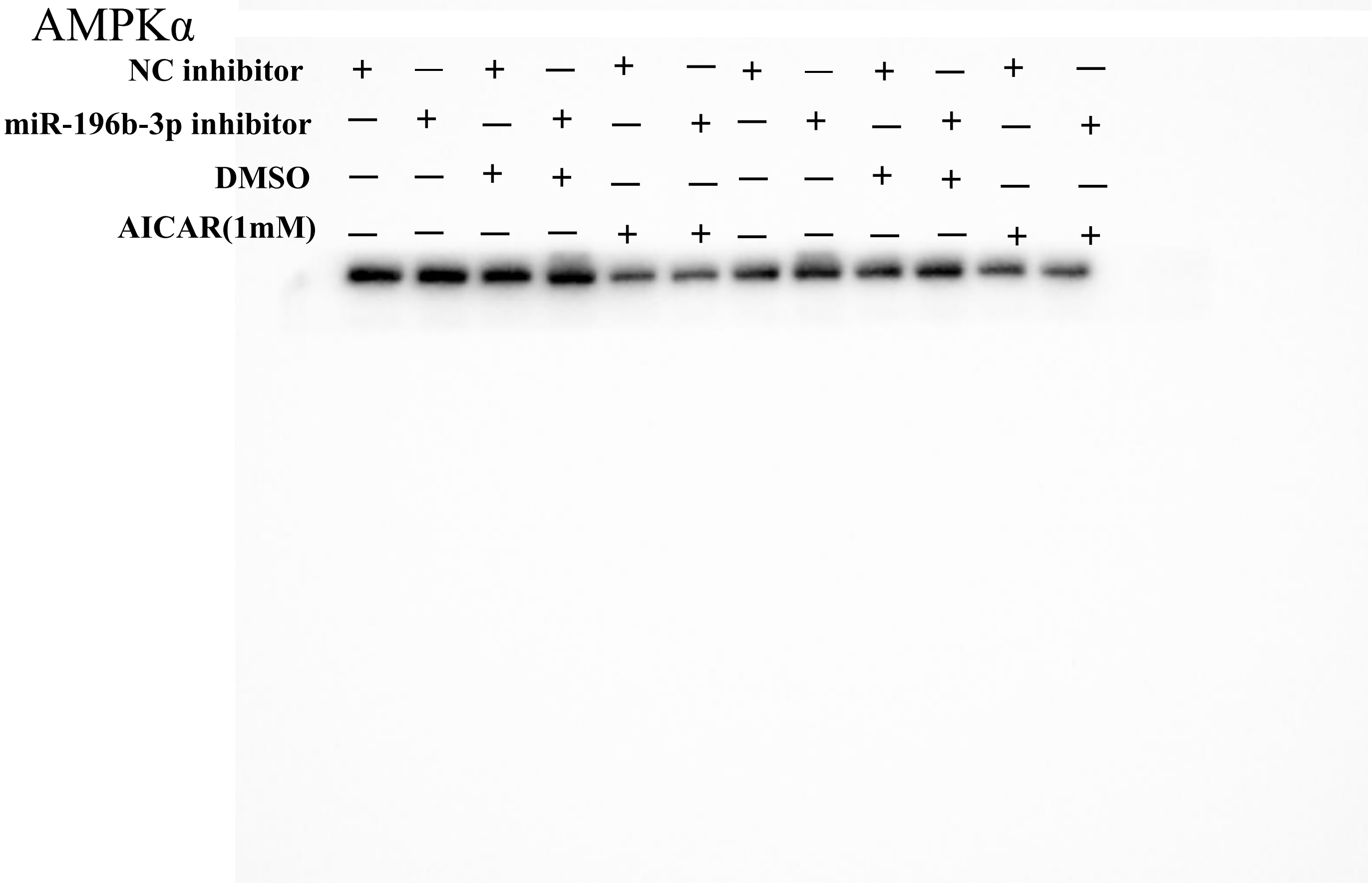
AMPKα
NC inhibitor + — + — + — + — + — + —
miR-196b-3p inhibitor — + — + — + — + — + — +
DMSO — — + + — — — — + + — —
AICAR(1mM) — — — — + + — — — — + +

β-actin

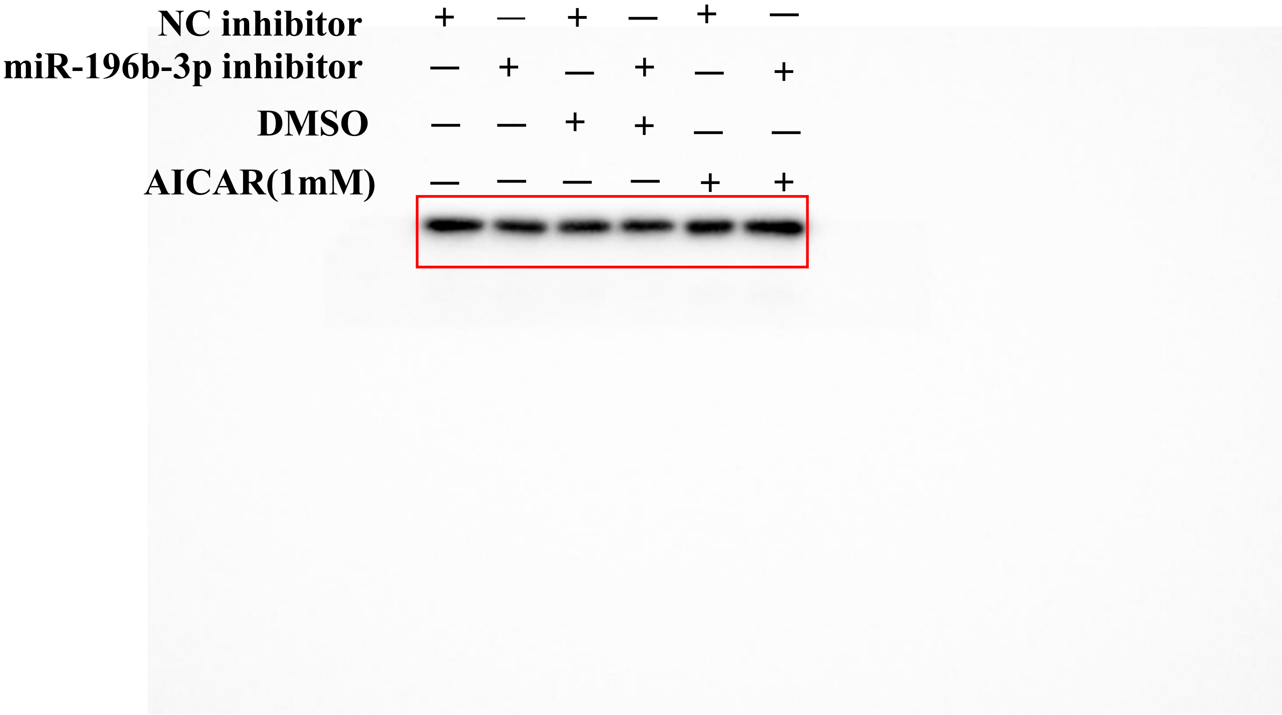

β-actin

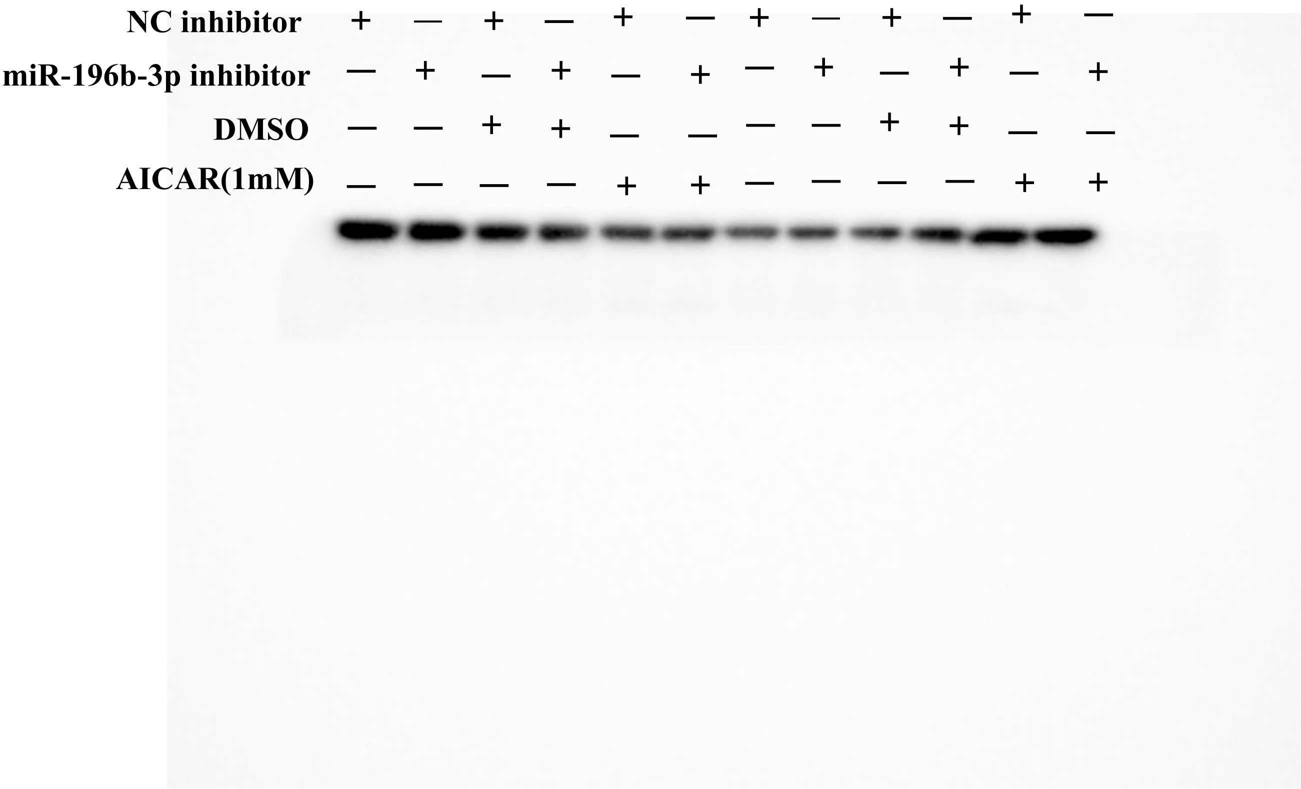

aP2

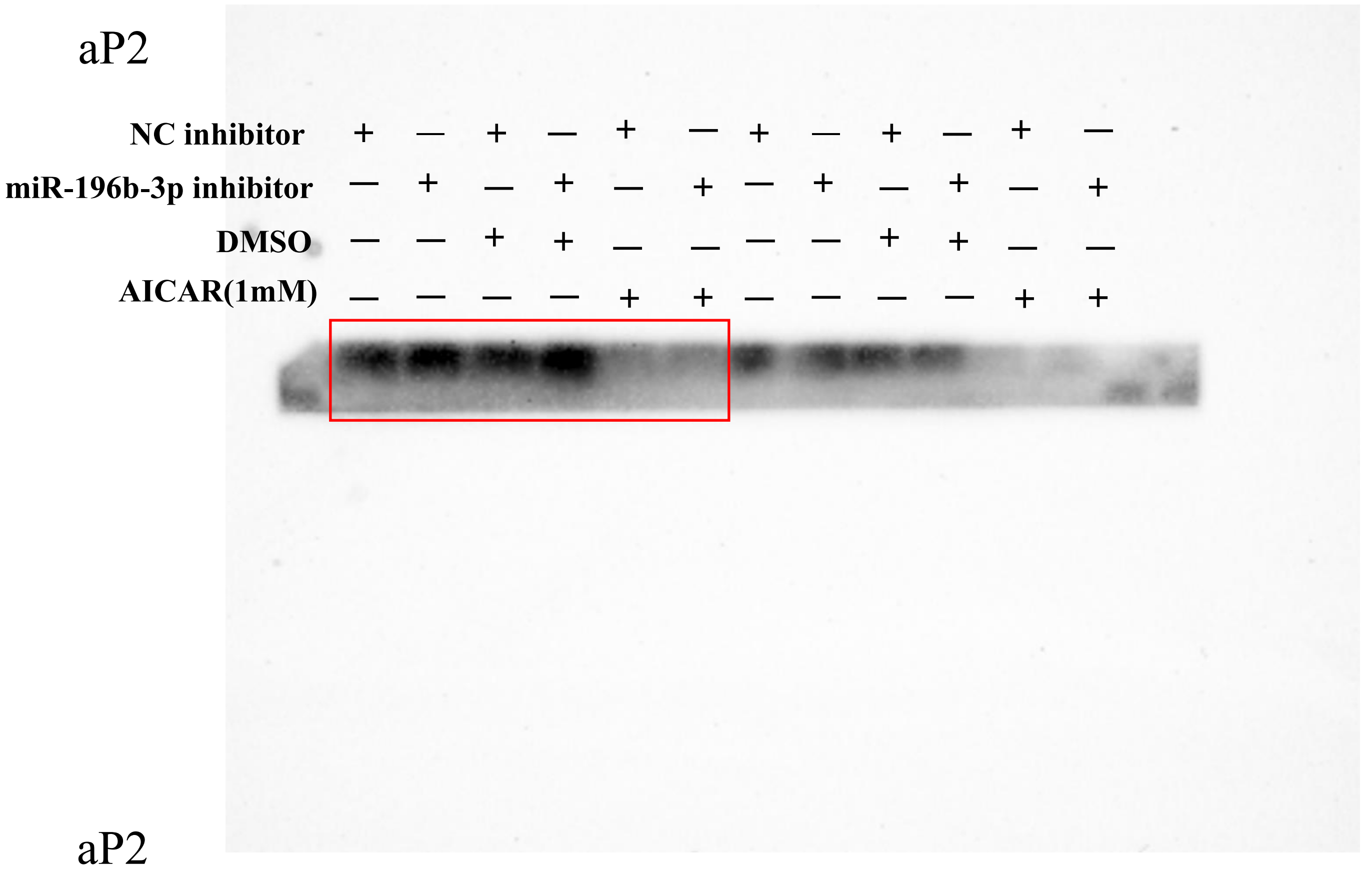
NC inhibitor + — + — + — + — + — + —
miR-196b-3p inhibitor — + — + — + — + — + — +
DMSO — — + + — — — — + + — —
AICAR(1mM) — — — — + + — — — — + +

aP2

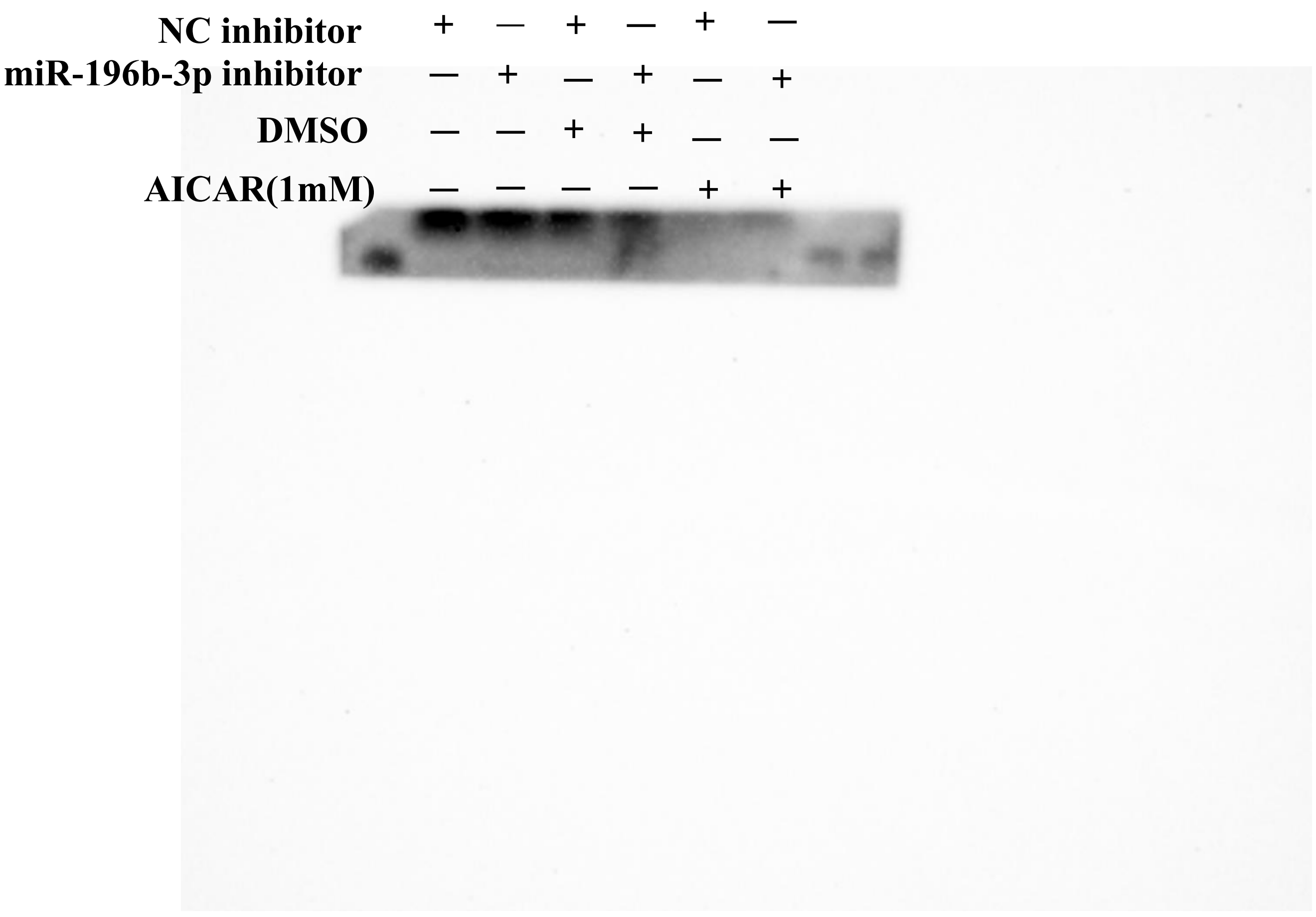
NC inhibitor + — + — + —
miR-196b-3p inhibitor — + — + — +
DMSO — — + + — —
AICAR(1mM) — — — — + +

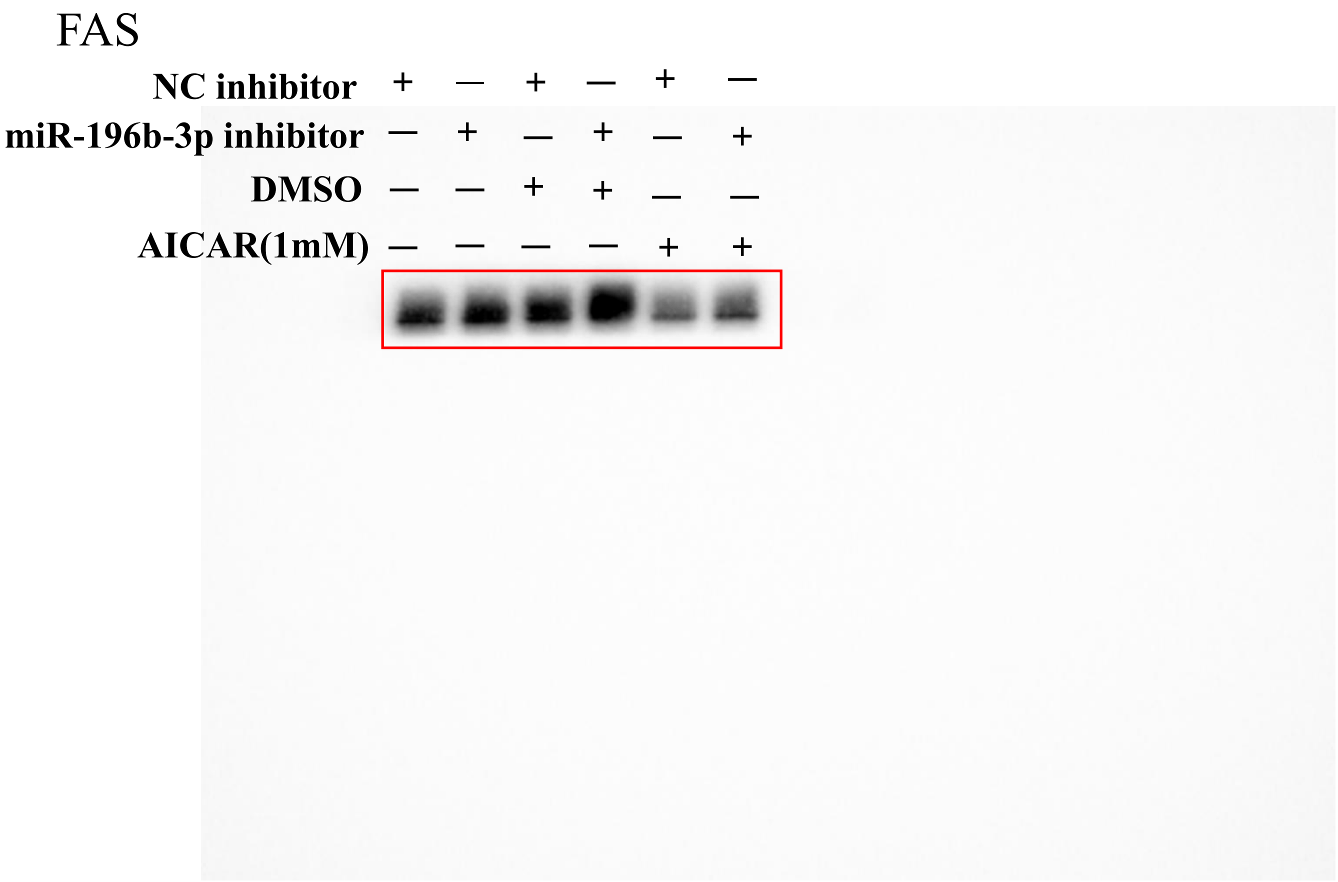
FAS
NC inhibitor + — + — + —
miR-196b-3p inhibitor — + — + — +
DMSO — — + + — —
AICAR(1mM) — — — — + +

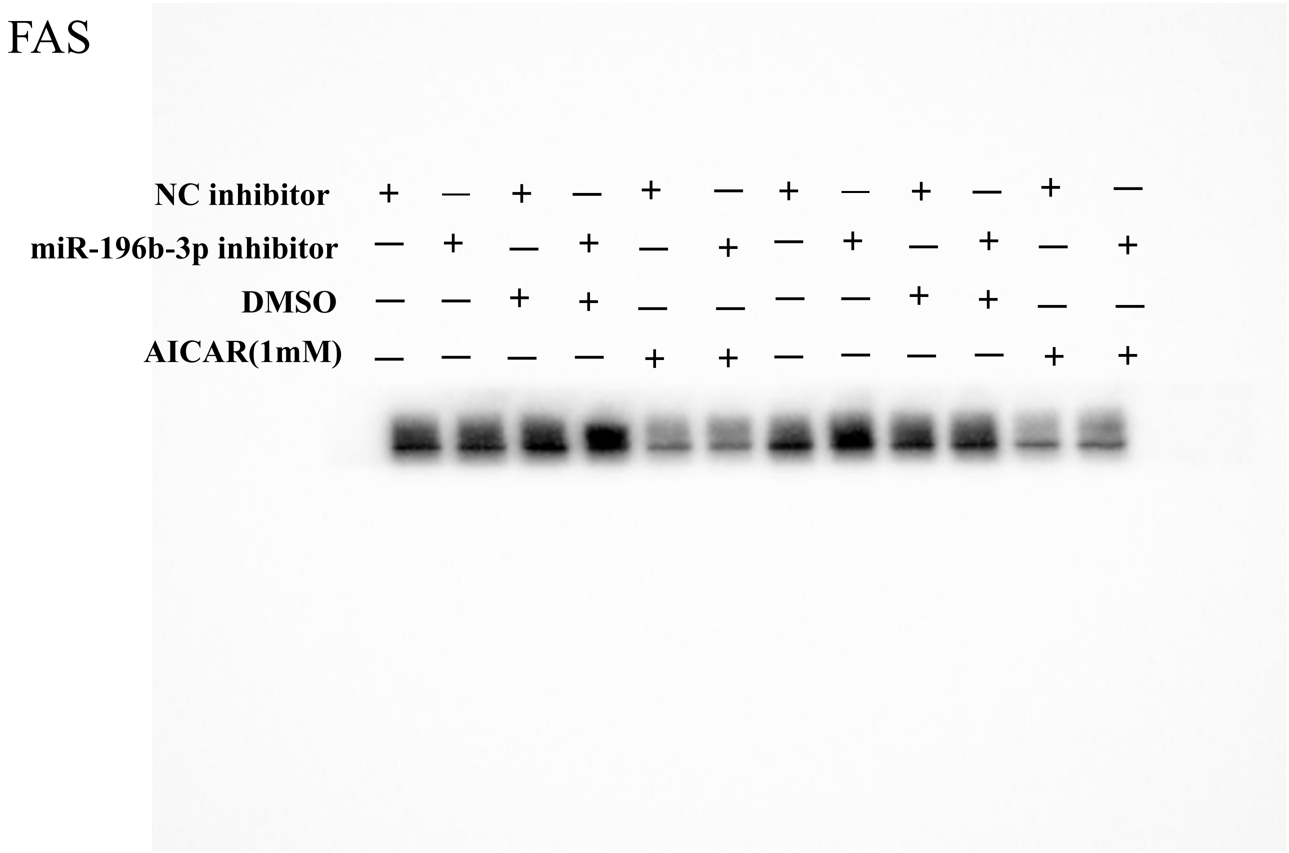
FAS
NC inhibitor + — + — + — + — + — + —
miR-196b-3p inhibitor — + — + — + — + — + — +
DMSO — — + + — — — — + + — —
AICAR(1mM) — — — — + + — — — — + +

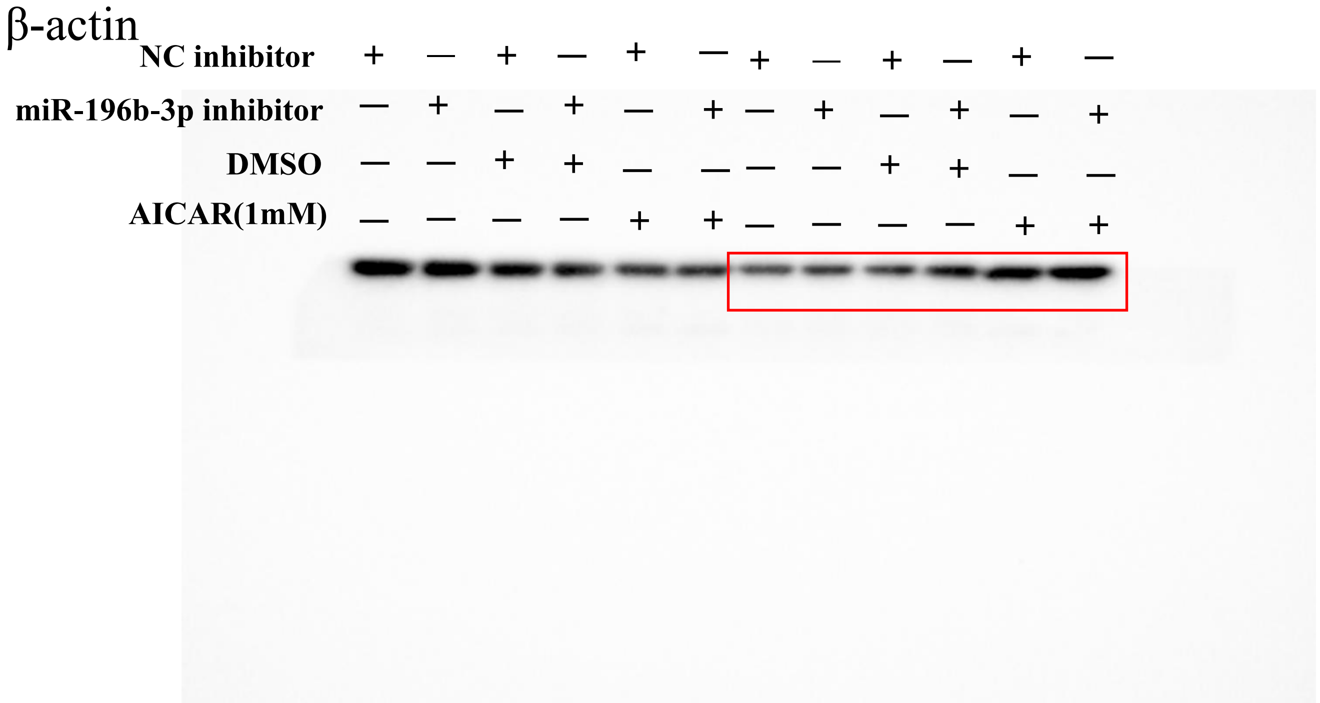
β-actin
NC inhibitor
miR-196b-3p inhibitor
DMSO
AICAR(1mM)

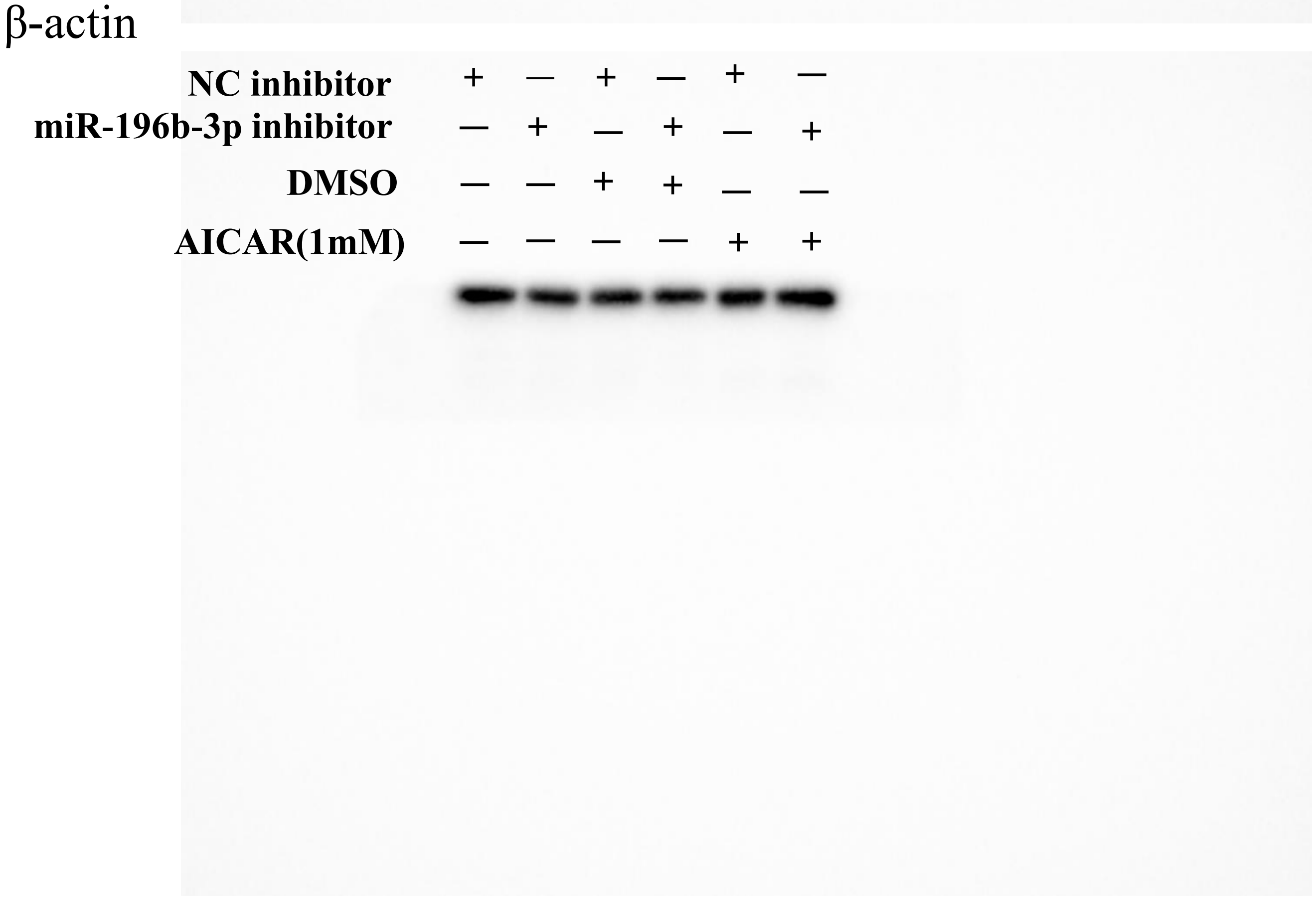
β-actin
NC inhibitor
miR-196b-3p inhibitor
DMSO
AICAR(1mM)

Figure 6

cellular protein

β-Catenin

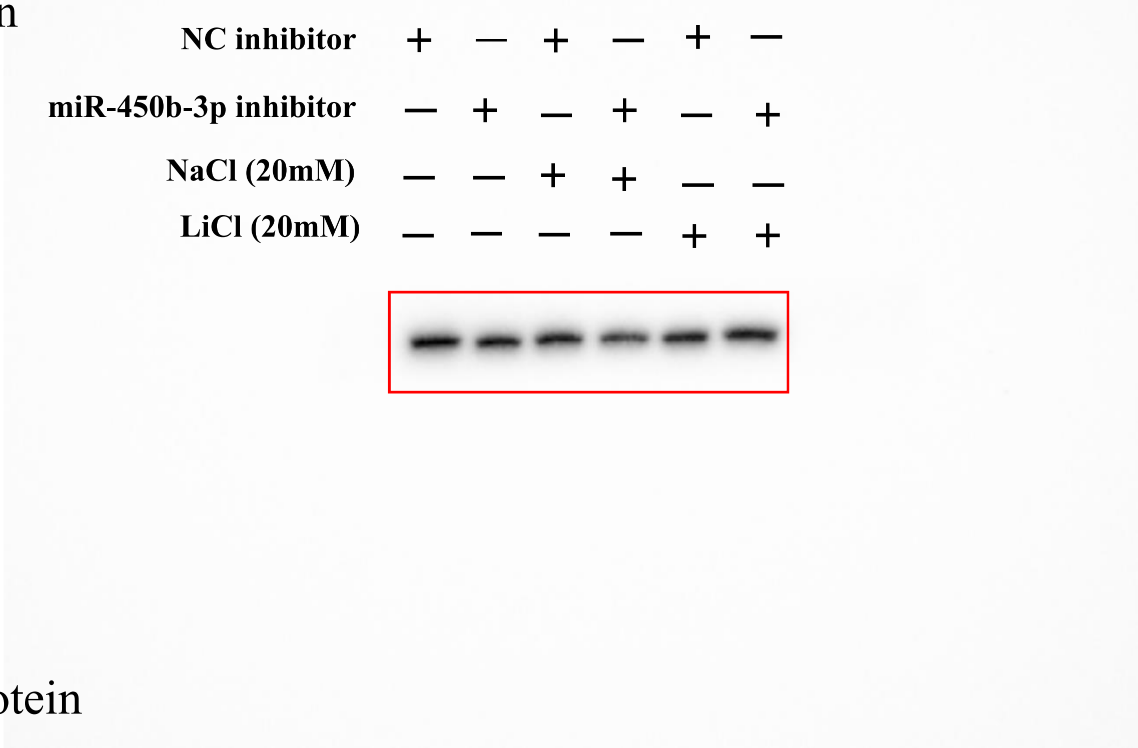

cellular protein

β-Catenin

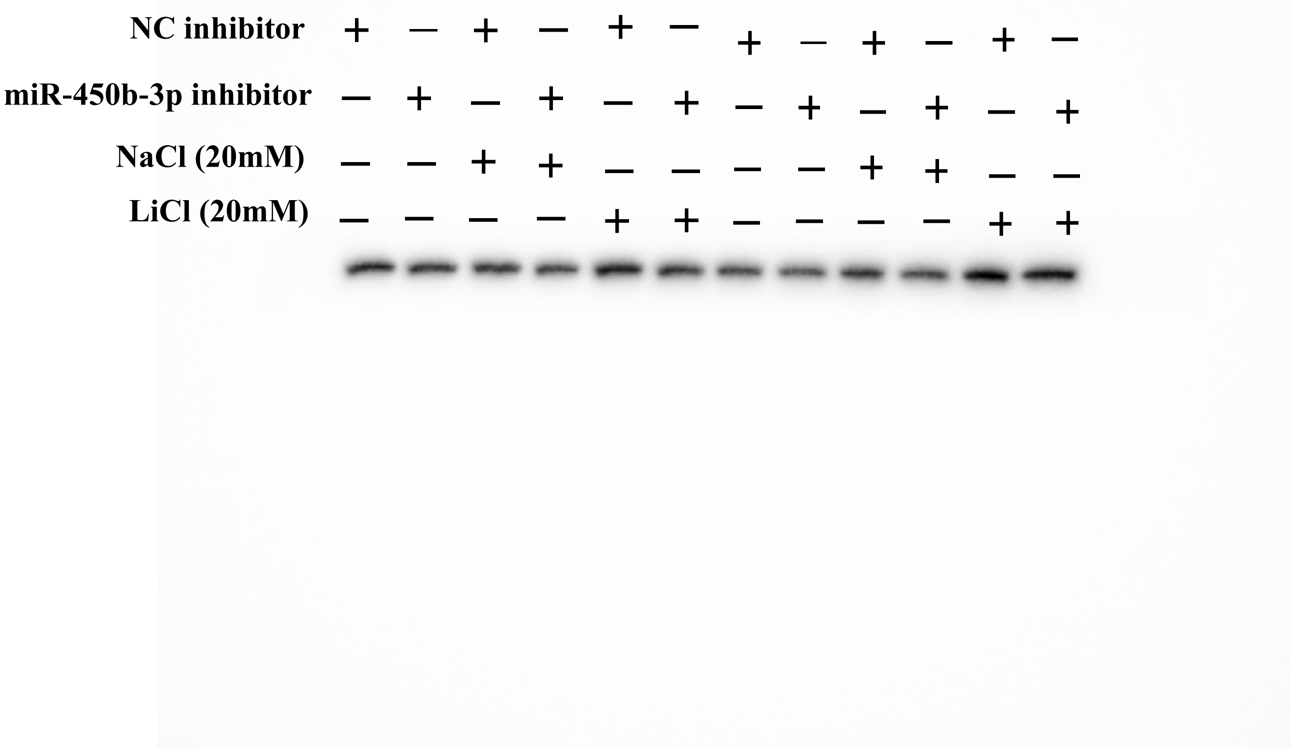

cellular protein

GAPDH

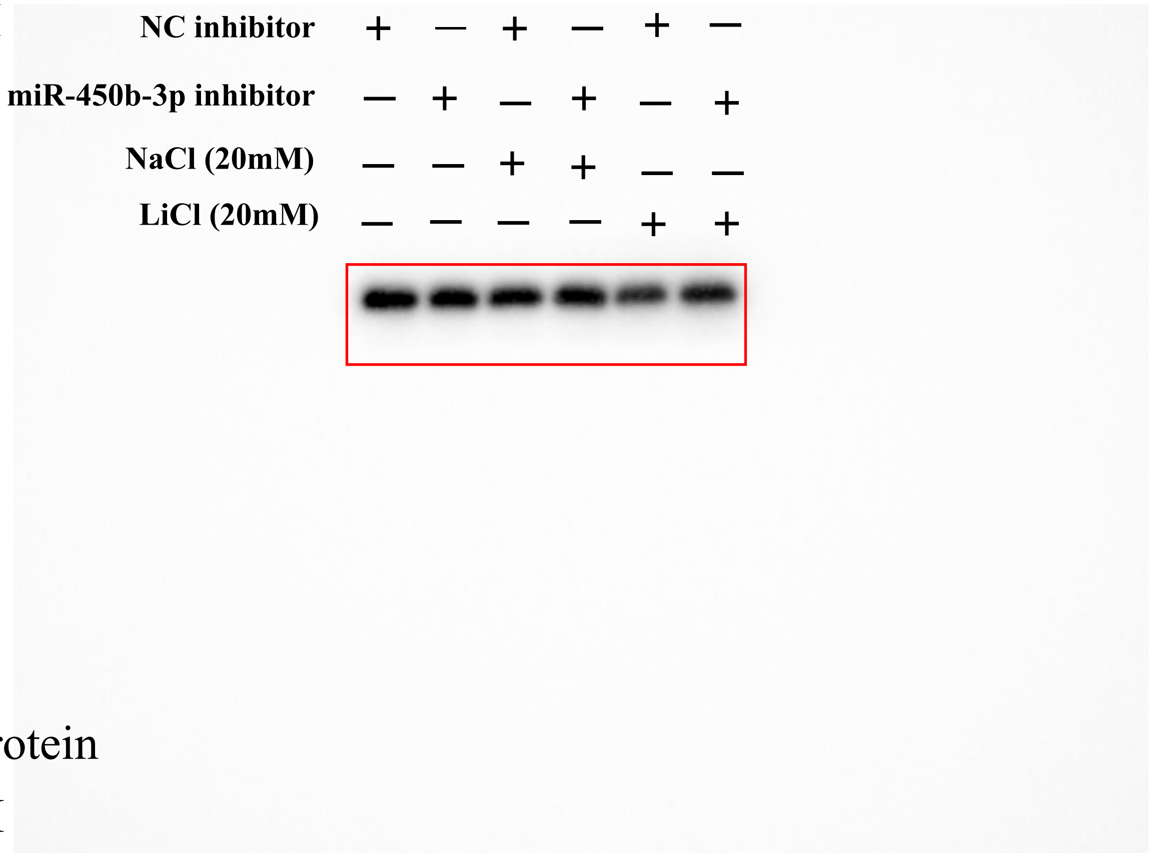

cellular protein

GAPDH

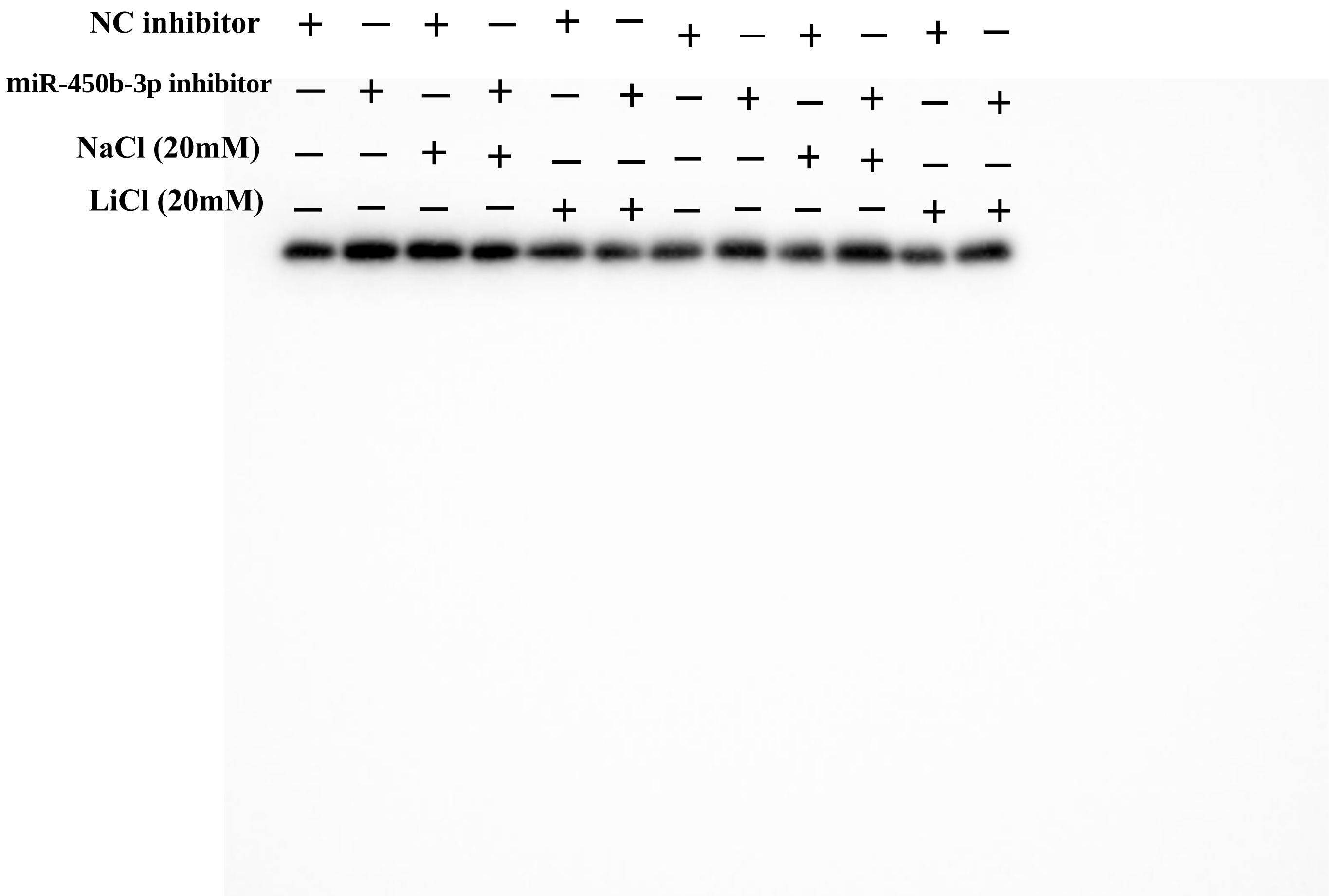

Nuclear protein

β-Catenin

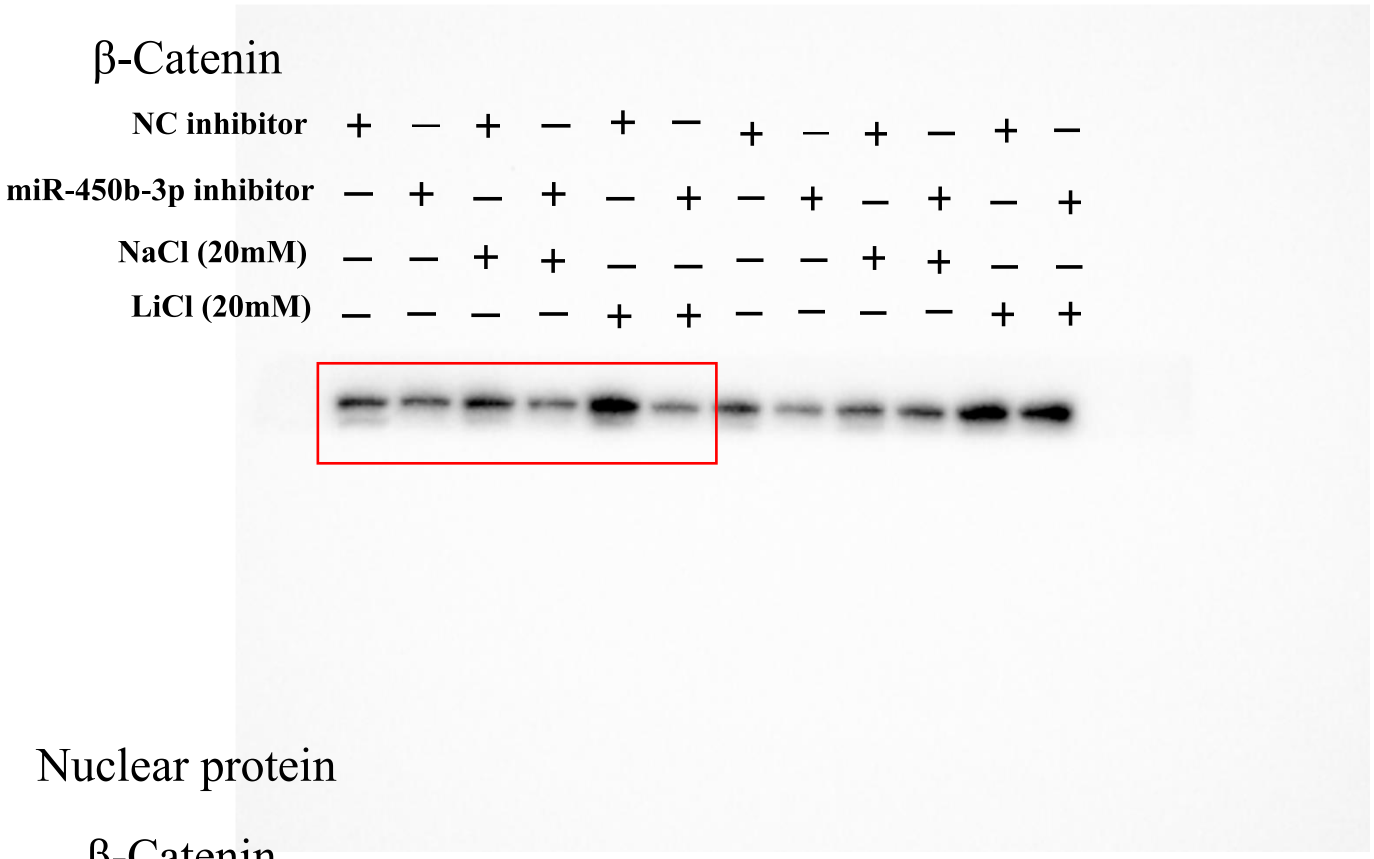

Nuclear protein

β-Catenin

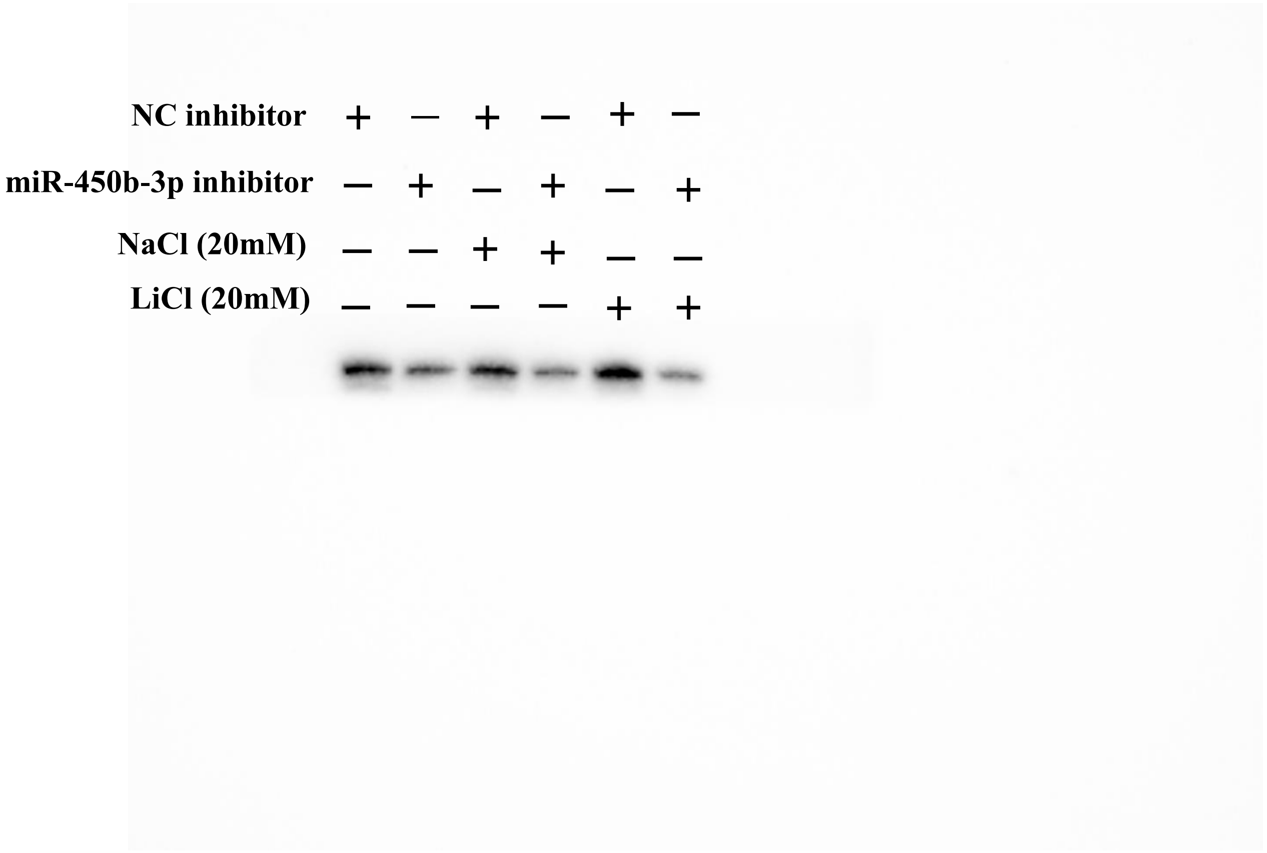

Nuclear protein

Lamin B1

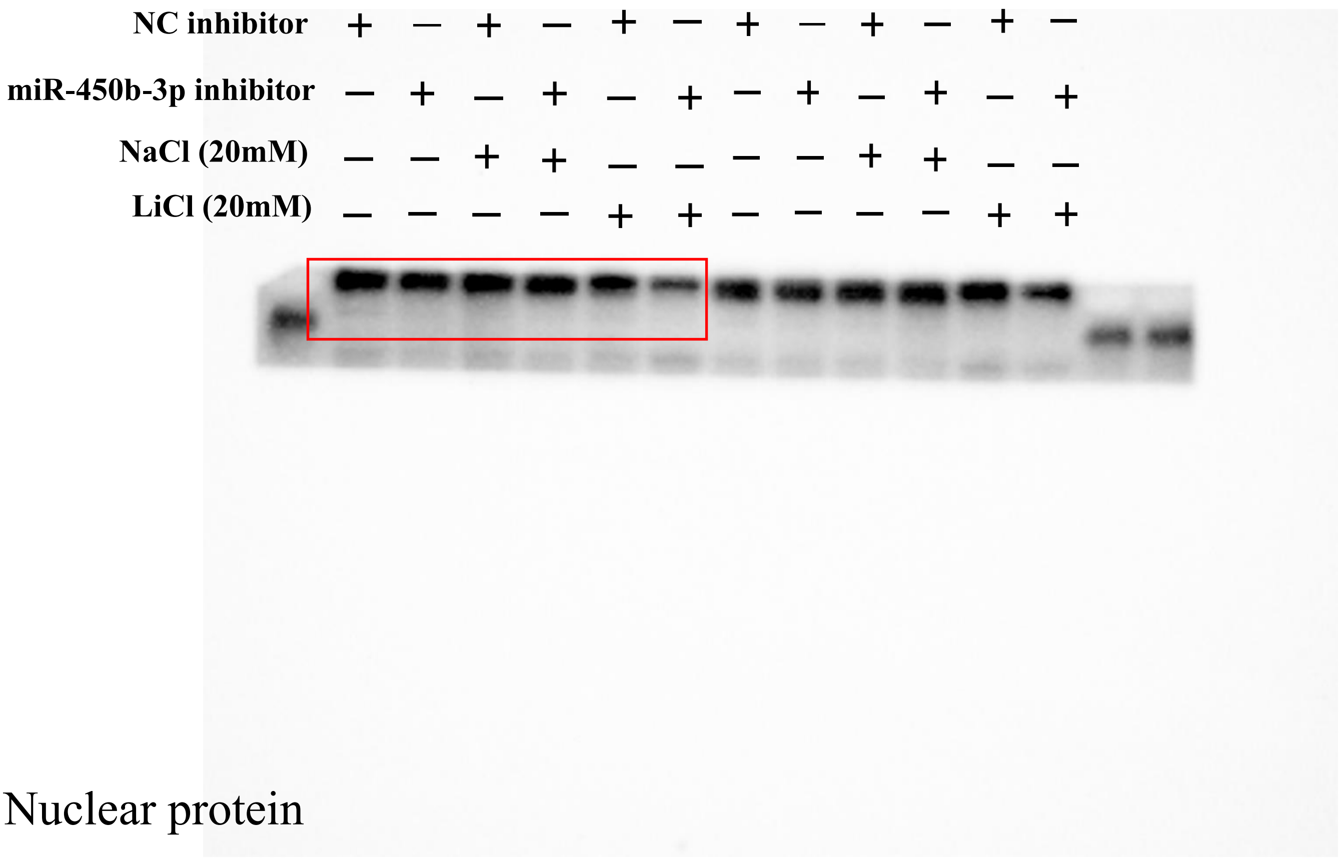

Nuclear protein

Lamin B1

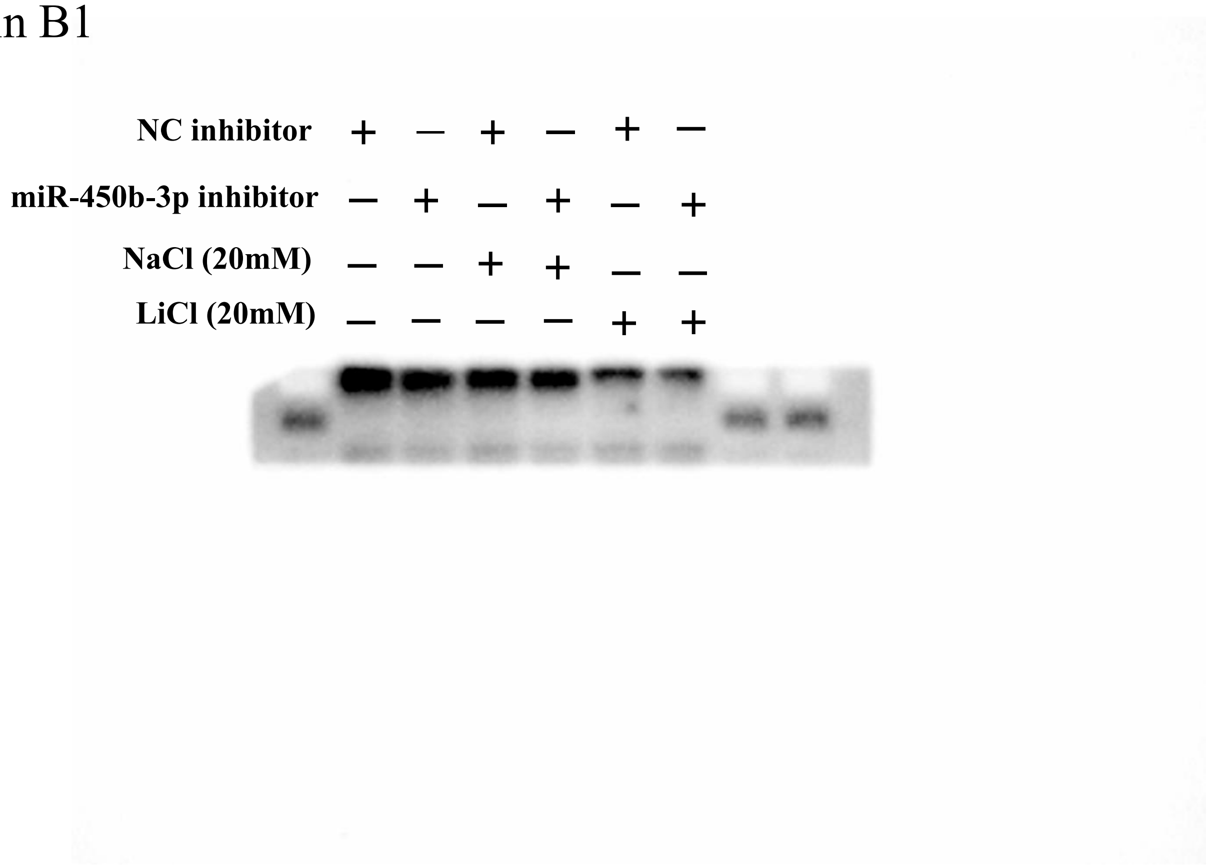

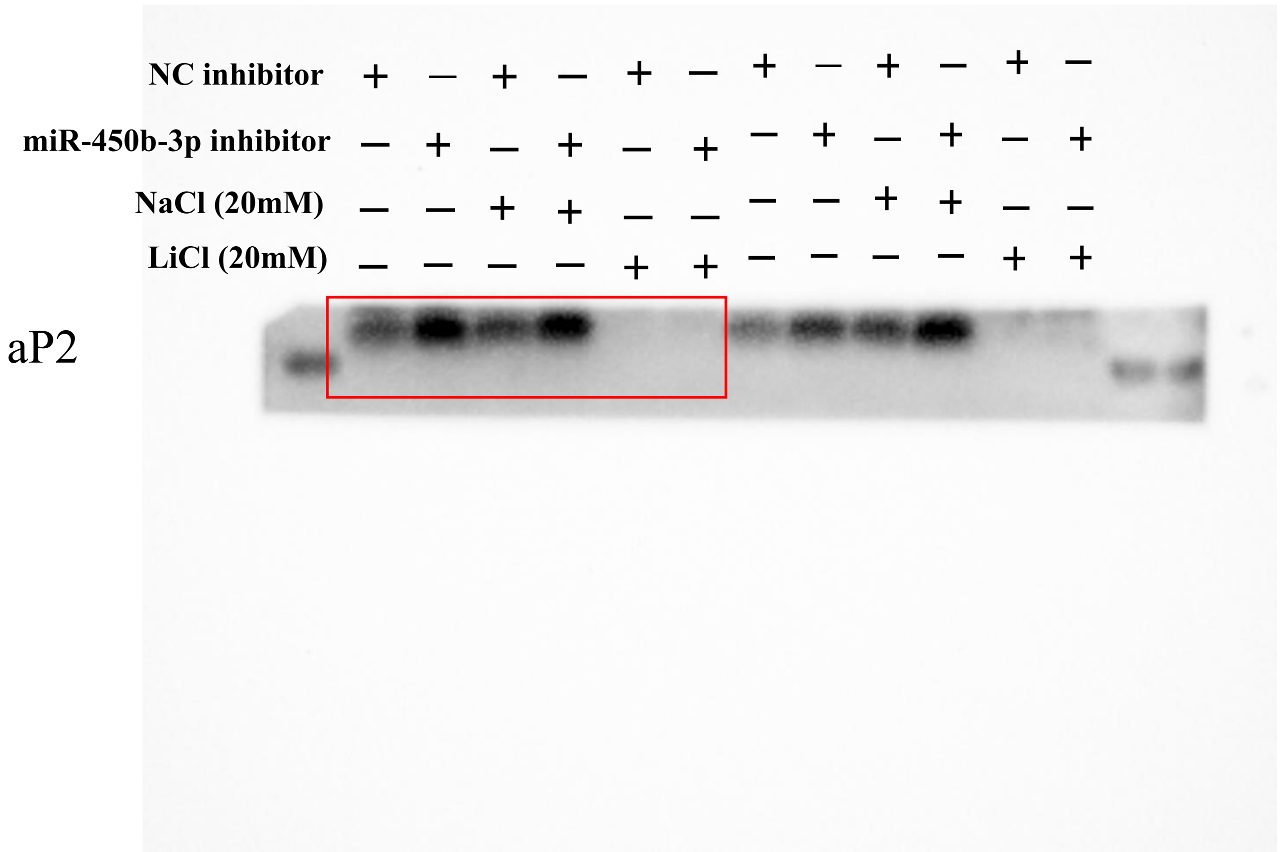

NC inhibitor + – + – + – + – + – + –
miR-450b-3p inhibitor – + – + – + – + – + – +
NaCl (20mM) – – + + – – – – + + – –
LiCl (20mM) – – – – + + – – – – + +
aP2

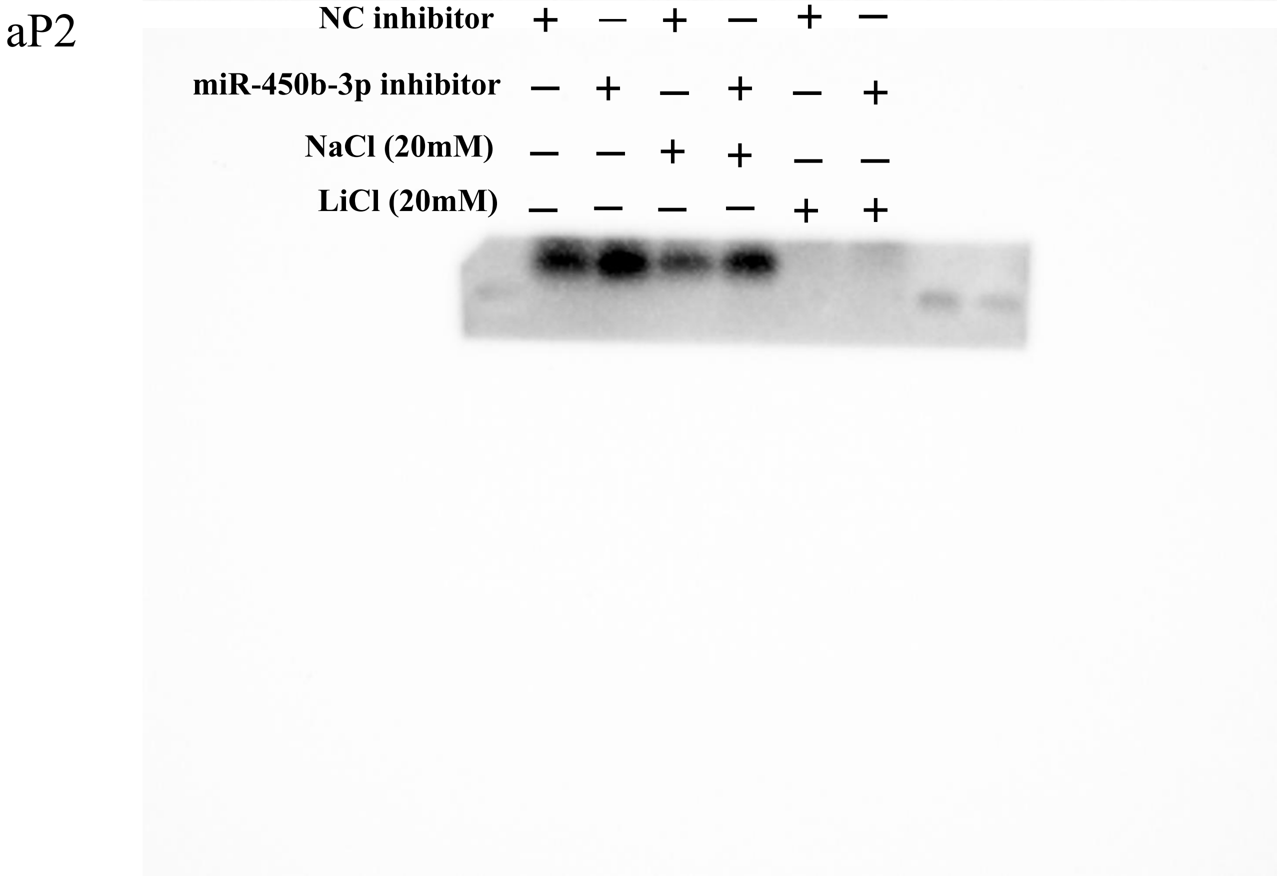

aP2
NC inhibitor + – + – + –
miR-450b-3p inhibitor – + – + – +
NaCl (20mM) – – + + – –
LiCl (20mM) – – – – + +

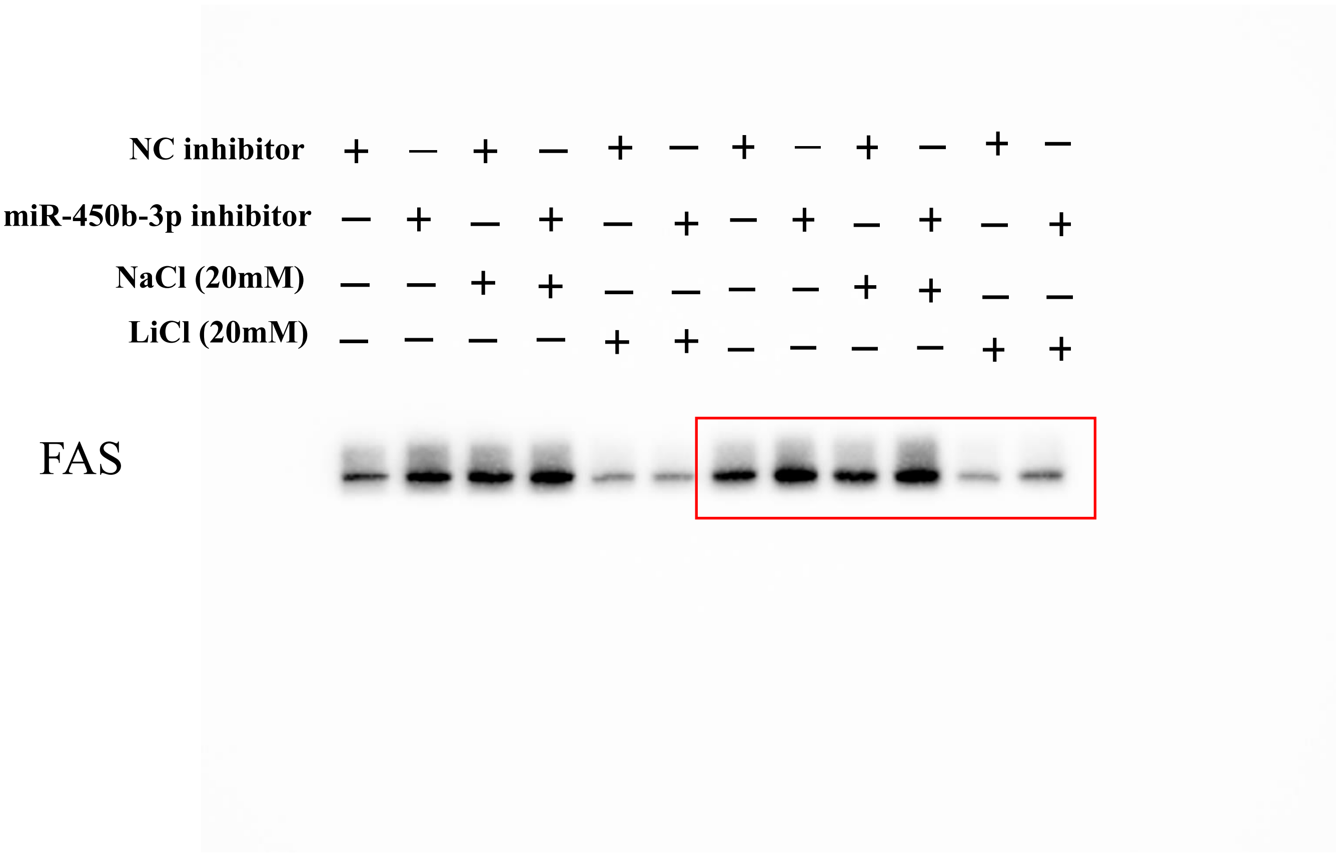
NC inhibitor + – + – + – + – + – + –
miR-450b-3p inhibitor – + – + – + – + – + – +
NaCl (20mM) – – + + – – – – + + – –
LiCl (20mM) – – – – + + – – – – + +
FAS

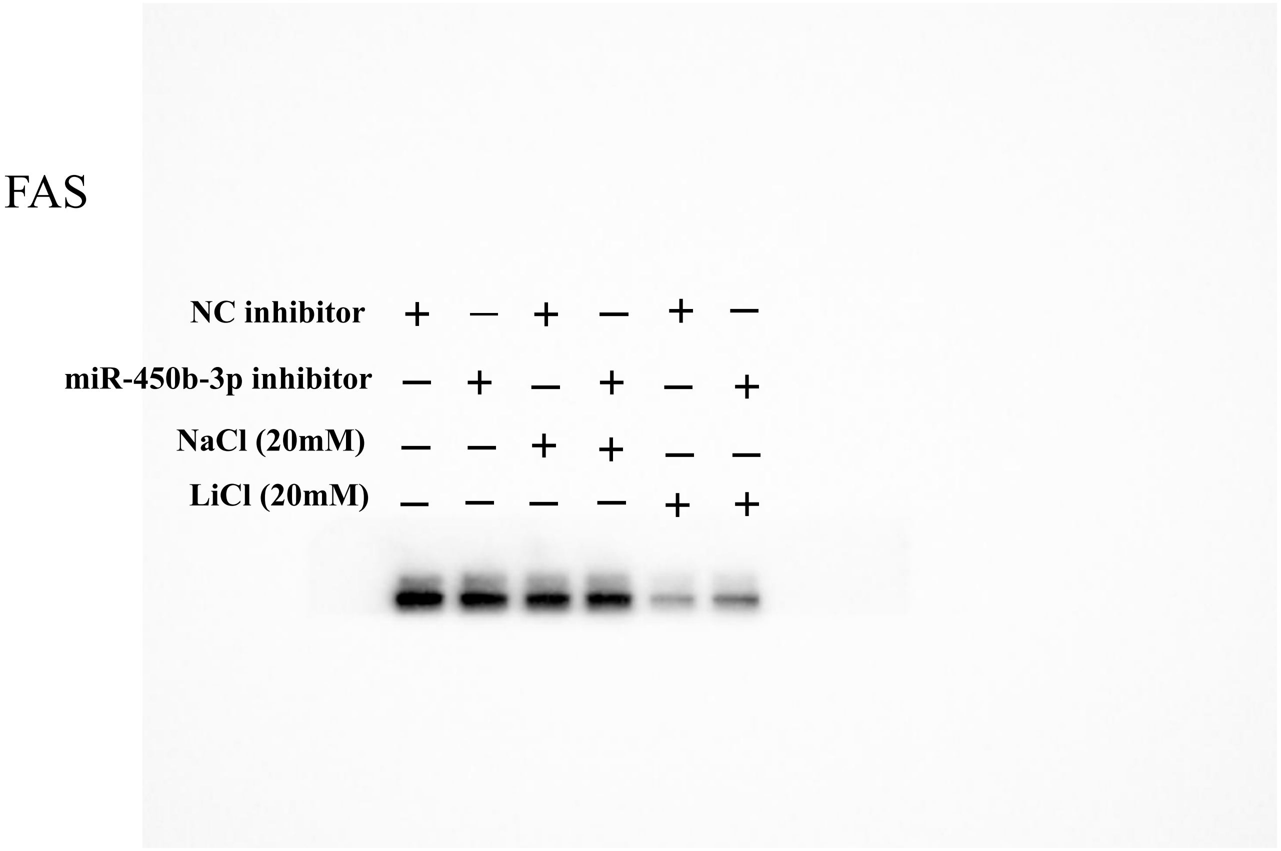
FAS
NC inhibitor + – + – + –
miR-450b-3p inhibitor – + – + – +
NaCl (20mM) – – + + – –
LiCl (20mM) – – – – + +

GAPDH

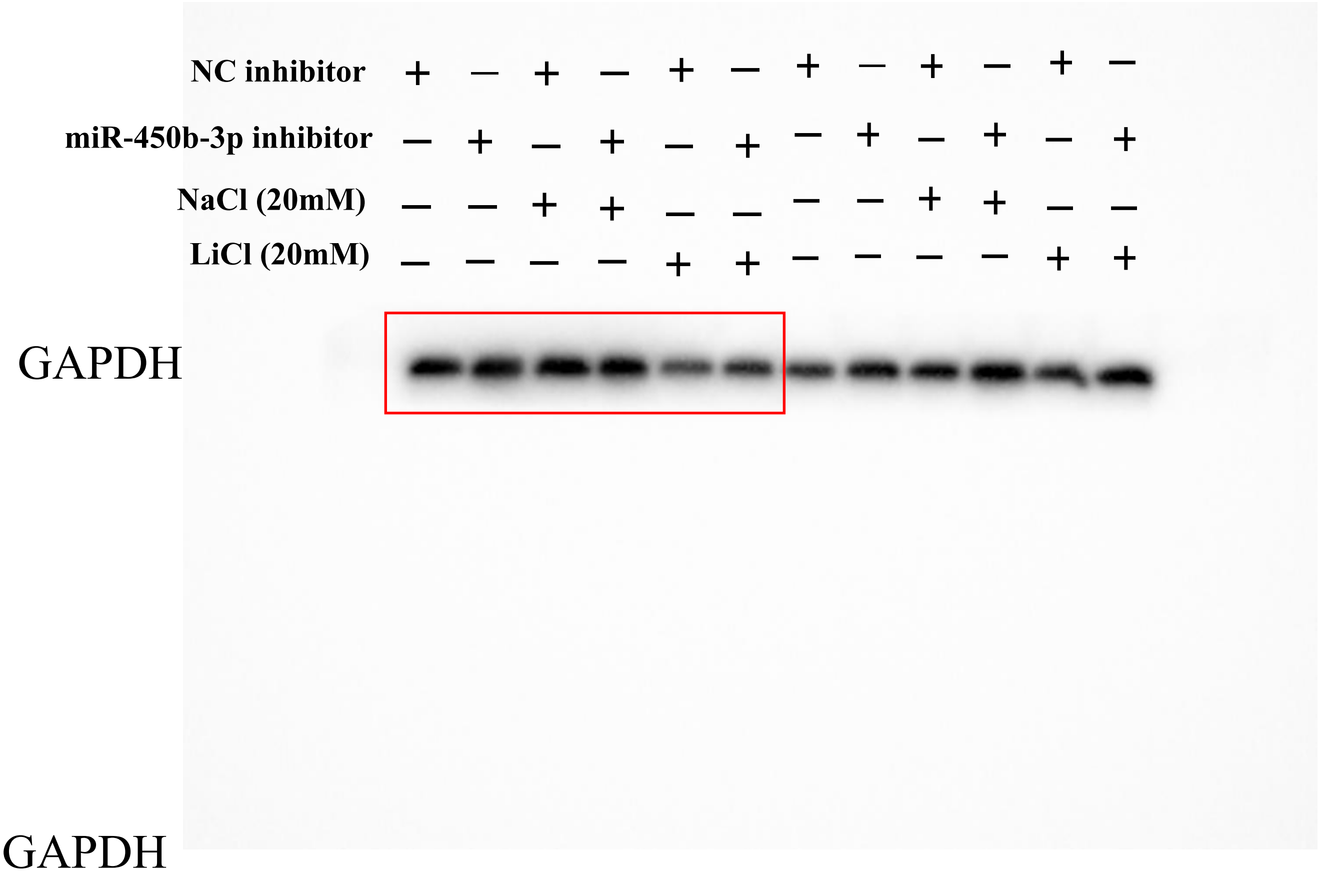
NC inhibitor + – + – + – + – + – + –
miR-450b-3p inhibitor – + – + – + – + – + – +
NaCl (20mM) – – + + – – – – + + – –
LiCl (20mM) – – – – + + – – – – + +

GAPDH

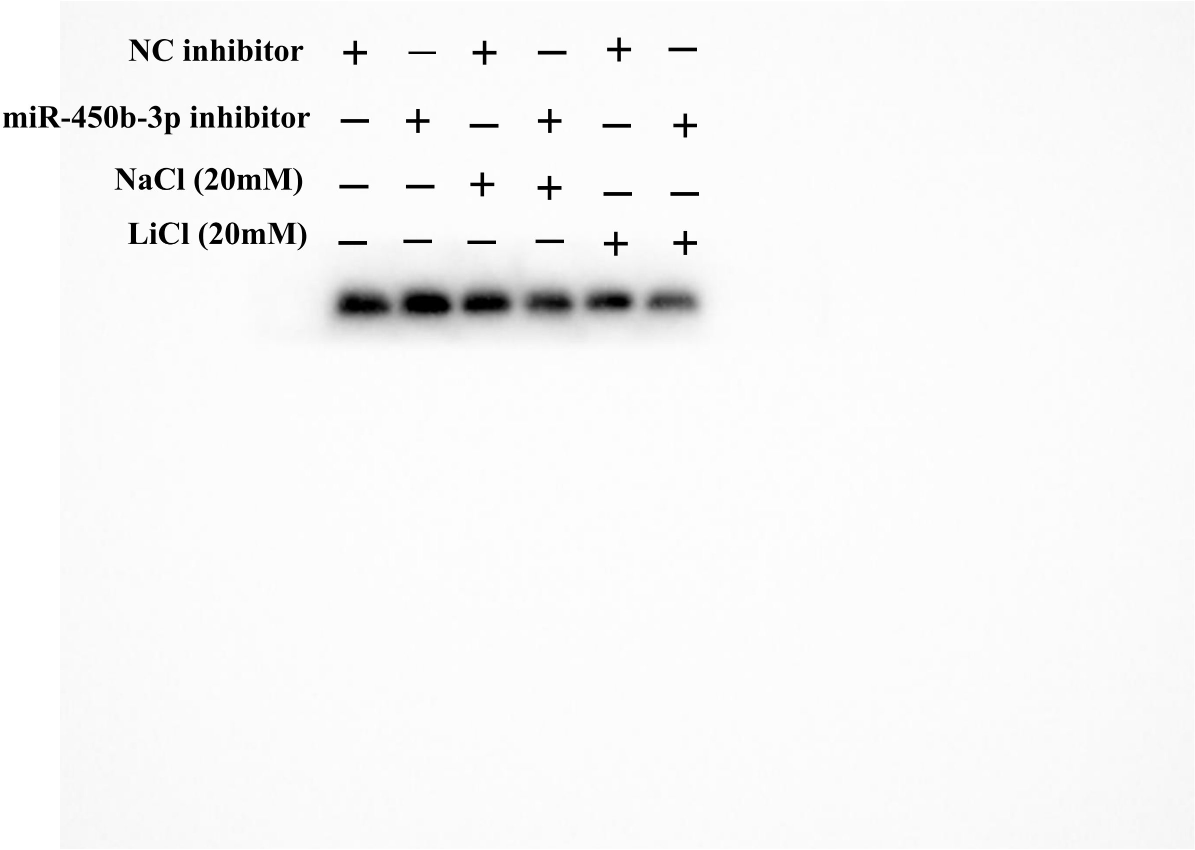
NC inhibitor + – + – + –
miR-450b-3p inhibitor – + – + – +
NaCl (20mM) – – + + – –
LiCl (20mM) – – – – + +

Figure 8

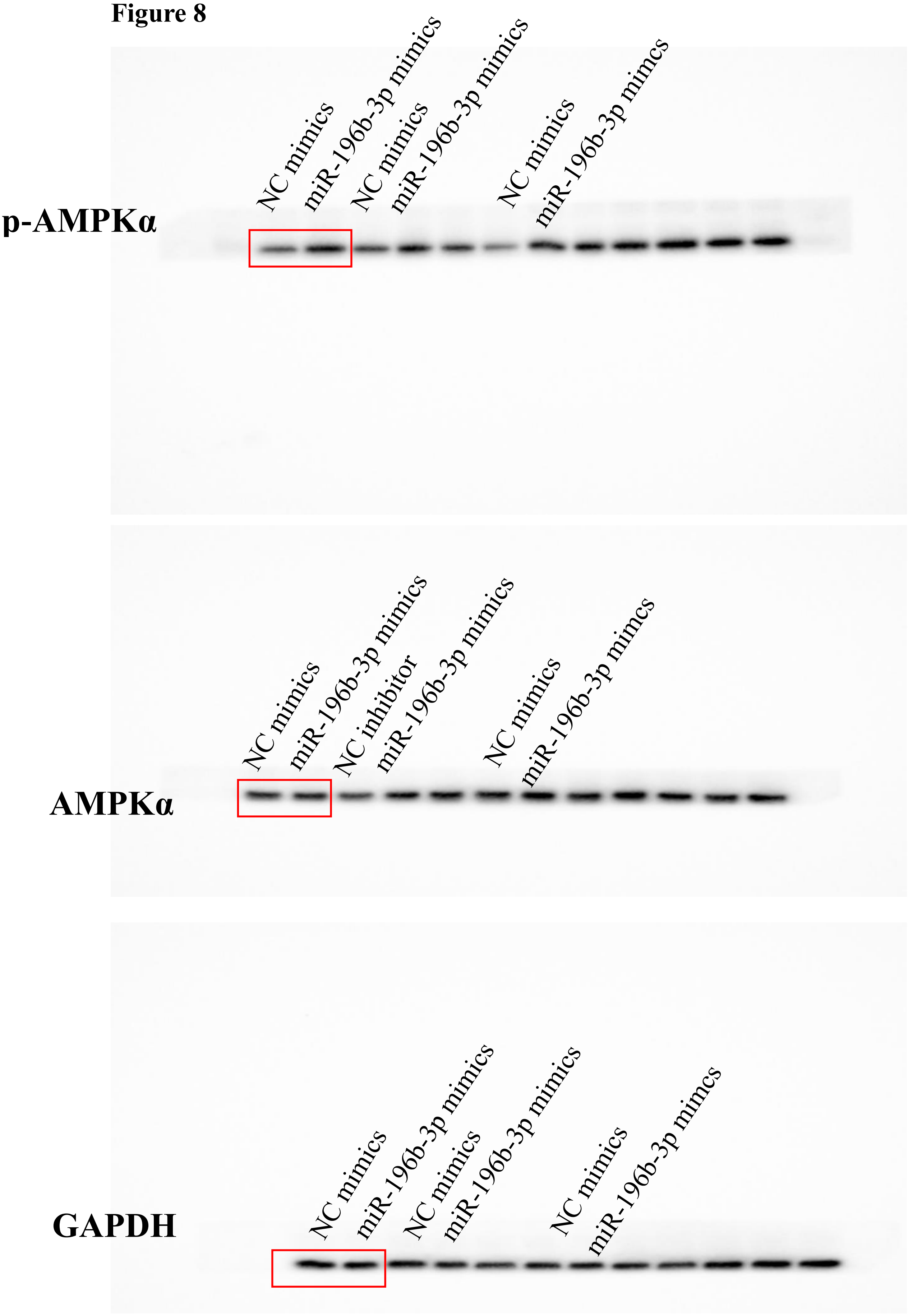

NC mimics
miR-196b-3p mimics
NC mimics
miR-196b-3p mimics
NC mimics
miR-196b-3p mimcs
p-AMPKα
NC mimics
miR-196b-3p mimics
NC inhibitor
miR-196b-3p mimics
NC mimics
miR-196b-3p mimcs
AMPKα
NC mimics
miR-196b-3p mimics
NC mimics
miR-196b-3p mimics
NC mimics
miR-196b-3p mimcs
GAPDH

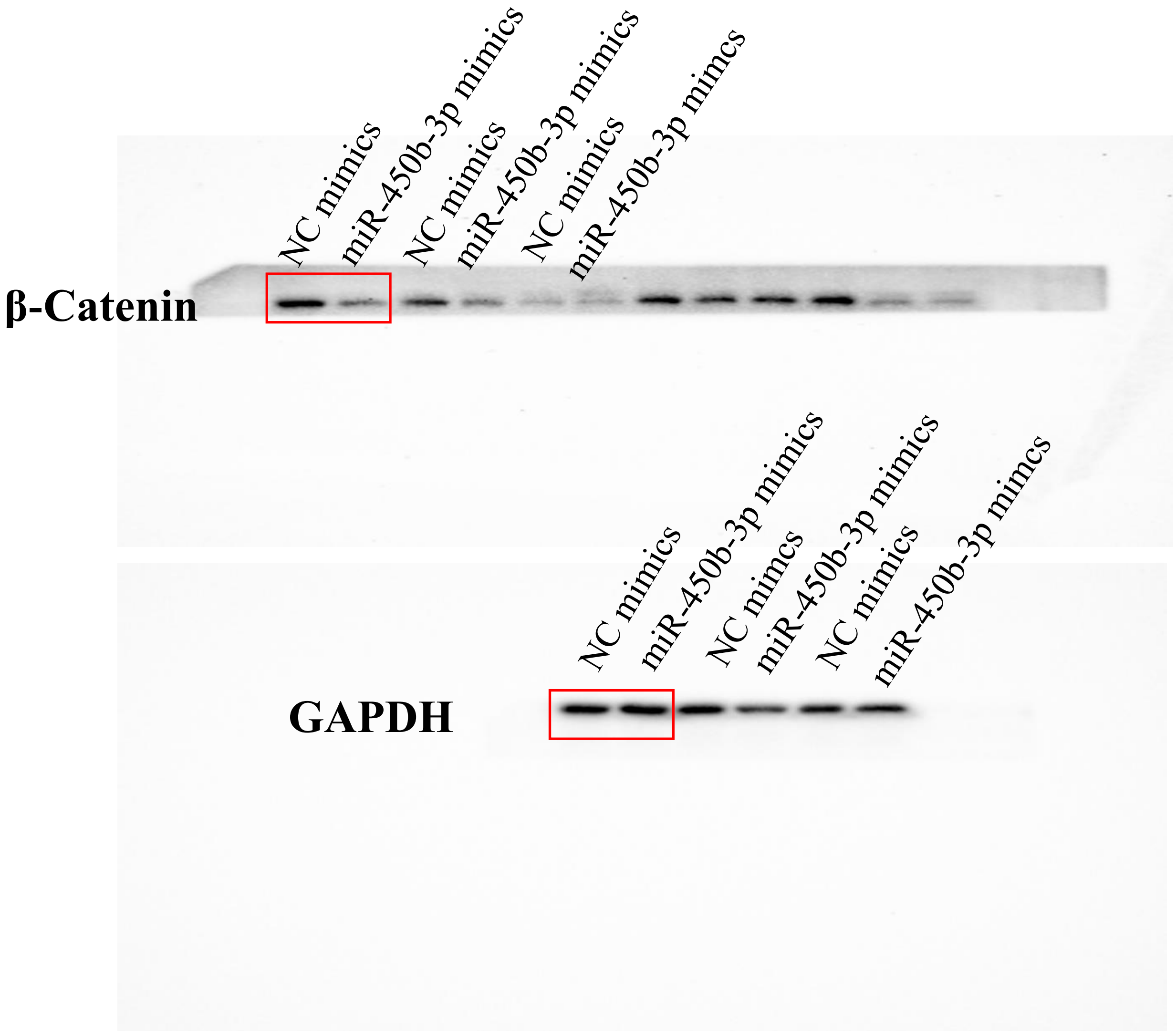
NC mimics
miR-450b-3p mimics
NC mimics
miR-450b-3p mimics
NC mimics
miR-450b-3p mimcs
β-Catenin
NC mimics
miR-450b-3p mimics
NC mimcs
miR-450b-3p mimics
NC mimics
miR-450b-3p mimcs
GAPDH

Figure S1

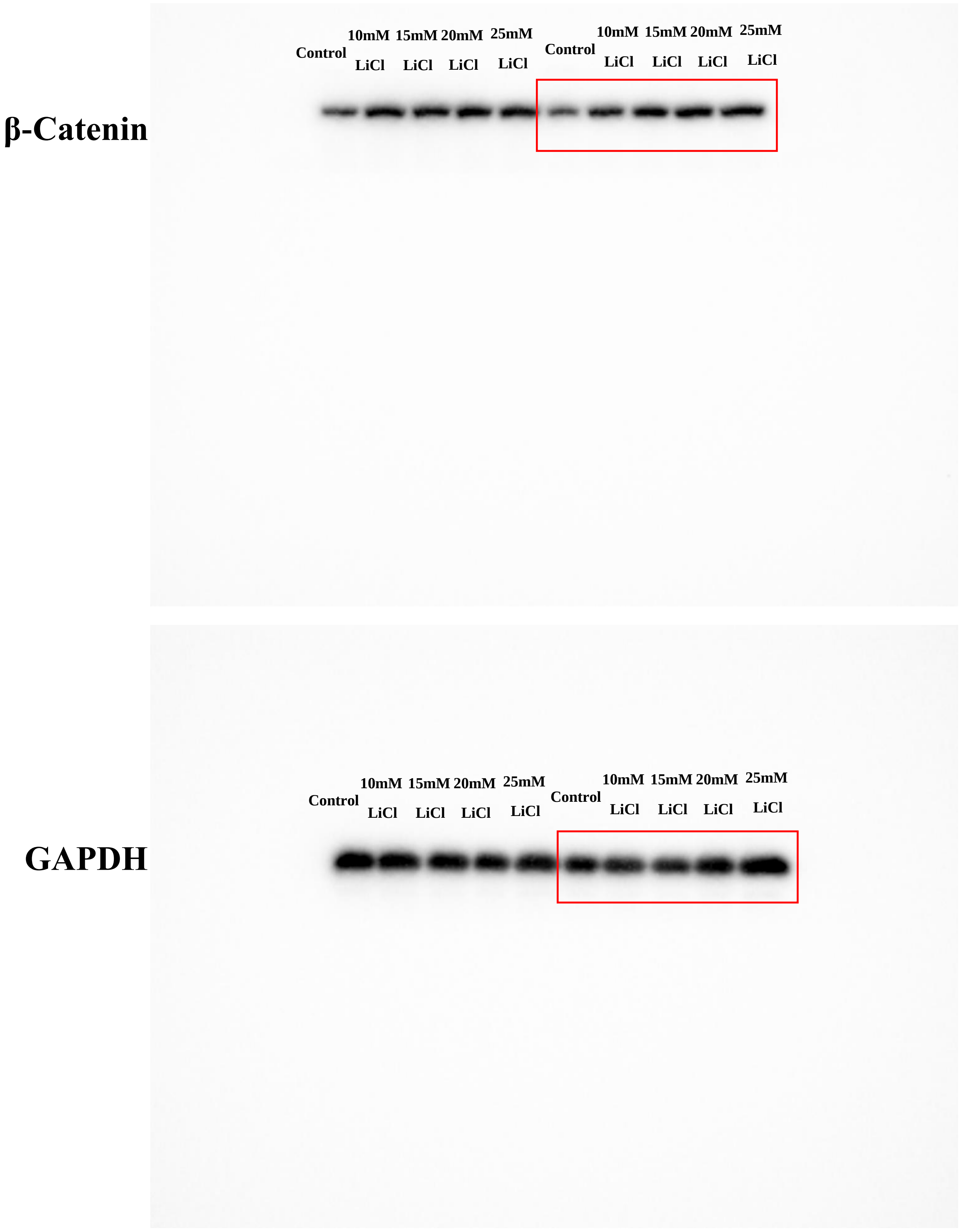

β-Catenin
Control
10mM LiCl
15mM LiCl
20mM LiCl
25mM LiCl
Control
10mM LiCl
15mM LiCl
20mM LiCl
25mM LiCl
GAPDH
Control
10mM LiCl
15mM LiCl
20mM LiCl
25mM LiCl
Control
10mM LiCl
15mM LiCl
20mM LiCl
25mM LiCl
