# Supplementary material for: MiR-196b-3p and miR-450b-3p are key regulators of adipogenesis in porcine intramuscular and subcutaneous adipocytes
Source: BMC Genomics. 2023 Jun 27;24:360. doi: 10.1186/s12864-023-09477-0 (PMC10303896; doi:10.1186/s12864-023-09477-0)
Supplement: Supplementary file 3 — Supplementary Material 3 [file 12864_2023_9477_MOESM3_ESM.pdf]

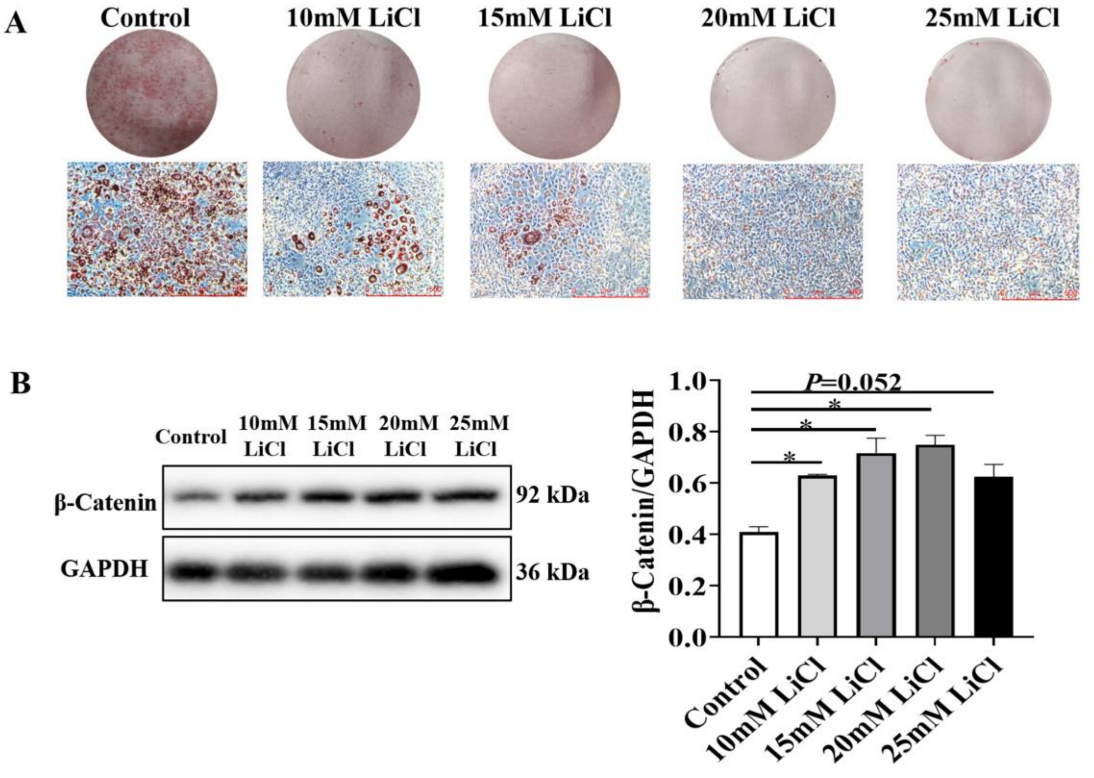

Supplementary Fig. 1 Optimization of the LiCl concentration. Treatment of 3T3-L1 adipocytes with different concentrations of LiCl, (**A**) adipogenic differentiation was detected by oil red O staining. (**B**) The protein level of β-Catenin was determined by western blot analysis. The data represent the means ± SEM. n=2, *$P < 0.05$, **$P < 0.01$
